# Subdivisions of the adult zebrafish pallium based on molecular marker analysis

**DOI:** 10.12688/f1000research.5595.2

**Published:** 2015-11-04

**Authors:** Julia Ganz, Volker Kroehne, Dorian Freudenreich, Anja Machate, Michaela Geffarth, Ingo Braasch, Jan Kaslin, Michael Brand

**Affiliations:** 1 Biotechnology Center, and DFG-Research Center for Regenerative Therapies Dresden, Technische Universität Dresden, Dresden, 01307, Germany; 2 Institute of Neuroscience, University of Oregon, Eugene, OR 97403, USA; 3 Present address: Faculty of Medicine, Nursing Health Sciences Monash University, Clayton, Victoria, 3800, Australia

**Keywords:** telencephalon, teleost, Actinopterygii, amygdala, hippocampus, neuroanatomy, vertebrate brain, homology, evolution, neurogenesis

## Abstract

**Background**: The telencephalon shows a remarkable structural diversity among vertebrates. In particular, the everted telencephalon of ray-finned fishes has a markedly different morphology compared to the evaginated telencephalon of all other vertebrates. This difference in development has hampered the comparison between different areas of the pallium of ray-finned fishes and the pallial nuclei of all other vertebrates. Various models of homology between pallial subdivisions in ray-finned fishes and the pallial nuclei in tetrapods have been proposed based on connectional, neurochemical, gene expression and functional data. However, no consensus has been reached so far. In recent years, the analysis of conserved developmental marker genes has assisted the identification of homologies for different parts of the telencephalon among several tetrapod species.

**Results**: We have investigated the gene expression pattern of conserved marker genes in the adult zebrafish ( *Danio rerio)* pallium to identify pallial subdivisions and their homology to pallial nuclei in tetrapods. Combinatorial expression analysis of *ascl1a*, *eomesa*, *emx1*, *emx2*, *emx3*, and Prox1 identifies four main divisions in the adult zebrafish pallium. Within these subdivisions, we propose that Dm is homologous to the pallial amygdala in tetrapods and that the dorsal subdivision of Dl is homologous to part of the hippocampal formation in mouse. We have complemented this analysis be examining the gene expression of *emx1*, *emx2* and *emx3* in the zebrafish larval brain.

**Conclusions**: Based on our gene expression data, we propose a new model of subdivisions in the adult zebrafish pallium and their putative homologies to pallial nuclei in tetrapods. Pallial nuclei control sensory, motor, and cognitive functions, like memory, learning and emotion. The identification of pallial subdivisions in the adult zebrafish and their homologies to pallial nuclei in tetrapods will contribute to the use of the zebrafish system as a model for neurobiological research and human neurodegenerative diseases.

## Background

The functions of the different parts of the telencephalon encompass control of sensory and motor, autonomic and endocrine functions, as well as cognitive tasks like memory, learning and emotion. The structures in the telencephalon can be assigned either to its dorsal part, the pallium, or its ventral part, the subpallium ^1– 3^. The telencephalon of most vertebrates forms by an evagination of the neural tube, where the central lumen of the neural tube expands to form the two paired telencephalic vesicles ^1, 3, 4^. However, in ray-finned fishes the rostral neural tube is thought to bend outward resulting in two telencephalic hemispheres separated by an unpaired ventricle and covered by a thin roof plate, thus referred to as “everted” ^1, 3, 4^. In the rayfin teleost zebrafish, it has been shown that the morphogenetic movement that creates this different layout of the telencephalon does not result from a simple lateral outward bending of the telencephalic walls ^5^. Instead, telencephalon morphogenesis comprises first the generation of a ventricular outfolding between telencephalon and diencephalon, followed by an enlargement of the pallial territory rostrally ^5^. The different development of the telencephalon results in an unpaired ventricle and a different arrangement of the parts in the pallium compared to all other vertebrates ^1, 4, 6^. Hence, due to its everted nature, a comparison between the parts of the pallium of rayfin fishes and all other vertebrates has been difficult. The correct determination of homologous pallial areas between teleosts and tetrapods is critical for the usage of teleost fish as neurobiological models as well as models for human neurological diseases. In recent years, a variety of different studies have demonstrated that using the expression pattern of conserved developmental regulatory genes as landmarks is a useful approach to identify homologous subdivisions of brain regions between divergent vertebrate species. The advantage of using the expression of conserved developmental genes lies in the uncoupling of anatomical and developmental differences of the brain part of interest between divergent species. This approach has been especially valuable for the telencephalon with its great variability in morphology between vertebrate species and has led to the clarification of the homology within subdivisions of the telencephalon between different vertebrate species, such as the domestic mouse, the chicken and the African clawed frog (e.g. ^7– 22^). For example, the gene expression of *Tbr1* and *Eomes* ( *Tbr2*) has been successfully used to identify the extent of the pallium in tetrapod embryos ^9, 21, 23^. In addition, absence of *Emx1* expression and presence of *Tbr1* expression delineate the ventral pallium in tetrapod embryos ^9, 10, 12, 13, 21^.

The pallium in teleosts has generally been subdivided in a medial (Dm), dorsal (Dd), central (Dc), lateral (Dl) and posterior (Dp) part ^4^. The pallium shows a notable structural variety and different subdivisions of these broad divisions have been described in different teleost species ^3, 24– 29^. The subdivisions of the pallium and their homologies to nuclei in other vertebrate species have not been resolved, but different models based on neurochemical and connectional data have been suggested ^3, 29– 38^. Ablation experiments combined with behavioral experiments suggest that the lateral nucleus of the pallium shows a similarity in function to the hippocampus and the medial nucleus of the pallium to the amygdala of amniotes ^39– 43^. Based on the expression of nicotine adenine dinucleotide phosphate diphorase (NADPHd) and Parvalbumin, a new model of four subdivisions (Dm, Dc, Dl, and Dp) has been proposed for the adult zebrafish pallium ^32^. However, a comprehensive study of pallial subdivisions based on different conserved molecular markers is still missing in the adult zebrafish.

The object of this study was to analyze expression of conserved marker genes to identify subdivisions within the adult zebrafish pallium. Here, we investigated the expression patterns of the molecular marker genes *emx1*, *emx2*, *emx3* to identify a ventral pallial subdivision both in the larval and adult zebrafish pallium. The expression of Prox1 in a dorsal subdivision of Dl caudally suggests that it is homologous to the dentate gyrus in mouse. Combinatorial expression of *ascl1a*, *emx1*, *emx2*, *emx3,* and *eomesa* shows four main divisions in the pallium, Dm, Dc, Dl, and Dp. The combinatorial expression pattern also suggests a subdivision of Dl in a dorsal and ventral subdivision (which we have named Dld and Dlv, respectively).

## Material and methods

### Fish maintenance

Fish were kept under standard conditions at a 14 hours light/10 hours dark cycle as previously described ^44, 45^. All procedures were in accordance with the live animal handling and research regulations of the local Animal Care and Use Committee, the Regierungspräsidium Dresden (permit AZ 24D-9168.11-1/2008-1 and -4). Wildtype experimental animals (Biotechnology Center Dresden) were adult fish from the *gol-b1* line in the AB genetic background ^46^. Adult fish were 6–8 months old and had a 24mm–32mm body length, zebrafish larvae were 7dpf old.

### Tissue preparation

Brains (either dissected or within the skull) were fixed at 4°C overnight in 2–4% paraformaldehyde/0.1M phosphate buffer (PB), pH 7.5. They were washed 1 × 10 minutes and then up to 1h in 0.1M PB and subsequently transferred for decalcification and cryoprotection to 20% sucrose/20% EDTA in 0.1M PB, pH7.5. Brains were frozen in 7.5% gelatine/20% sucrose and sectioned into 14–16 µm cryosections. Sections were stored at -20°C.

### Orthology analysis between tetrapod and teleost genes

Compared to tetrapods, teleost fish have undergone an additional whole genome duplication: the teleost genome duplication (TGD) (reviewed in ^47^). Thus, there is the possibility of two co-orthologous genes in zebrafish compared to the single tetrapod gene.

**Figure S1.**
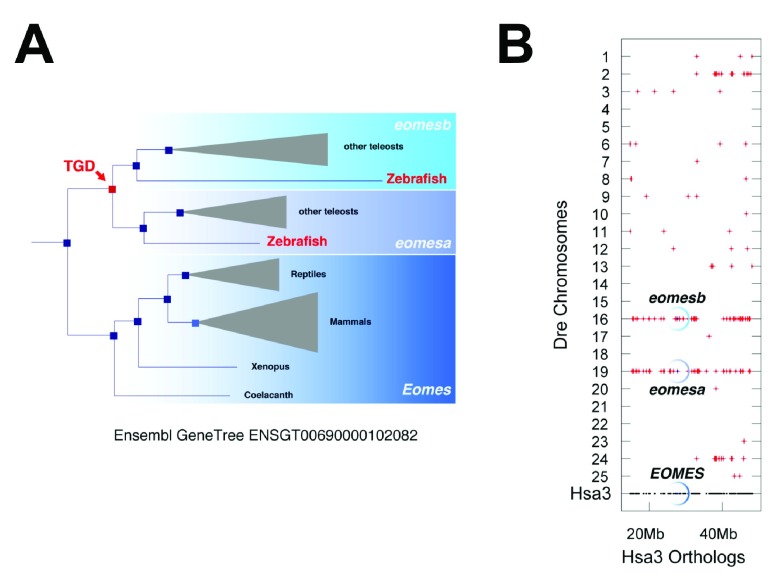
Teleost *eomes* genes. **A**. The topology of the Ensembl Gene Tree suggest that the teleost *eomes* co-orthologs, *eomesa* and *eomesb*, were duplicated at the base of the teleost lineage and thus most likely during the teleost genome duplication (TGD). **B**. Dotplot from the Synteny Database shows that the human *EOMES* gene region on chromosome Hsa3 shows extensive double conserved synteny to zebrafish chromosomes Dre19 and Dre16 which contain *eomesa* and *eomesb*, respectively, providing strong evidence for the TGD origin of teleost *eomes* co-orthologs.

We analyzed if there are two co-orthologous genes compared to the tetrapod gene using Ensembl73 gene trees ( http://www.ensembl.org) and synteny analysis with the Synteny Database ( http://syntenydb.uoregon.edu; ^48^). This is clearly the case, e.g., for human *EOMES* with two TGD co-orthologs in zebrafish, *eomesa* and *eomesb* ( Figure S1A,B).

Two genes are currently termed *ascl1* in zebrafish. Zebrafish *ascl1a* is clearly orthologous by phylogeny and conserved synteny to *Ascl1* in lobefins (tetrapods and coelacanth). Zebrafish *ascl1b* not only has a separate ortholog in coelacanth but also shows conserved synteny to tetrapod *Ascl2*, suggesting that *ascl1b* is in fact the missing teleost *ascl2* gene.

There are two *prox1* genes described in zebrafish, yet while *prox1a* is clearly orthologous to tetrapod *Prox1*, teleost *prox1b* shows no conserved synteny to teleost *prox1a* or tetrapod *Prox1*. This suggests that teleost *prox1b* represent a more distant *prox* paralog and is not a TGD paralog of *prox1a*. The phylogeny of the *emx* genes in zebrafish has previously been determined in ^49^.

### RNA *in situ* hybridization

**Figure S2.**
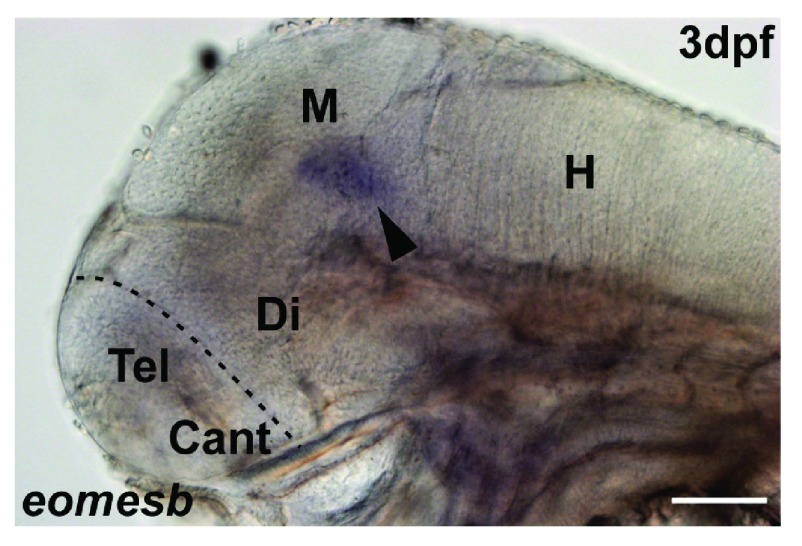
Expression of *eomesb* in the embryonic zebrafish brain. **A**. *eomesb* expression is not found in the telencephalon (T), but present in the midbrain (M), arrowhead, shown is a lateral view. Di Diencephalon, H Hindbrain.

RNA *in situ* hybridization on sections and on whole-mount brains and RNA probe generation was essentially performed as previously described ^7, 50^. Briefly, after defrosting at room temperature (RT), sections were rehydrated for 15 minutes in PBS with 0.3% TritonX (PBSTx) and incubated with the probe overnight at 62–65°C. Information on the antisense *in situ* riboprobes can be found in ^7^ for *ascl1a* (NM_131219), *emx1* (NM_198144), *emx2* (NM_131280), *emx3* (NM_131279), *eomesa* (NM_131679). The *in situ* probe *eomesb* (NM_001083575) was cloned from zebrafish embryonic cDNA with the following primers ( *eomesb*-F, TTTCCAAAACGAAAAGCGTA, *eomesb*-R, GAGCCAGAACTGGATCCTTCT). The *eomesb* probe was tested on 3dpf embryos and showed specific staining only in the midbrain at 3dpf ( Figure S2, Dataset 1). A sense probe of *eomesb* did not show any signal. The sections were washed at 60–65°C in washing solution (1 × SSC, 50% deionized formamide) for 1 × 15 minutes and 2 × 30 minutes followed by 2 × 30 minutes MAB with 0.1% Tween-20 (MABT) washes. Sections were incubated for 1h at RT in 2% DIG-blocking reagent (Roche) and incubated with anti-DIG antibody (Roche Diagnostics, sheep, polyclonal, Fab fragments conjugated to alkaline phosphatase, #11093274910) diluted 1:4000 in 2% DIG-blocking reagent overnight at 4°C. Subsequently, sections were washed 4 × 20 minutes in MABT, equilibrated with staining buffer and stained with the substrate NBT/BCIP. The staining was controlled using a stereomicroscope. Finally, sections were washed 2 × 5 minutes in PBS, postfixed with 4% PFA for 20–30 minutes, washed again 2 × 10 minutes in PBS and mounted with 70% glycerol in PBS. All washing steps were performed on a shaker, all incubation steps in a humid chamber. To test for nonspecific binding of the antibodies that detect digoxigenin, which are not endogenous to vertebrate tissue, we performed control experiments in which the labeled RNA was omitted from the hybridization mix. No signal was detected in the absence of the riboprobe, demonstrating that the antibody reacts specifically with the synthetic RNA ( Dataset 2).

### Immunohistochemistry

Immunohistochemistry on cryosections was performed as previously described ^51^. Briefly, to retrieve the antigens of Prox1, sections were pre-incubated in 50 mM Tris-buffer (pH 8.0) at 99°C for 5 minutes, cooled down to RT over 15 minutes and washed for 5 minutes in PBS and twice for 10 minutes in PBSTx. The sections were then incubated in primary and secondary antibodies in PBSTx. The primary antibody Prox1 (AB5475, 1:2000, Millipore) was incubated overnight at 4°C and secondary antibodies for 1h at room temperature. The slides were washed in PBSTx and mounted. The secondary antibody (dilution 1:750) was Alexa 488-Fluor conjugated (A-11034, Invitrogen, Karlsruhe).

### Image acquisition and processing

Confocal images were acquired with Leica TCS-SP5 confocal microscope. Brightfield images were acquired with Zeiss Axio Imager Z1. The images were processed using ImageJ v. 1.4.3.67 and Adobe Photoshop CS2. Composites were assembled using Adobe Photoshop CS2 and Adobe Illustrator CS2.

### Nomenclature

We primarily followed the nomenclature proposed in ^32^ with modifications based on our combinatorial expression pattern analysis, which suggest a subdivision of Dl in a dorsal and ventral subdivision (which we have named Dld and Dlv, respectively). In the figures we employ a two-color code to separate subdivisions that can be made based on the current marker (red dashed line) from those that we make based on other markers (white dashed line). The black dashed line indicates the boundary between D and V. The distinction of ventricular zone versus neuronal layer was based on cellular morphology. The ventricular zone is the region where cells are directly facing the ventricle. The neuronal layer is characterized by cells with a round shape.

## Results

### Expression of *eomesa* in the adult zebrafish pallium

**Figure 1.**
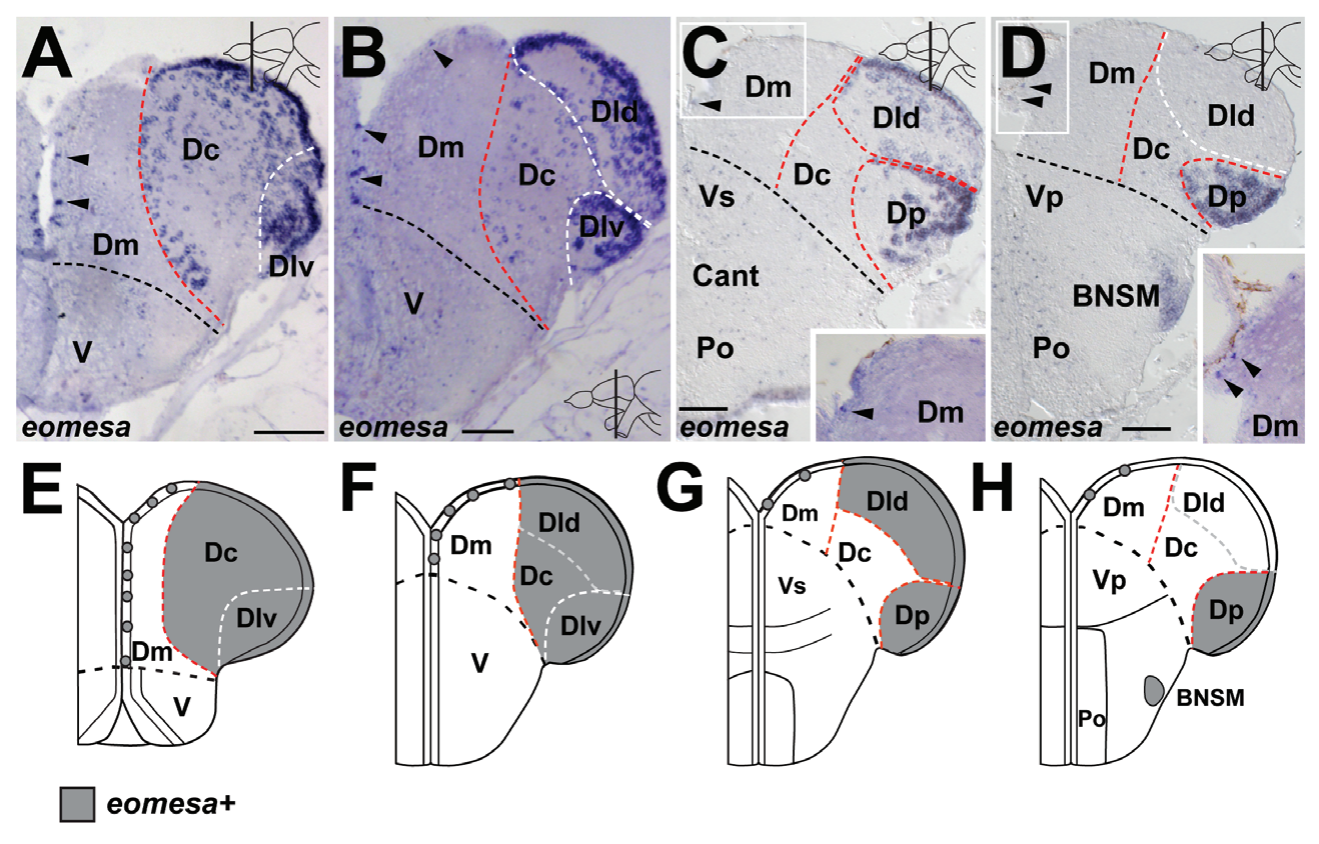
Expression of *eomesa* in the pallium. **A**. In the rostral telencephalic, *eomesa* expression is found in the ventricular zone (vz) of Dm (arrowheads) and in the vz and neuronal layer (nl) of Dc and Dlv. **B**. At mid-telencephalic levels, *eomesa* expression is present in the vz of Dm (arrowheads), in the nl of Dc and in the vz and nl of Dld and Dlv. **C**. At the anterior commissure (Cant), *eomesa* expression is present in the vz of Dm (arrowhead) and in the vz and nl of Dld and Dp. Inset shows close-up of vz of Dm **D**. Caudal to Cant, *eomesa* expression is present in the vz of Dm (arrowhead), in the vz and nl of Dp and in BNSM. Inset shows close-up of vz of Dm **E**.– **H**. Summary of the expression pattern of *eomesa* at rostral ( **E**.), mid-telencephalic ( **F**.), commissural ( **G**.) and postcommissural levels ( **H**.). **A**.– **D**. Brightfield images of cross-sections at the levels indicated through the telencephalon. Red dashed line indicates subdivisions based on the current marker. White dashed line indicates subdivisions based on other markers. The black dashed line indicates the boundary between D and V. Scale bars = 50µm in **A**– **D**.

In tetrapod embryos, *Eomes* ( *Tbr2*) expression is found in the ventricular zone and mantle layer in all parts of the pallium at embryonic stages ^9, 21, 23^. In the zebrafish embryo, *eomesa* expression is present throughout the dorsal telencephalon ^52, 53^. Its TGD paralog, *eomesb* is not present in the embryonic or adult telencephalon ( Figure S2, Dataset 1) and thus was not further taken into account. In the adult zebrafish, *eomesa* positive cells are scattered in a salt-and-pepper pattern along the ventricular zone of Dm along the rostro-caudal axis ( Figure 1A–D, arrowheads). Further, *eomesa* expression is present in the ventricular zone and neuronal layer of Dc, Dlv and Dld in the rostral telencephalon and at mid-telencephalic levels and in the ventricular zone and neuronal layer of Dld and Dp at the anterior commissure (Cant; Figure 1A–C). Caudal to Cant, *eomesa* is expressed in the ventricular zone and neuronal layer of Dp, sporadically in Dld and in bed nucleus of the stria medullaris (BNSM, Figure 1D). In summary, *eomesa* is differentially expressed in the pallium along the rostro-caudal axis ( Figure 1E–H, Dataset 3; Table 1). The most abundant parenchymal *eomesa* expression is detected in parts of the central and lateral dorsal telencephalic nuclei.

**Table 1.** Summary of gene expression pattern in the adult zebrafish pallium.

	*ascl1a*	*eomesa*	*emx1*	*emx2*	*emx3*	**Prox1**
**Dm**	3 vz ^a^	3 vz ^a^	0	0	3vz nl	0
**Dc**	0	3 vz nl/0 ^b^	0	0/3 nl ^c^	1–2 nl ^d^	0
**Dld**	0	3 vz nl ^e^	0	0	0	0/3 nl ^f^
**Dlv**	2 vz ^a^	3 vz nl	0	0	2 vz nl ^g^	0
**Dp**	2 vz ^a^	3 vz nl	3 vz nl	0	2 vz nl	0
**EN**	0	0	3 nl	0	0	0
**BNSM**	0	3 nl	0	0	0	0

0 expression not detected, 1 weak expression, 2 moderate expression, 3 strong expression, vz ventricular zone, nl neuronal layer,

^a^ scattered cells in the ventricular zone; ^b^ no expression from mid-telencephalic levels; ^c^ part of Dc posterior to Cant; ^d^ scattered cells in the neuronal layer; ^e^ no expression posterior to Cant; ^f^ expression at mid-telencephalic level shortly anterior to Cant, shortly caudal to Cant, only scattered Prox1+ cells are present; ^g^ expression present in the rostralmost telencephalon in the vz, moving caudally expression in vz and nl.

### Expression of *emx1*, *emx2* and *emx3* in the adult zebrafish pallium

**Figure 2.**
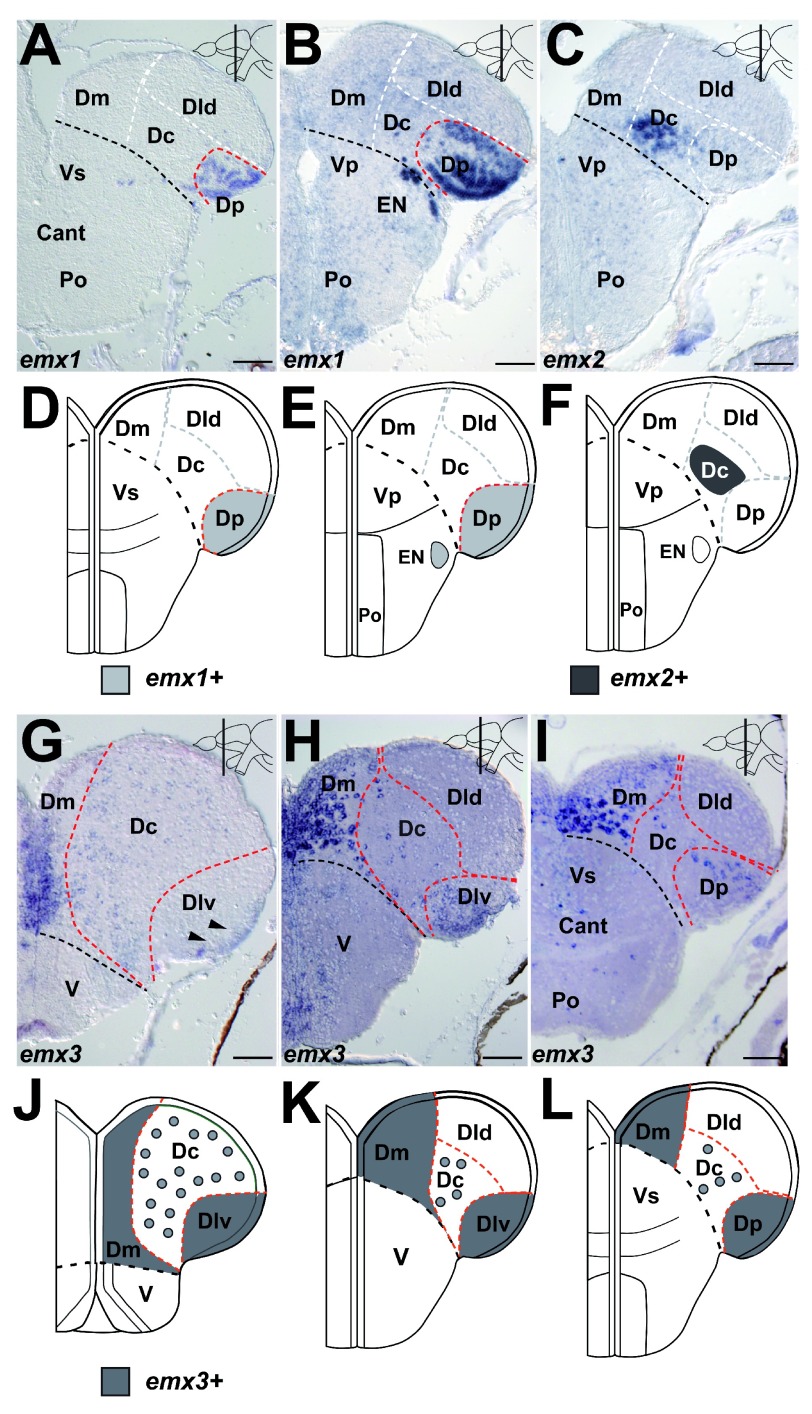
Expression of *emx1*, *emx2* and *emx3* in the pallium. **A**. At the anterior commissure (Cant), *emx1* expression is found in neuronal layer (nl) and ventricular zone (vz) of Dp. **B**. Caudal to Cant, *emx1* expression is present in the vz and nl of Dp and in EN. **C**. Caudal to Cant, *emx2* expression is present in part of Dc. **D**.– **F**. Summary of the expression pattern of *emx1* and *emx2* at commissural ( **D**.) and postcommissural levels ( **E**., **F**.). **G**. In the rostral telencephalon, *emx3* expression is present in the vz and nl of Dm, weakly in scattered cells of the nl od Dc and in the vz of Dlv (arrowheads). **H**. At mid-telencephalic levels, *emx3* expression is found in the vz and nl of Dm, in scattered cells in Dc, and in the vz and nl of Dlv. **I**. At Cant, *emx3* expression is present in the vz and nl of Dm, in scattered cells in Dc and in the vz and nl of Dp. **J**.– **L**. Summary of the expression pattern of *emx3* at rostral ( **J**.), mid-telencephalic ( **K**.) and commissural levels ( **L**.). **A**.– **F**. Brightfield images of cross-sections at the levels indicated through the telencephalon. Red dashed line indicates subdivisions based on the current marker. White dashed line indicates subdivisions based on other markers. The black dashed line indicates the boundary between D and V. Scale bars = 50µm in **A**– **D**.

**Figure S3.**
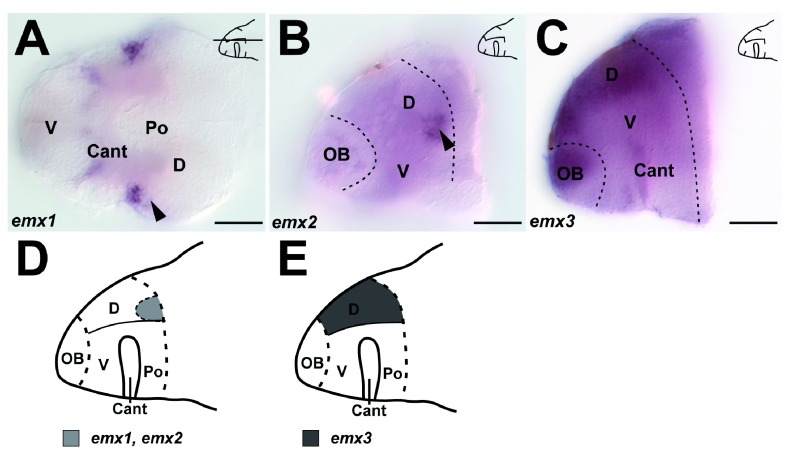
Expression of *emx1*, *emx2* and *emx3* in the zebrafish larval brain at 7dpf. **A**. *emx1* expression is present in the lateral part of the dorsal telencephalon ( **D**, arrowhead), shown is a ventral view. **B**. *emx2* expression is present in the lateral part of D (arrowhead), shown is a parasaggital view. **C**. *emx3* expression is present throughout **D**, shown is a parasaggital view. **D**. Summary of the distribution of *emx1* and *emx2* expression in **D** in a sagittal view. **E**. Summary of the distribution of *emx3* expression in **D** in a sagittal view. **A**.– **C**. Brightfield whole-mount images, **A**. ventral, **B**.– **C**. sagittal views. Scale bars = 50µm **A**– **C**.

In tetrapod embryos, the ventral pallial subdivision is characterized by absence of *Emx1* and presence of *Tbr1* ^9, 10, 12, 13, 21^. In embryonic and adult zebrafish, *tbr1* is expressed throughout the pallium ^7, 53, 54^. Thus, we investigated the expression of *emx1, emx2* and *emx3* to identify a ventral pallial subdivision in the zebrafish pallium. In zebrafish, the three *emx* genes are expressed at 1 day post fertilization (dpf) in the embryo throughout the dorsal telencephalon ^49, 55, 56^. In 7 day-old larva, the expression of *emx1* and *emx2* is restricted to a small caudo-lateral area in the dorsal telencephalon ( Figure S3A,B,D, Dataset 4). The expression of *emx3* is found throughout the dorsal telencephalon ( Figure S3C,E, Dataset 4). In the adult zebrafish pallium, *emx1* and *emx2* are expressed in a very restricted fashion. The expression of *emx1* only starts in the mid-telencephalon shortly rostral to Cant, where it is restricted to the ventricular zone and neuronal layer of Dp. This expression pattern continues to Cant ( Figure 2A). Furthermore, *emx1* expression is present in Dp and in EN caudal to Cant ( Figure 2B). The expression of *emx2* is only found caudal to Cant in an area in Dc lateral to Vp ( Figure 2C). Similar to the larvae, *emx3* shows the broadest expression of the *emx* genes. In the rostral telencephalon, the expression of *emx3* is found in the ventricular zone and neuronal layer of Dm and weakly in scattered cells in the neuronal layer of Dc ( Figure 2G). Additionally, the expression of *emx3* is weakly present rostrally in the ventricular zone of Dlv ( Figure 2G, arrowheads). At mid-telencephalic levels, *emx3* expression is found in the ventricular zone and neuronal layer of Dm, in scattered cells in Dc and in the ventricular zone and neuronal layer of Dlv ( Figure 2H). At Cant, expression of *emx3* is found in the ventricular zone and neuronal layer of Dm, in scattered cells in Dc and in the ventricular zone and neuronal layer of Dp ( Figure 2I). Caudal to Cant, the expression gets weaker in Dm, Dc and Dp (data not shown). In summary, *emx1* and *emx2* show a very restricted expression pattern in the adult zebrafish pallium ( Figure 2D–F, Dataset 5; Table 1). Expression of *emx3* is present in Dm, Dc and Dlv/Dp ( Figure 2J–L, Dataset 5; Table 1).

### Expression of Prox1 in the adult zebrafish pallium

**Figure 3.**
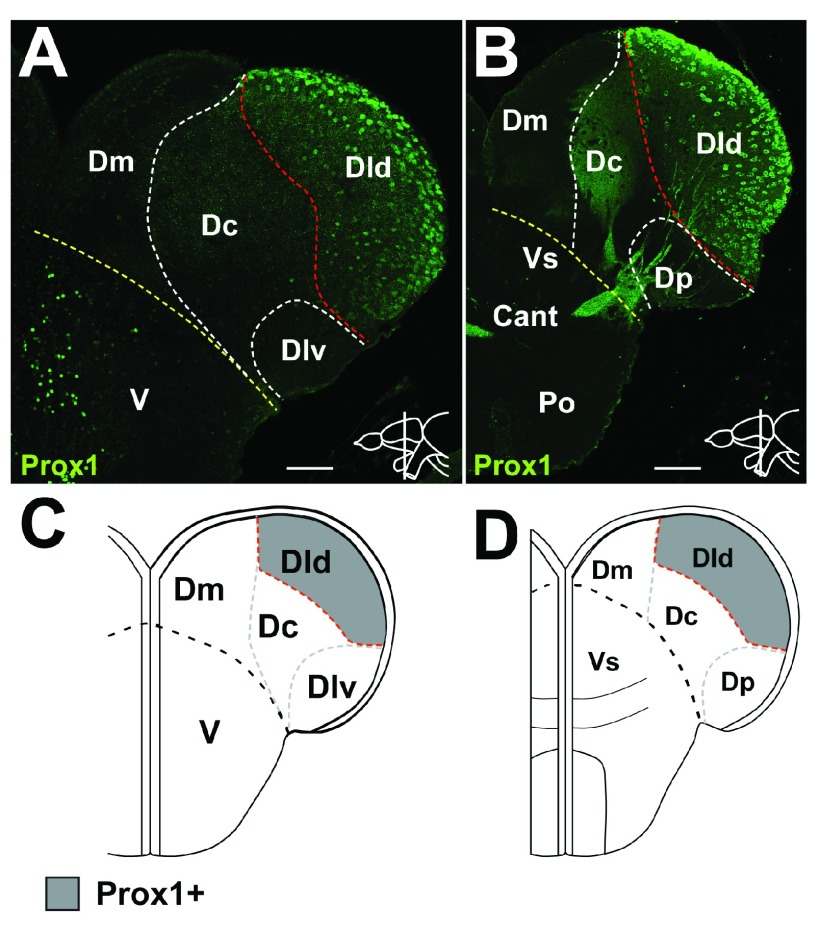
Expression of Prox1 in the pallium. **A**. At mid-telencephalic levels, Prox1 positive cells are found in the neuronal layer (nl) of Dld shortly before the anterior commissure (Cant). **B**. At Cant, Prox1 positive cells are found in the nl of Dld. **C**.– **D**. Summary of expression pattern of Prox1. **A**.– **B**. Confocal images of cross-sections at the levels indicated through the telencephalon. Red dashed line indicates subdivisions based on the current marker. White dashed line indicates subdivisions based on other markers. The yellow dashed line indicates the boundary between D and V. Scale bars = 50µm in **A**– **B**.

During mouse development, *Prox1* expression is found in the amygdala, dentate gyrus and in the neocortex ^57^. At adult stages, however, strong Prox1 expression is restricted to the dentate gyrus of the hippocampus and is commonly used as a specific marker for granule cells of the hippocampus ^57– 60^. In the adult zebrafish pallium, Prox1+ cells only are present in the midtelencephalon shortly rostral to Cant in the neuronal layer of Dld ( Figure 3A). This expression pattern continues to the anterior commissure ( Figure 3B). Shortly caudal to Cant, only scattered Prox1+ cells are present in Dld, more caudally no Prox1+ cells can be found (data not shown). In summary, Prox1 staining is present in Dld starting at mid-telencephalic levels until shortly caudal to Cant ( Figure 3A–D, Dataset 6; Table 1).

### Expression of *ascl1a* in the adult zebrafish pallium

**Figure 4.**
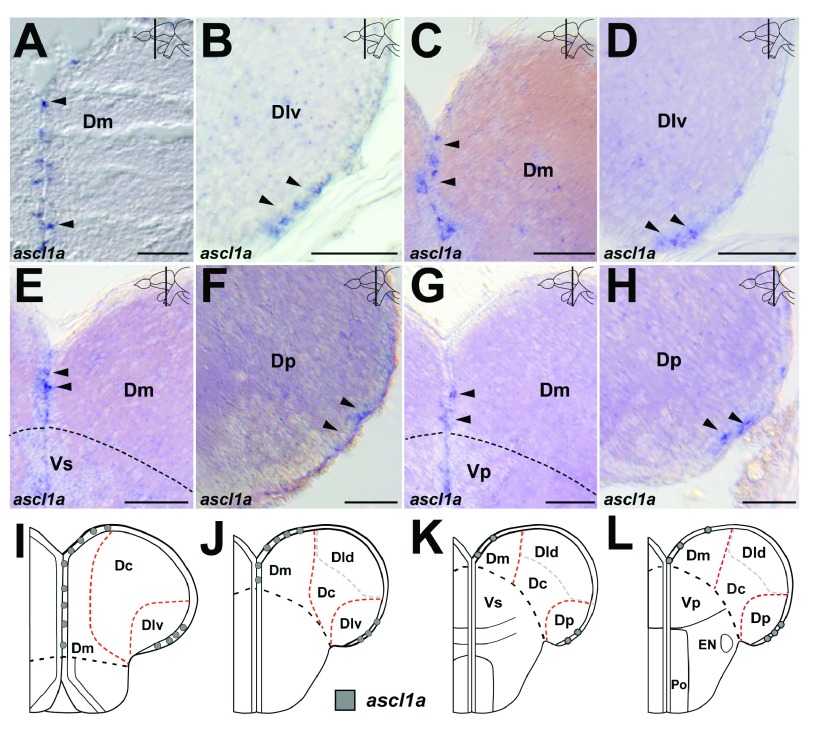
Expression of *ascl1a* in the pallium. **A**.– **B**. In the rostral telencephalon, *ascl1a* expression is present in scattered cells in the ventricular zone (vz) of Dm ( **A**, arrowheads) and Dlv ( **B**, arrowheads). **C**.– **D**. At mid-telencephalic levels, *ascl1a* expression is present in scattered cells in the vz of Dm ( **C**, arrowheads) and Dlv ( **D**, arrowheads). **E**.– **F**. At Cant, *ascl1a* expression is present in scattered cells in the vz of Dm ( **E**, arrowheads) and Dp ( **F**, arrowheads). **G**.– **H**. Posterior to Cant, *ascl1a* expression is present in scattered cells in the vz of Dm ( **G**, arrowheads) and Dp ( **H**, arrowheads). **I**.– **L**. Summary of the expression pattern of *ascl1a* rostral ( **I**.), mid-telencephalic ( **J**.), commissural ( **K**.) and postcommissural levels ( **L**.). **A**.- **H**. Brightfield images of cross-sections at the levels indicated through the telencephalon. Red dashed line indicates subdivisions based on the current marker. White dashed line indicates subdivisions based on other markers. The black dashed line indicates the boundary between D and V. Scale bars = 50µm in **A**– **C**.

In tetrapod embryos, *Ascl1* is expressed a subpopulation of progenitors in the dorsal telencephalon ^61, 62^. In the zebrafish embryo, *ascl1a* expression is present in the caudomedial ventricular zone of the dorsal telencephalon ^63^. In the adult zebrafish pallium, *ascl1a* is expressed in scattered cells in the ventricular zone of Dm and in scattered cells in the ventricular zone of Dlv ( Figure 4A–D, arrowheads, Figure 4I,J, Dataset 7; Table 1). At Cant and posterior to Cant *ascl1a* is present in scattered cells in Dm and Dp ( Figure 4E–H, arrowheads, Figure 4K,L, Dataset 7; Table 1).

Gene expression analysis in the adult zebrafish pallium Dataset 1 Expression of eomesb in the embryonic brain and the adult pallium in zebrafish. Raw data of Figure S2 and additional image files of eomesb expression in the embryo and the adult pallium.
Dataset 2 Images of negative control. No signal was detected in the absence of the riboprobe, demonstrating that the antibody reacts specifically with the synthetic RNA.
Dataset 3 Expression of eomesa in the zebrafish pallium. Raw data of Figure 1 and additional image files of eomesa expression in the adult pallium.
Dataset 4 Expression of emx1, emx2 and emx3 in the zebrafish larval brain. Raw data of Figure S3 and additional image files of emx gene expression in the zebrafish larvae.
Dataset 5 Expression of emx1, emx2 and emx3 in the zebrafish pallium. Raw data of Figure 2 and additional image files of emx gene expression in the adult pallium.
Dataset 6 Expression of Prox1 in the zebrafish pallium. Raw data of Figure 3 and additional image files of Prox1 expression in the adult pallium.
Dataset 7 Expression of ascl1a in the zebrafish pallium. Raw data of Figure 4 and additional image files of ascl1a expression in the adult pallium. Click here for additional data file. Copyright: © 2015 Ganz J et al. 2015 Data associated with the article are available under the terms of the Creative Commons Zero "No rights reserved" data waiver (CC0 1.0 Public domain dedication).

## Discussion

### Subdivisions of the zebrafish pallium and their homology to other vertebrates

**Figure 5.**
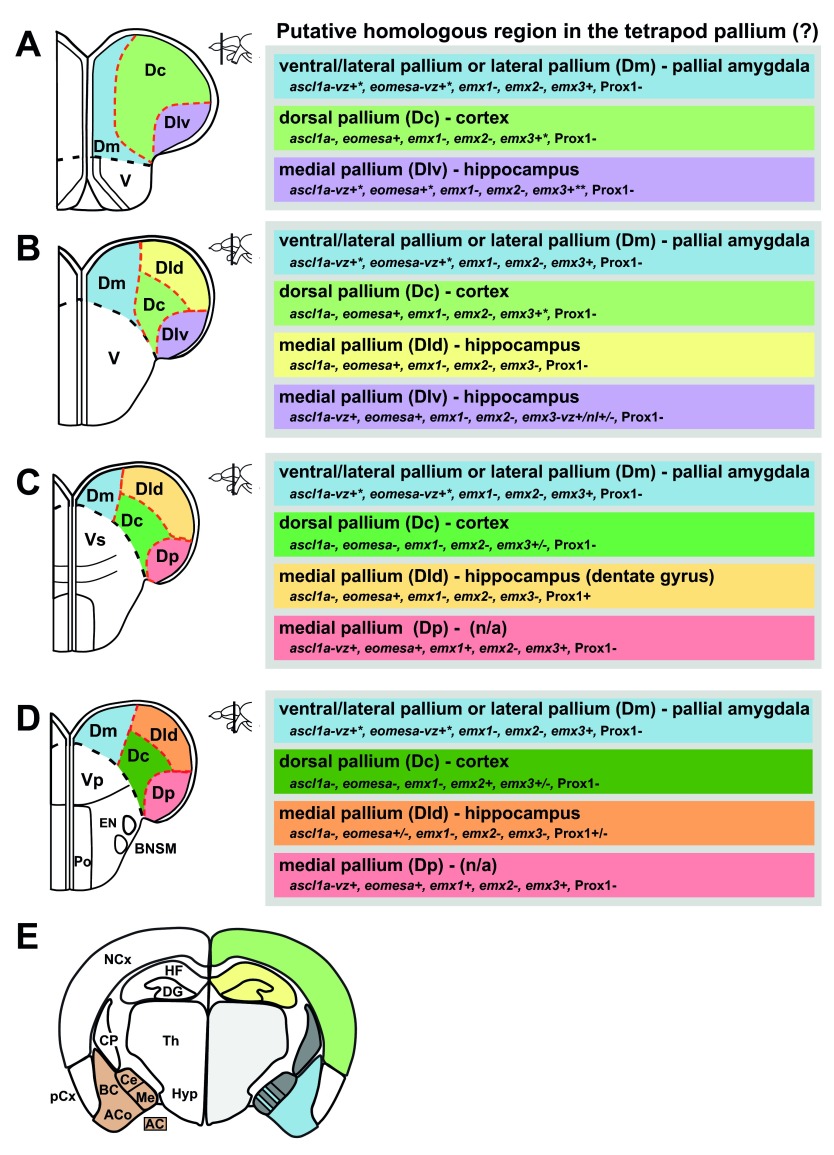
Summary of the gene expression patterns in the adult zebrafish pallium. **A**.– **D**. Cross-sections at the levels indicated through the telencephalon. **E**. Schematic diagram of a cross section through the mouse telencephalon for comparison (modified after ^14^), note that the amygdaloid complex (AC, brown) is derived both from subpallium (grey) and pallium. Light grey areas (Th/Hyp) are part of the diencephalon. Indicated is also a model of the putative homology of the subdivisions in the adult zebrafish pallium to regions in the tetrapod pallium taking the data presented in this paper into account. Additional marker analysis, lineage tracing experiments and functional analyses are necessary to substantiate the proposed homology to pallial nuclei in tetrapods. The different shades of green (Dc) and yellow (Dld) indicate gene expression changes along the rostro-caudal axis. * in scattered cells, ** in Dlv in the ventricular zone, moving caudally expression in the neuronal layer and ventricular zone, note that Prox1 positive cells are present in Dld shortly before the anterior commissure, n/a = not applicable. The black dashed line indicates the boundary between D and V. The red dashed lines indicate the boundaries between different nuclei in **D**. AC amygdaloid complex, ACo anterior cortical amygdalar area, BC basal amygdalar complex, BNSM bed nucleus of the stria medullaris, Ce central amygdala, CP Caudateputamen, DG dentate gyrus, EN entopeduncular nucleus, HF hippocampal formation, Hyp hypothalamus, Me medial amygdala, NCx neocortex, pCx piriform cortex, Po preoptic region, Th thalamus, V area ventralis telencephali, Vp postcommissural nucleus of the area ventralis telencephali, Vs supracommisural nucleus of the area ventralis telencephali.

Due to the different development, the pallium of ray-finned fishes has a markedly different morphology compared to all other vertebrates, which makes the comparison between the areas of the pallium of ray-finned fishes to pallial nuclei of other vertebrates particularly challenging. Yet, the correct assignment of homologous pallial areas between teleosts and tetrapods is essential for usage of the teleost fish model in neurobiological research. We have analyzed several conserved molecular marker genes that are found in specific areas of the pallium in the domestic mouse, chicken and the African clawed frog. Based on the expression analysis we identify four main subdivisions of the pallium (Dm, Dl, Dc and Dp) and propose that Dl is subdivided in a dorsal (Dld) and ventral part (Dlv). Based on our data we also suggest putative homologies to pallial nuclei in tetrapods. We suggest that Dm is homologous to the ventral or ventral/lateral pallium, Dc to the dorsal pallium, Dl to the medial pallium, and suggest that Dp comprises a specialized part of Dl ( Figure 5). Additional marker analysis, lineage tracing experiments and functional analyses will be necessary to substantiate the proposed pallial subdivisions and their homology to pallial nuclei in tetrapods. As our study is based solely on comparative gene expression data, we have discussed our results in the framework of gene expression data of other organisms and subsequently compared them to connectional, neurochemical, and functional data in teleosts. For clarity, we discuss our results separately for each pallial subdivision.

### Medial part of the area dorsalis telencephali (Dm)

In the African clawed frog, the chicken, and the domestic mouse, four pallial divisions have been identified in the embryo, the ventral pallium (VP), the lateral pallium (LP), the dorsal pallium (DP) and the medial pallium (MP) ^9, 10, 13, 21^. In the African clawed frog, chicken, and domestic mouse, the ventral pallial subdivision is characterized in the embryo by the absence of *Emx1* expression and presence of the pallial marker *Tbr1* ^9, 10, 13, 21^. In embryonic and adult zebrafish, *tbr1* is expressed throughout the pallium ^7, 53, 54^. In adult ray-finned fishes, Dm has been proposed to be homologous to the ventral pallium (pallial amygdala) based on topological, connectional and functional data ^29– 31, 64– 66^. In contrast, Nieuwenhuys (2009) proposed that Dm is homologous to the lateral pallium based on topology and Yamamoto *et al.* (2007) suggest that Dm together with Dd and Dld is homologous to the dorsal pallium. Thus, we analyzed the expression of the *emx* genes in the larval and adult zebrafish to determine if the absence of these markers identifies a ventral pallial subdivision in the zebrafish pallium. In zebrafish, the *emx* genes show a dynamic expression pattern both in the embryo and the adult. At 1dpf, the three *emx* genes are expressed at throughout the dorsal telencephalon ^49, 55, 56^. We found that expression of *emx1* is restricted to a caudolateral expression domain in the zebrafish larvae at 7dpf and to Dp in the adult ( Figure 2D,E, Figure S3). Similarly, *emx2* also shows a restricted expression pattern to a caudolateral domain both in the zebrafish larvae at 7dpf and in the adult zebrafish pallium ( Figure 2F, Figure S3). The expression of *emx3* is present in the entire pallium in the zebrafish embryo and larvae ( ^49^, Figure S3C). In the adult, *emx3* is present in the rostral telencephalon in Dm, Dc and in the ventricular zone of Dlv ( Figure 2J). Moving caudally, *emx3* is present in Dm, in scattered cells in Dc and in the ventricular zone and neuronal layer of Dlv and Dp ( Figure 2K,L). As it has been previously suggested that zebrafish *emx3* supplies the functions provided by *Emx1* and *Emx2* in mouse ^49^, we took the combined expression pattern of the three *emx* genes into account for our analysis. The lack of an area in Dm that is *tbr1* positive and *emx* gene negative suggests that zebrafish might not have a distinct ventral pallial subdivision and that Dm is either homologous to the lateral pallium, or that Dm comprises a combined ventral and lateral pallium ( Figure 5A–D). *Emx1* alone might not be an adequate marker for the ventral pallium in the domestic mouse and chicken, as they have lost the *Emx3* gene ^49^.

Both the ventral pallium and the lateral pallium contribute together with subpallial derivatives to the amygdaloid complex in tetrapods ^10, 67, 68^. In rodents, the Cannabinoid Receptor (CB1) is expressed in a subset of neurons in the basolateral amygdala ^69, 70^. In the weakly electric fish *Apteronotus leptorhynchus* and zebrafish, *cb1* is expressed in Dm ^71– 73^, thus supporting the model that Dm is homologous to the pallial amygdala. Based on gene expression analysis, we have suggested previously that the supracommissural nucleus of the area ventralis telencephali (Vs) is homologous to the dorsal and ventral part of the central amygdala and the bed nucleus of the stria terminalis (BST) and that the postcommissural nucleus of the area ventralis telencephali (Vp) is homologous to the dorsal part of the central amygdala and the BST ^7^. Thus, it is plausible that Vs and Vp (subpallial amygdala) and Dm (pallial amygdala) form the amygdaloid complex in the adult zebrafish ( Figure 5A–E).

### Lateral part of the area dorsalis telencephali (Dl)

Based on topological and gene expression data it has been proposed that the entire Dl ^4^ or Dl excluding Dlv or Dp is homologous to the medial pallium in other vertebrates ^24, 30, 32, 64^. In contrast, Wullimann and Mueller (2004) considered only Dlv to be homologous to the medial pallium and Yamamoto *et al.* (2007) proposed that only the dorsal part of Dl is homologous to the medial pallium. Functional ablation experiments suggested that Dl is equivalent to the hippocampus of tetrapods ^65^. Our combinatorial expression data suggests that Dl is subdivided in Dld and Dlv rostrally and at Cant and posterior to Cant in Dld and Dp ( Figure 5A–D). Dld is different from Dc based on absence of *emx3* positive cells in Dld. During mouse development, *Prox1* expression is found in the amygdala, dentate gyrus and in the neocortex ^57^. At adult stages, however, strong *Prox1* expression is restricted to the dentate gyrus of the hippocampus and is commonly used as a specific marker for granule cells of the hippocampus ^57– 59^. In the adult zebrafish, Prox1 positive cells are exclusively present in the caudal part of Dld. In summary, our data suggests that Dl (excluding Dp, see below) may be homologous to the medial pallium and hippocampus and the caudal part of Dld homologous to the dentate gyrus in the domestic mouse ( Figure 5A–E). We will discuss the possible homology of Dp in the subsequent part.

### Posterior part of D (Dp)

It has previously been shown that Dp receives olfactory input and has been on this basis homologized to the lateral pallium in amphibians and all other gnathostomes with evaginated forebrains ^4^. Being homologous to the lateral pallium, it should be located next to Dm in the embryo, the presumptive ventral pallium. The discrepancy between the position of Dp in the adult and an expected location next to Dm has led to different models to explain the different position of Dp in the adult pallium: “the partial pallial eversion model” by Wullimann and Mueller ^31, 32, 52, 74^, the “eversion-rearrangement theory” by Northcutt and Braford ^33, 64, 75^, and the “new eversion model” by Yamamoto and colleagues ^37^. In the “partial pallial eversion model” ^31, 32, 52, 74^, based on connectional and gene expression data, it has been proposed that the homolog of the lateral pallium does not participate in the eversion. Wullimann and Mueller put forward that neuroblasts generated by the ventricular zone of the uneverted, medially located lateral pallium homolog migrate laterally to give rise to the submeningeally located nucleus Dp ^31, 74^. Our gene expression analysis does not support this part of their modified partial eversion model. Based on differential gene expression we can identify Dp, which is not located submeningeally, but has its own ventricular zone even in the adult ^4, 64, 76^. New neurons are still generated in the zebrafish pallium in the adult ^77, 78^. Thus, a small area of ventricular zone should be still present next to Dm to generate neurons for Dp. However, we have not observed neuroblasts migrating laterally across the pallium to reach Dp in the adult by BrdU pulse chase studies or genetic lineage tracing ^51, 76, 77^. The “eversion-rearrangement theory” by Northcutt and Braford suggests that differential expansion of the ventricular surface of some pallial zones and differential proliferation and migration of neuroblasts from the different ventricular zones might result in displacement or shifting of the different pallial subdivisions ^33, 64, 75^. Similar to the “partial pallial eversion model”, they propose that a small stretch of Dp is still located between Dm and Dl. Our gene expression data does not support this aspect of both models, as we do not identify a small area of ventricular zone and neuronal layer sandwiched between Dm and Dc rostrally and Dm and Dld more caudally that matches the gene expression profile found in Dp.

In the “new eversion model”, the eversion was suggested to occur in a caudolateral direction leading to a shift of the arrangement of the different pallial subdivisions ^37^. Yamamoto and colleagues propose that ventral pallial and lateral pallial homologs are not present in the rostral but only in the caudal pallium. In their model they suggest that Dp is homologous to the lateral pallium ^37^. In contrast, we find the putative ventral or ventral/lateral pallial homolog Dm contiguously from rostral to caudal. However, we also identify a separate Dp nucleus only in the posterior part of the pallium.

In contrast to the models discussed above, Nieuwenhuys has put forward that the lateral olfactory tract in vertebrates with everted telencephala is not homologous to the olfactory tract in vertebrates with evaginated telencephala ^4^. He bases this on data that showed considerable variation in the pattern of secondary olfactory projections among different groups of ray-finned fishes and showed secondary olfactory projections both to Dm and Dp ^4, 29, 75^. He suggests that the olfactory input has increased overall in Dl, with a subsequent confinement to Dp and has decreased in Dm during ray-fin fish evolution. He considers the lateral olfactory tract as an apomorphy of actinopterygian pallia ^4^. Even though our gene expression data does not support the models that suggest a migration or displacement of Dp from a position equivalent to the lateral pallium to its caudolateral position, we can identify Dp as a subdivision of Dl separate from Dld. Furthermore, Neuropeptide Y and Parvalbumin immunohistochemistry clearly distinguishes Dl from Dp ^79^. Furthermore, as discussed above, our gene expression data suggest that Dm might be homologous to the lateral pallium or comprise a combined ventral and lateral pallium, which might suggest that Dp is not homologous to the lateral pallium. Our gene expression data is consistent with Nieuwenhuys’ model that Dp comprises a specialized part of Dl that receives olfactory input, even though the other parts of Dl might be homologous to the medial pallium and hippocampus in tetrapods ( Figure 5A–D).

### Central part of the area dorsalis telencephali (Dc)

In the “modified partial pallial eversion model” ^32^, based on expression of Parvalbumin and nicotine adenine dinucleotide phosphate diphorase (NADPHd) in Dl, it was suggested that Dc is a true histogenetic unit that has its own germinative zone rostrally, but is caudally displaced to a more central location by differential growth of Dm and Dl. Mueller and colleagues suggest that Dc is homologous to the dorsal pallium in other vertebrates and that Dd is absent in zebrafish ^32^. This model is consistent with connectional and gene expression data in *Apteronotus leptorhynchus* ^24^. In addition, it has been noted that Dc shows no significant immunoreactivity to Calretinin, Neuropeptide Y or Thyrosine hydroxylase separating it from the surrounding areas of the pallium ^79^. Other models based mainly on topology suggest that Dm, Dd and Dld are homologous to the dorsal pallium ^37^, whereas others have homologized Dd with the dorsal pallium ^4, 29, 30, 33, 64^ and have suggested that Dc is not a separate unit, but represents the deeper zone of the periventricular areas of Dm, Dd and Dl. Our gene expression data is consistent with the “modified partial pallial eversion model” of Mueller and colleagues with regard to Dc. We have adapted the nomenclature of Mueller *et al.* (2011) to call the displaced nucleus Dc, even though our data do not rule out the possibility that Dc is rather a displaced Dd nucleus. Accordingly, Dc does not have its own germinative zone caudally, but only in the rostral part of the pallium. However, the morphogenesis of the telencephalon has been analyzed between 1dpf and 5dpf and no such displacement of Dc has been described ^5^, so if it is the case, then it has to happen after 5dpf. To conclusively evaluate the displacement of Dc, detailed lineage analysis using Cre/loxP technology should be performed from embryonic stages till adulthood.

### Gene expression in pallial progenitors

In the adult zebrafish, proliferating cells are found scattered in the ventricular zone of the pallium ^76– 78^. In the adult zebrafish pallium, *ascl1a* is found in scattered cells in the ventricular zone of Dm and in Dlv/Dp. In addition, *eomesa* is present in scattered cells in the ventricular zone of Dm and in the ventricular zone of Dld and Dlv/Dp. *Emx1* is also present in the ventricular zone of Dp and *emx3* is found in the ventricular zone of Dm, Dlv and Dp ( Figure 5A–D).

In mouse, *Ascl1* is expressed in a subpopulation of progenitors in the dorsal telencephalon ^61, 62^. *Eomes (Tbr2)* is present in intermediate progenitors in the cortex in the embryo and in the dentate gyrus both in the embryo and the adult and is part of the glutamatergic differentiation cascade that also contains *NeuroD* and *Tbr1* ^80^. In the adult zebrafish, *neurod* and *tbr1* as well as the vesicular glutamate transporters *vglut1/2.1/2.2* marking glutamatergic neurons are expressed throughout the pallium, suggesting that the glutamatergic differentiation cascade may still be present in the adult zebrafish telencephalon ^7, 72^. The expression in scattered cells in the ventricular zone suggests that *eomesa* and *ascl1a* may label progenitor subpopulations in the pallium and *eomesa* could play a role in differentiation of glutamatergic neurons in the adult zebrafish brain. In addition, *emx1* and *emx3* might be involved in regulating neuronal differentiation processes in the pallium. It will be interesting to perform comprehensive lineage analysis using Cre/loxP technology to follow the progeny of these different markers in the adult zebrafish.

## Conclusions

In this study, we present a new model of the subdivisions in the adult zebrafish pallium based on conserved marker gene expression and propose putative homologies to pallial nuclei in tetrapods. Additional marker analysis, lineage tracing experiments and functional analyses are necessary to substantiate the proposed pallial subdivisions and their homology to pallial nuclei in tetrapods. It is important to identify pallial areas in adult zebrafish and their homologies to pallial nuclei in tetrapods to improve our knowledge about the zebrafish brain, in order to implement the zebrafish system as an ideal model for neurobiological research and as a model for human neurodegenerative diseases.

## List of abbreviations

BNSM: bed nucleus of the stria medullaris
BST: bed nucleus of the stria terminalis
Cant: anterior commissure
D: area dorsalis telencephali
Dc: central part of the area dorsalis telencephali
Dd: dorsal part of the area dorsalis telencephali
Dl: lateral part of the area dorsalis telencephali
Dld: dorsal part of Dl
Dlv: ventral part of Dl
Dm: medial part of the area dorsalis telencephali
Dp: posterior part of the area dorsalis telencephali
dpf: days post fertilization
EN: entopeduncular nucleus
nl: neuronal layer
OB: olfactory bulb
Po: preoptic region
V: area ventralis telencephali
Vp: postcommissural nucleus of the area ventralis telencephali
Vs: supracommisural nucleus of the area ventralis telencephali
vz: ventricular zone

## Data availability

The data referenced by this article are under copyright with the following copyright statement: Copyright: © 2015 Ganz J et al.

Data associated with the article are available under the terms of the Creative Commons Zero "No rights reserved" data waiver (CC0 1.0 Public domain dedication). 

*Figshare:* Gene expression analysis in the adult zebrafish pallium. Doi: http://dx.doi.org/10.6084/m9.figshare.1266194 ^81^
