# Supplementary figures and images for: Subdivisions of the adult zebrafish pallium based on molecular marker analysis
Source: F1000Res. 2015 Nov 4;3:308. Originally published 2014 Dec 17. [Version 2] doi: 10.12688/f1000research.5595.2 (PMC4335597; doi:10.12688/f1000research.5595.2)

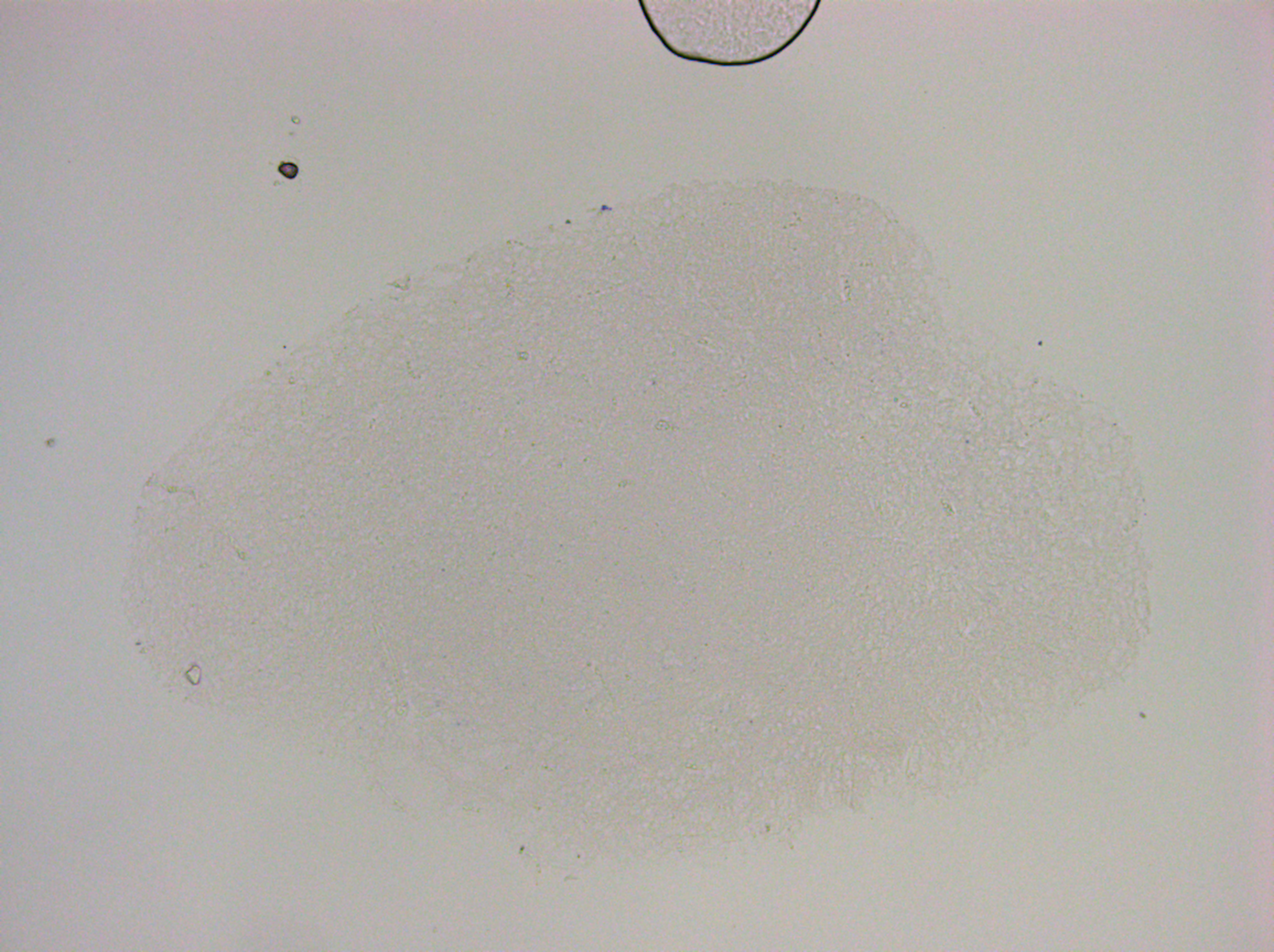

Supplement: Gene expression analysis in the adult zebrafish pallium — Dataset 1 Expression of eomesb in the embryonic brain and the adult pallium in zebrafish. Raw data of Figure S2 and additional image files of eomesb expression in the embryo and the adult pallium. Dataset 2 Images of negative control. No signal was detected in the absence of the riboprobe, demonstrating that the antibody reacts specifically with the synthetic RNA. Dataset 3 Expression of eomesa in the zebrafish pallium. Raw data of Figure 1 and additional image files of eomesa expression in the adult pallium. Dataset 4 Expression of emx1, emx2 and emx3 in the zebrafish larval brain. Raw data of Figure S3 and additional image files of emx gene expression in the zebrafish larvae. Dataset 5 Expression of emx1, emx2 and emx3 in the zebrafish pallium. Raw data of Figure 2 and additional image files of emx gene expression in the adult pallium. Dataset 6 Expression of Prox1 in the zebrafish pallium. Raw data of Figure 3 and additional image files of Prox1 expression in the adult pallium. Dataset 7 Expression of ascl1a in the zebrafish pallium. Raw data of Figure 4 and additional image files of ascl1a expression in the adult pallium. [file f1000research-3-7777-s0000.tgz › eomesb_adult_1.tif]

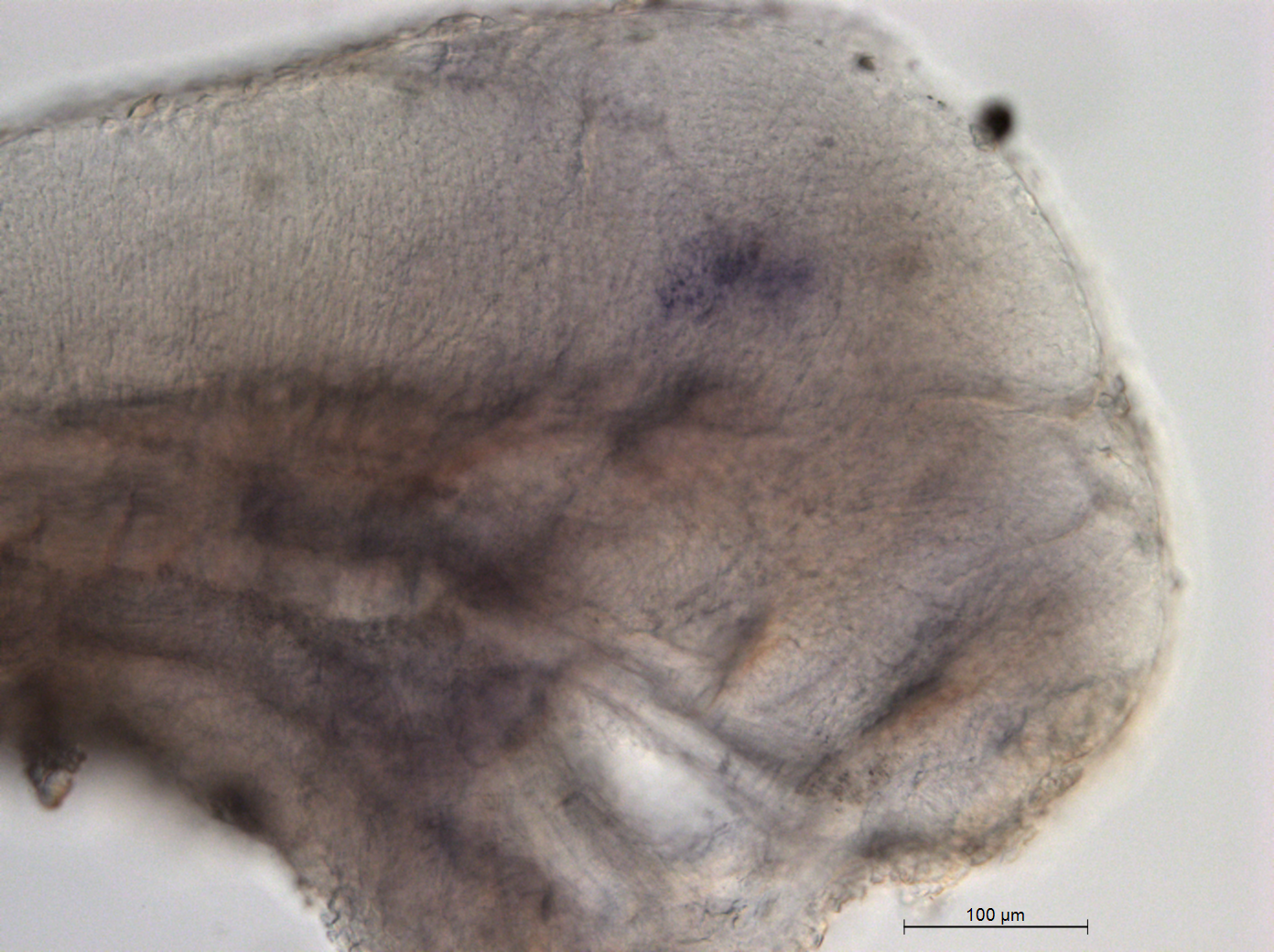

Supplement: Gene expression analysis in the adult zebrafish pallium — Dataset 1 Expression of eomesb in the embryonic brain and the adult pallium in zebrafish. Raw data of Figure S2 and additional image files of eomesb expression in the embryo and the adult pallium. Dataset 2 Images of negative control. No signal was detected in the absence of the riboprobe, demonstrating that the antibody reacts specifically with the synthetic RNA. Dataset 3 Expression of eomesa in the zebrafish pallium. Raw data of Figure 1 and additional image files of eomesa expression in the adult pallium. Dataset 4 Expression of emx1, emx2 and emx3 in the zebrafish larval brain. Raw data of Figure S3 and additional image files of emx gene expression in the zebrafish larvae. Dataset 5 Expression of emx1, emx2 and emx3 in the zebrafish pallium. Raw data of Figure 2 and additional image files of emx gene expression in the adult pallium. Dataset 6 Expression of Prox1 in the zebrafish pallium. Raw data of Figure 3 and additional image files of Prox1 expression in the adult pallium. Dataset 7 Expression of ascl1a in the zebrafish pallium. Raw data of Figure 4 and additional image files of ascl1a expression in the adult pallium. [file f1000research-3-7777-s0000.tgz › eomesb_embryo.tif]

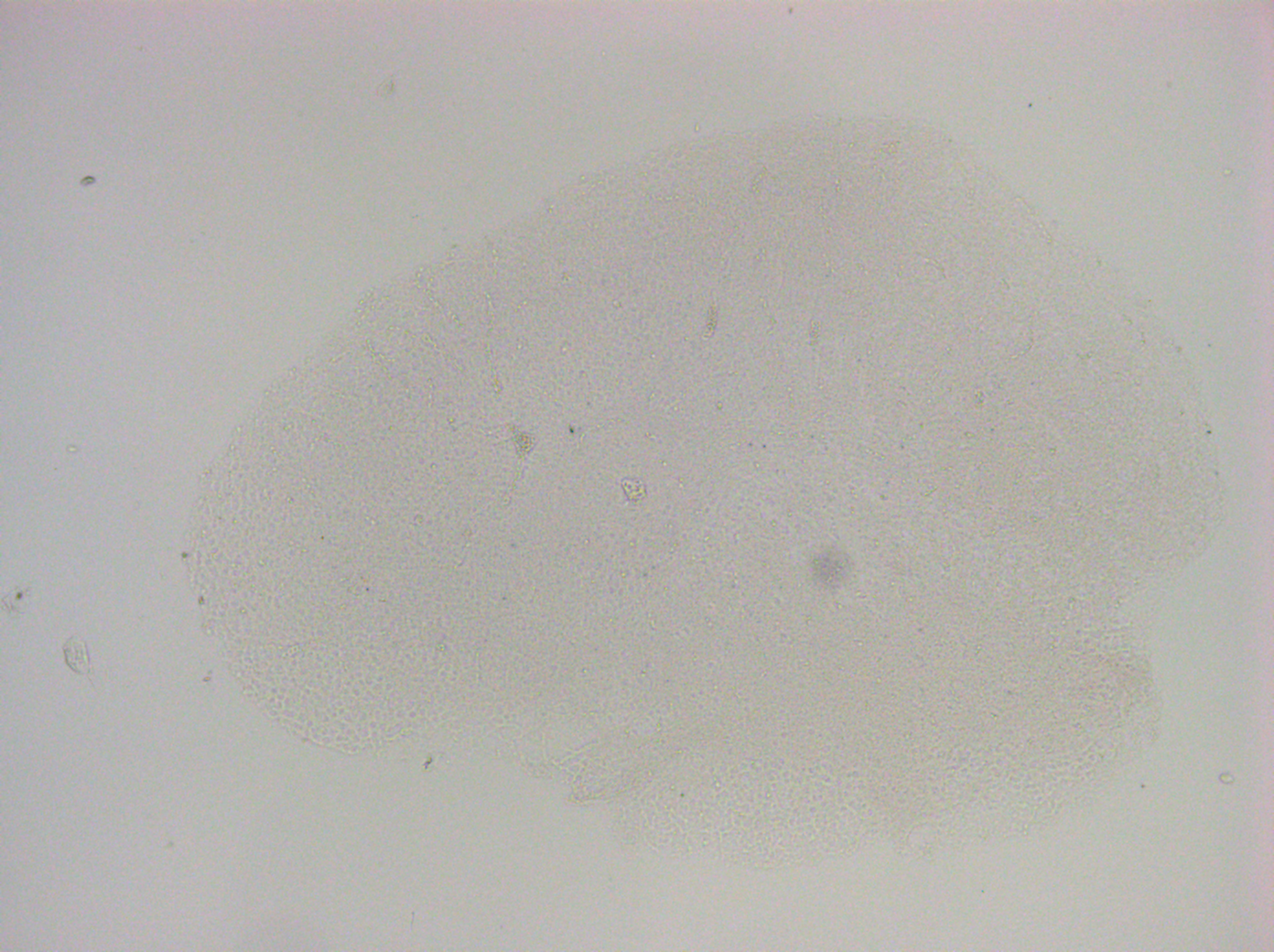

Supplement: Gene expression analysis in the adult zebrafish pallium — Dataset 1 Expression of eomesb in the embryonic brain and the adult pallium in zebrafish. Raw data of Figure S2 and additional image files of eomesb expression in the embryo and the adult pallium. Dataset 2 Images of negative control. No signal was detected in the absence of the riboprobe, demonstrating that the antibody reacts specifically with the synthetic RNA. Dataset 3 Expression of eomesa in the zebrafish pallium. Raw data of Figure 1 and additional image files of eomesa expression in the adult pallium. Dataset 4 Expression of emx1, emx2 and emx3 in the zebrafish larval brain. Raw data of Figure S3 and additional image files of emx gene expression in the zebrafish larvae. Dataset 5 Expression of emx1, emx2 and emx3 in the zebrafish pallium. Raw data of Figure 2 and additional image files of emx gene expression in the adult pallium. Dataset 6 Expression of Prox1 in the zebrafish pallium. Raw data of Figure 3 and additional image files of Prox1 expression in the adult pallium. Dataset 7 Expression of ascl1a in the zebrafish pallium. Raw data of Figure 4 and additional image files of ascl1a expression in the adult pallium. [file f1000research-3-7777-s0000.tgz › eomesb_adult_2.tif]

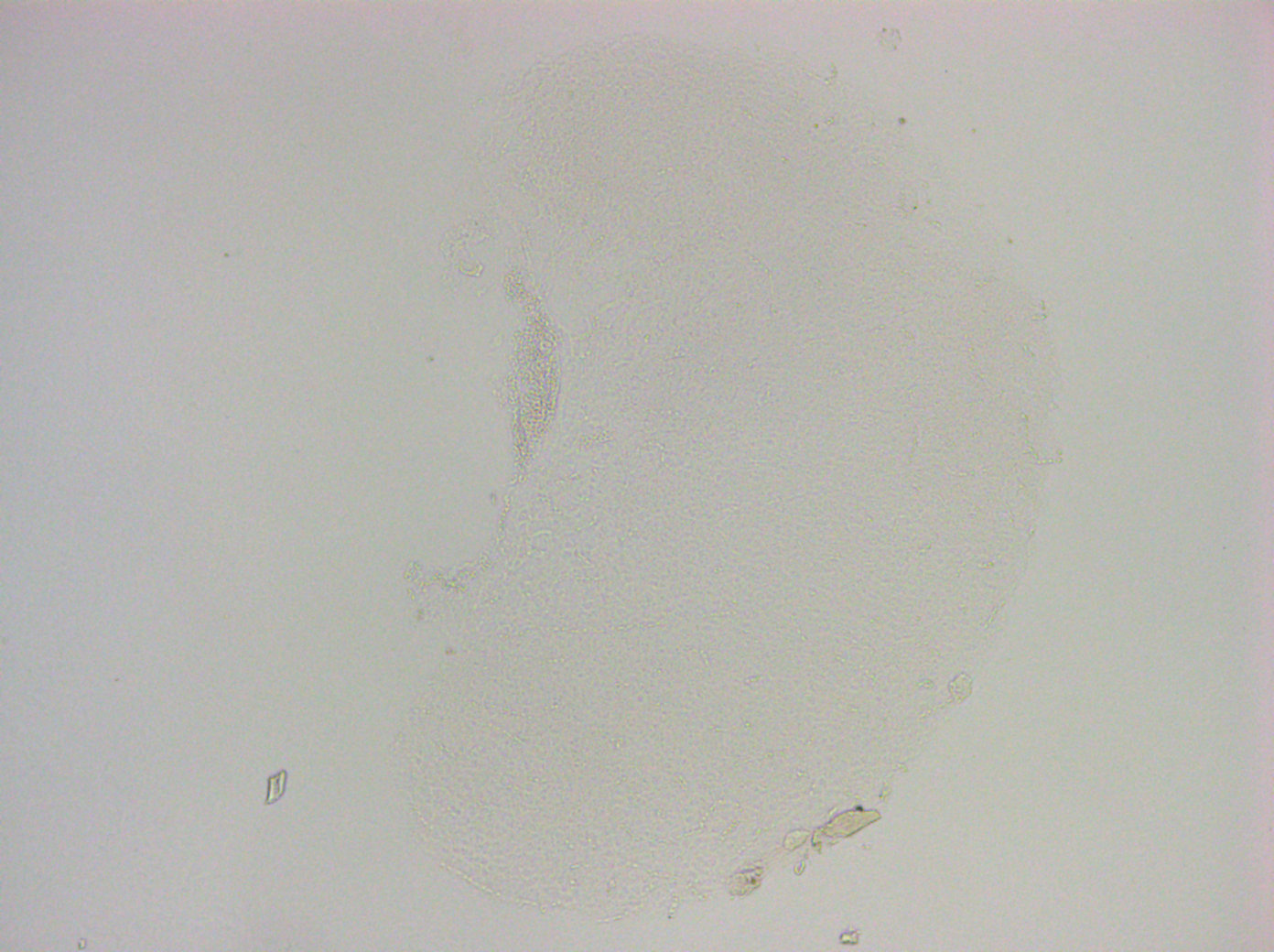

Supplement: Gene expression analysis in the adult zebrafish pallium — Dataset 1 Expression of eomesb in the embryonic brain and the adult pallium in zebrafish. Raw data of Figure S2 and additional image files of eomesb expression in the embryo and the adult pallium. Dataset 2 Images of negative control. No signal was detected in the absence of the riboprobe, demonstrating that the antibody reacts specifically with the synthetic RNA. Dataset 3 Expression of eomesa in the zebrafish pallium. Raw data of Figure 1 and additional image files of eomesa expression in the adult pallium. Dataset 4 Expression of emx1, emx2 and emx3 in the zebrafish larval brain. Raw data of Figure S3 and additional image files of emx gene expression in the zebrafish larvae. Dataset 5 Expression of emx1, emx2 and emx3 in the zebrafish pallium. Raw data of Figure 2 and additional image files of emx gene expression in the adult pallium. Dataset 6 Expression of Prox1 in the zebrafish pallium. Raw data of Figure 3 and additional image files of Prox1 expression in the adult pallium. Dataset 7 Expression of ascl1a in the zebrafish pallium. Raw data of Figure 4 and additional image files of ascl1a expression in the adult pallium. [file f1000research-3-7777-s0000.tgz › NegativeControl_1.tif]

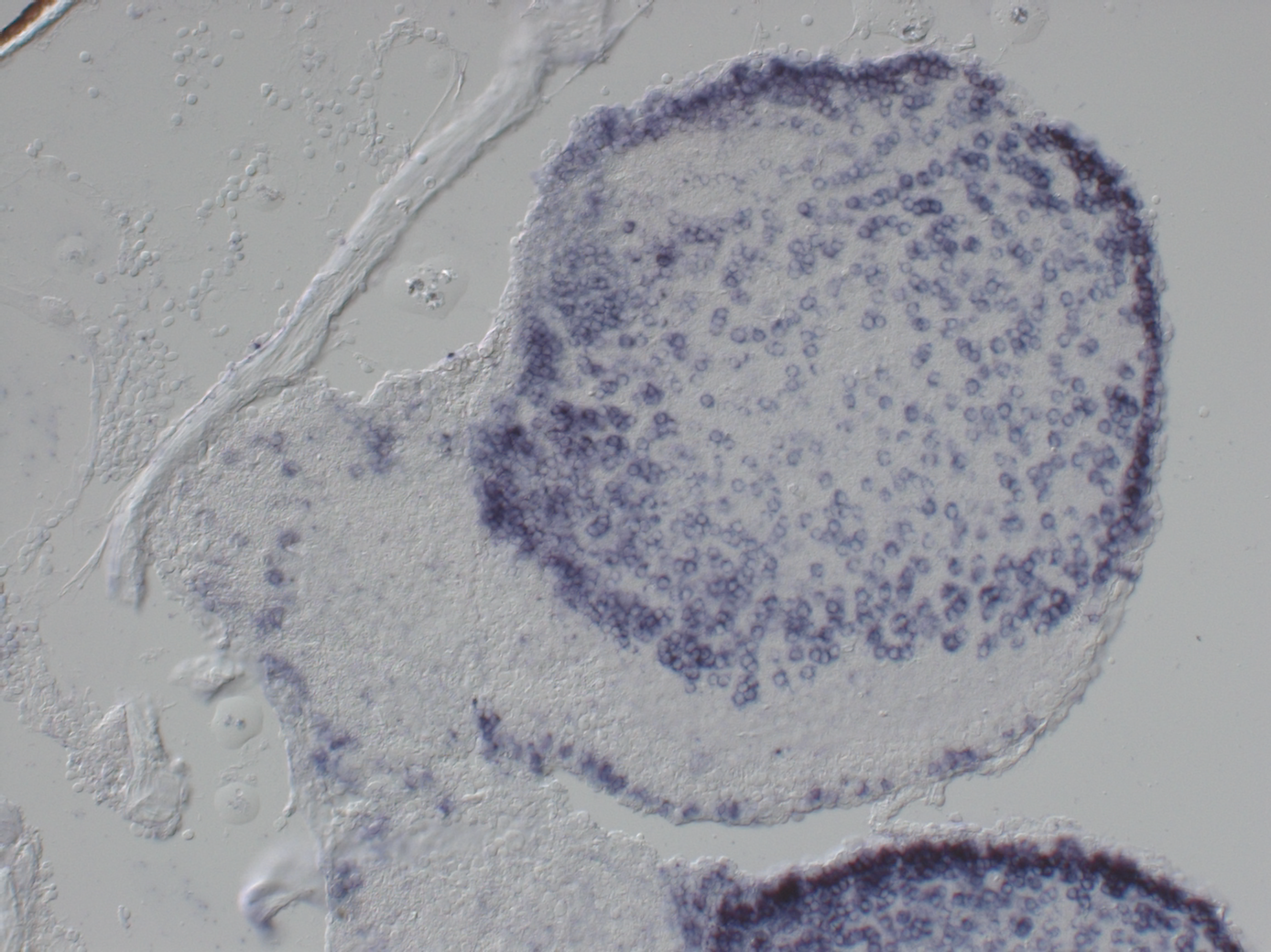

Supplement: Gene expression analysis in the adult zebrafish pallium — Dataset 1 Expression of eomesb in the embryonic brain and the adult pallium in zebrafish. Raw data of Figure S2 and additional image files of eomesb expression in the embryo and the adult pallium. Dataset 2 Images of negative control. No signal was detected in the absence of the riboprobe, demonstrating that the antibody reacts specifically with the synthetic RNA. Dataset 3 Expression of eomesa in the zebrafish pallium. Raw data of Figure 1 and additional image files of eomesa expression in the adult pallium. Dataset 4 Expression of emx1, emx2 and emx3 in the zebrafish larval brain. Raw data of Figure S3 and additional image files of emx gene expression in the zebrafish larvae. Dataset 5 Expression of emx1, emx2 and emx3 in the zebrafish pallium. Raw data of Figure 2 and additional image files of emx gene expression in the adult pallium. Dataset 6 Expression of Prox1 in the zebrafish pallium. Raw data of Figure 3 and additional image files of Prox1 expression in the adult pallium. Dataset 7 Expression of ascl1a in the zebrafish pallium. Raw data of Figure 4 and additional image files of ascl1a expression in the adult pallium. [file f1000research-3-7777-s0000.tgz › eomesa_1.TIF]

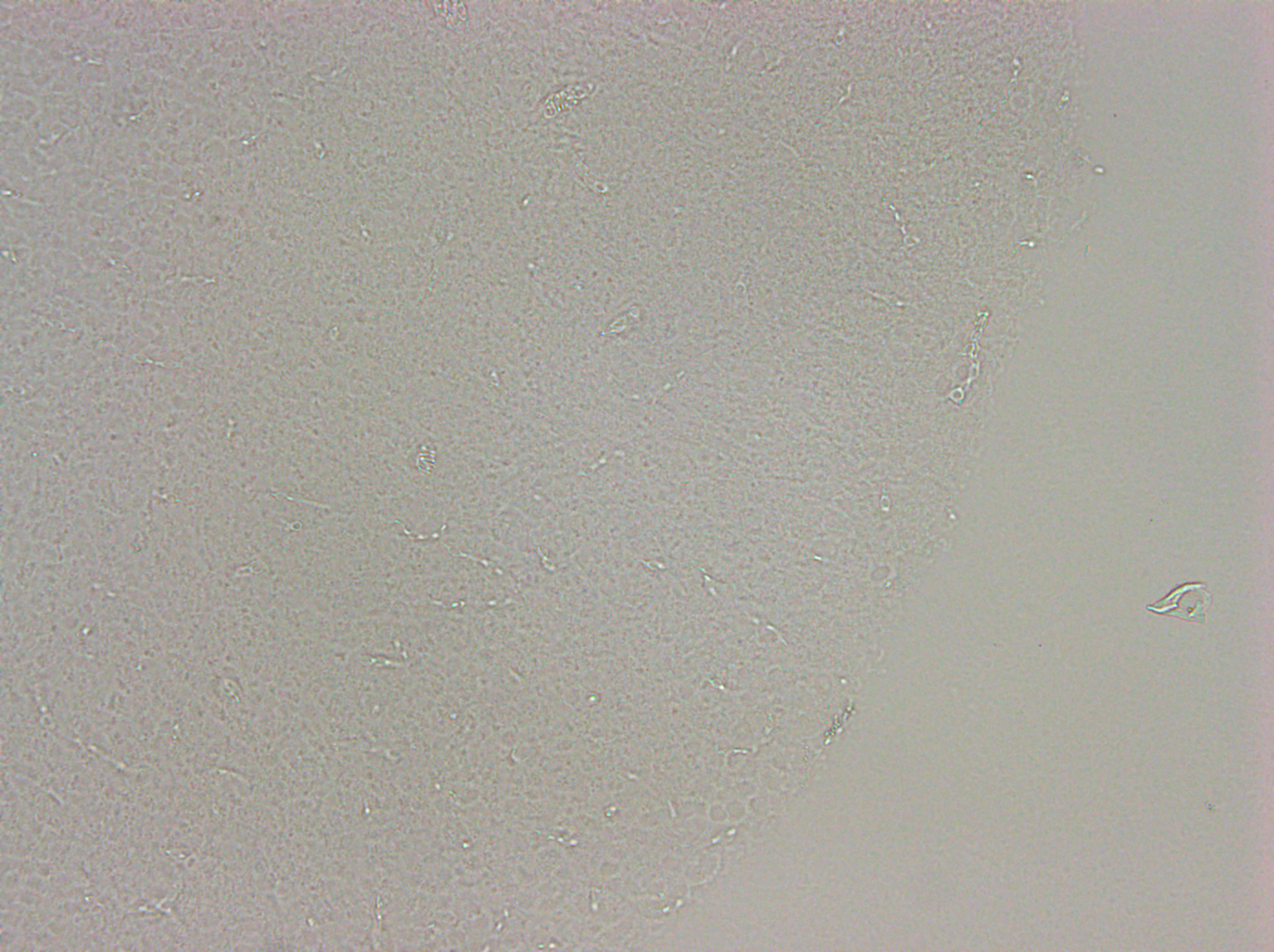

Supplement: Gene expression analysis in the adult zebrafish pallium — Dataset 1 Expression of eomesb in the embryonic brain and the adult pallium in zebrafish. Raw data of Figure S2 and additional image files of eomesb expression in the embryo and the adult pallium. Dataset 2 Images of negative control. No signal was detected in the absence of the riboprobe, demonstrating that the antibody reacts specifically with the synthetic RNA. Dataset 3 Expression of eomesa in the zebrafish pallium. Raw data of Figure 1 and additional image files of eomesa expression in the adult pallium. Dataset 4 Expression of emx1, emx2 and emx3 in the zebrafish larval brain. Raw data of Figure S3 and additional image files of emx gene expression in the zebrafish larvae. Dataset 5 Expression of emx1, emx2 and emx3 in the zebrafish pallium. Raw data of Figure 2 and additional image files of emx gene expression in the adult pallium. Dataset 6 Expression of Prox1 in the zebrafish pallium. Raw data of Figure 3 and additional image files of Prox1 expression in the adult pallium. Dataset 7 Expression of ascl1a in the zebrafish pallium. Raw data of Figure 4 and additional image files of ascl1a expression in the adult pallium. [file f1000research-3-7777-s0000.tgz › NegativeControl_2.tif]

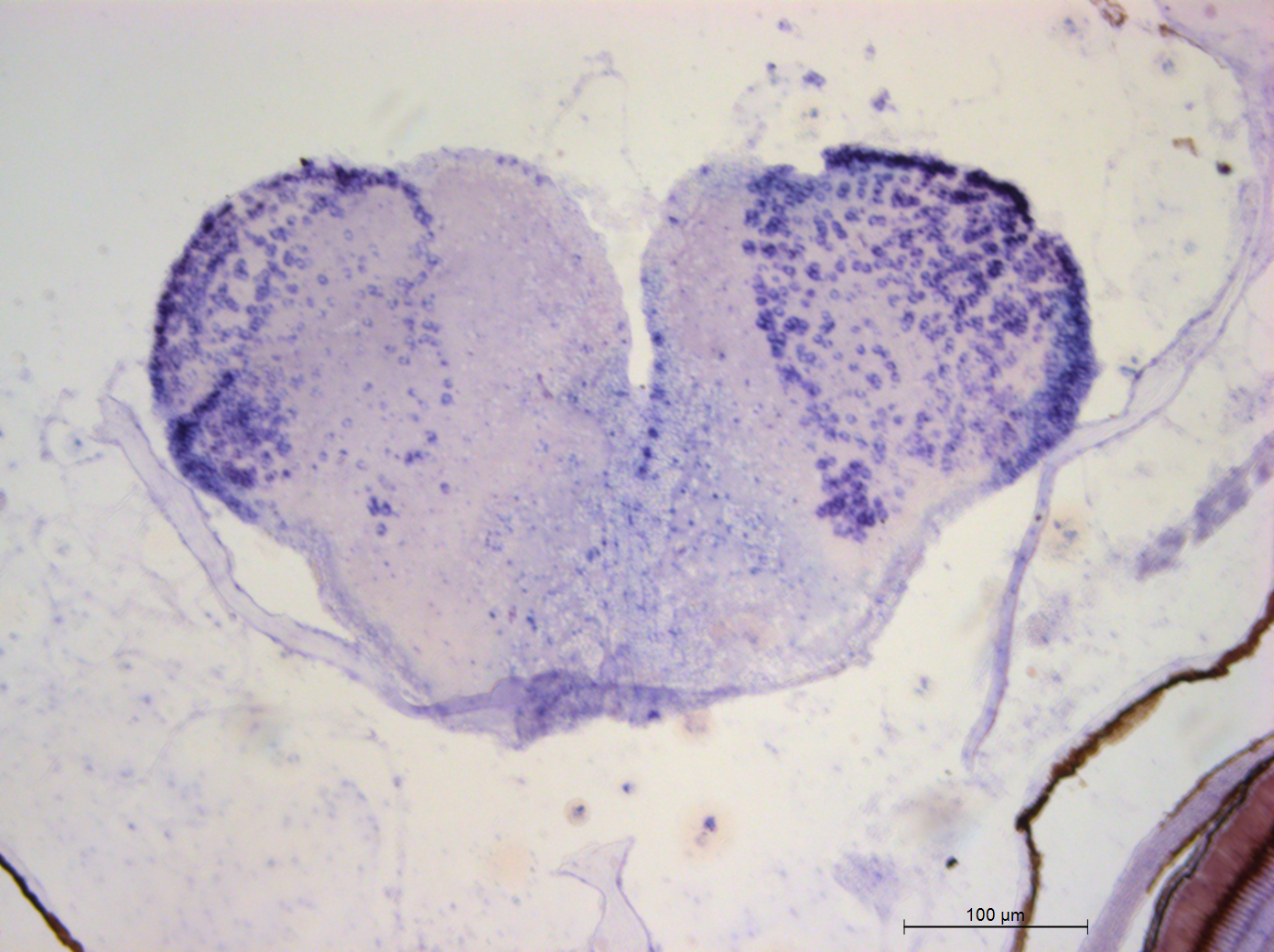

Supplement: Gene expression analysis in the adult zebrafish pallium — Dataset 1 Expression of eomesb in the embryonic brain and the adult pallium in zebrafish. Raw data of Figure S2 and additional image files of eomesb expression in the embryo and the adult pallium. Dataset 2 Images of negative control. No signal was detected in the absence of the riboprobe, demonstrating that the antibody reacts specifically with the synthetic RNA. Dataset 3 Expression of eomesa in the zebrafish pallium. Raw data of Figure 1 and additional image files of eomesa expression in the adult pallium. Dataset 4 Expression of emx1, emx2 and emx3 in the zebrafish larval brain. Raw data of Figure S3 and additional image files of emx gene expression in the zebrafish larvae. Dataset 5 Expression of emx1, emx2 and emx3 in the zebrafish pallium. Raw data of Figure 2 and additional image files of emx gene expression in the adult pallium. Dataset 6 Expression of Prox1 in the zebrafish pallium. Raw data of Figure 3 and additional image files of Prox1 expression in the adult pallium. Dataset 7 Expression of ascl1a in the zebrafish pallium. Raw data of Figure 4 and additional image files of ascl1a expression in the adult pallium. [file f1000research-3-7777-s0000.tgz › eomesa_2.tif]

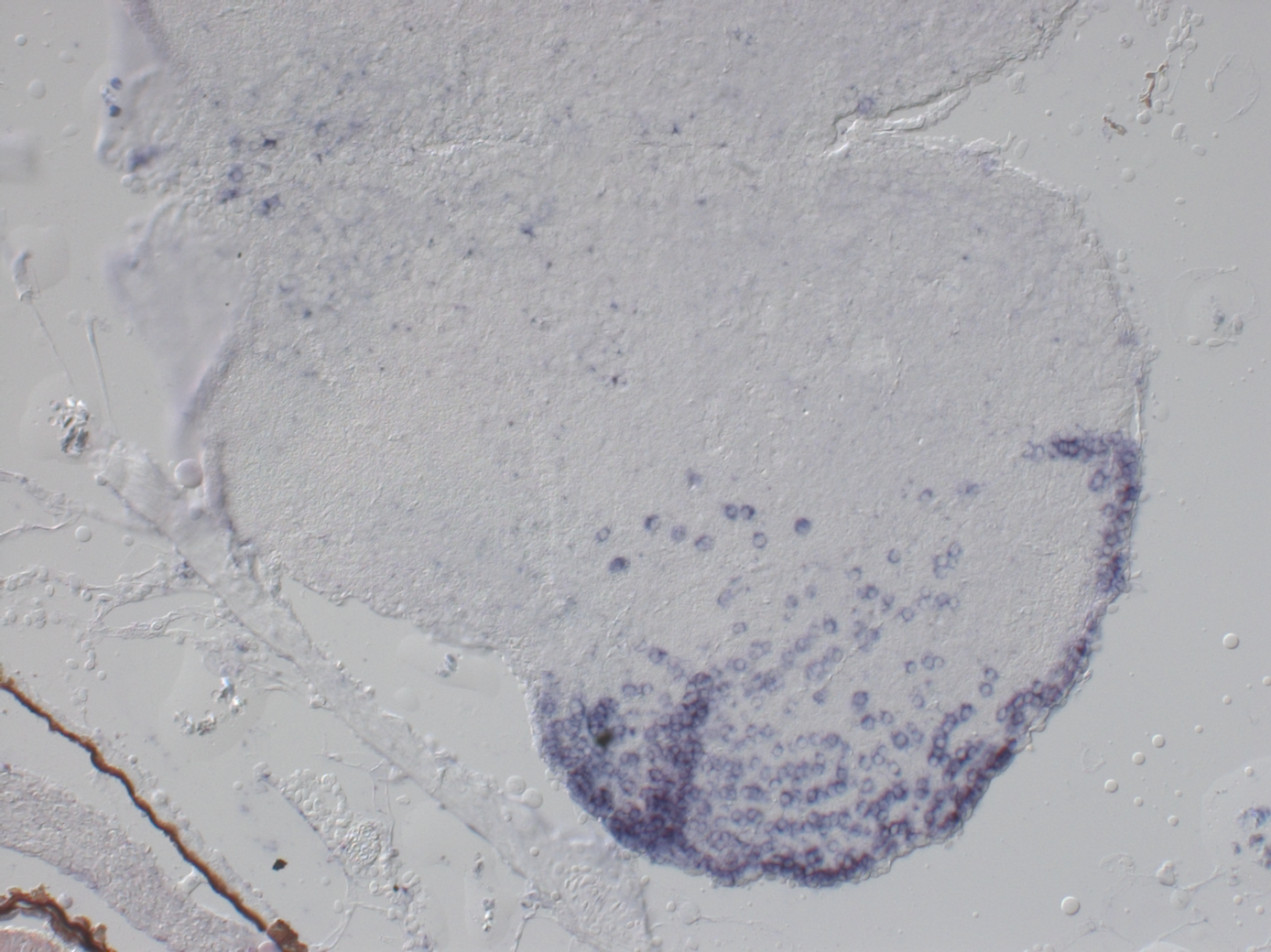

Supplement: Gene expression analysis in the adult zebrafish pallium — Dataset 1 Expression of eomesb in the embryonic brain and the adult pallium in zebrafish. Raw data of Figure S2 and additional image files of eomesb expression in the embryo and the adult pallium. Dataset 2 Images of negative control. No signal was detected in the absence of the riboprobe, demonstrating that the antibody reacts specifically with the synthetic RNA. Dataset 3 Expression of eomesa in the zebrafish pallium. Raw data of Figure 1 and additional image files of eomesa expression in the adult pallium. Dataset 4 Expression of emx1, emx2 and emx3 in the zebrafish larval brain. Raw data of Figure S3 and additional image files of emx gene expression in the zebrafish larvae. Dataset 5 Expression of emx1, emx2 and emx3 in the zebrafish pallium. Raw data of Figure 2 and additional image files of emx gene expression in the adult pallium. Dataset 6 Expression of Prox1 in the zebrafish pallium. Raw data of Figure 3 and additional image files of Prox1 expression in the adult pallium. Dataset 7 Expression of ascl1a in the zebrafish pallium. Raw data of Figure 4 and additional image files of ascl1a expression in the adult pallium. [file f1000research-3-7777-s0000.tgz › eomesa_3.TIF]

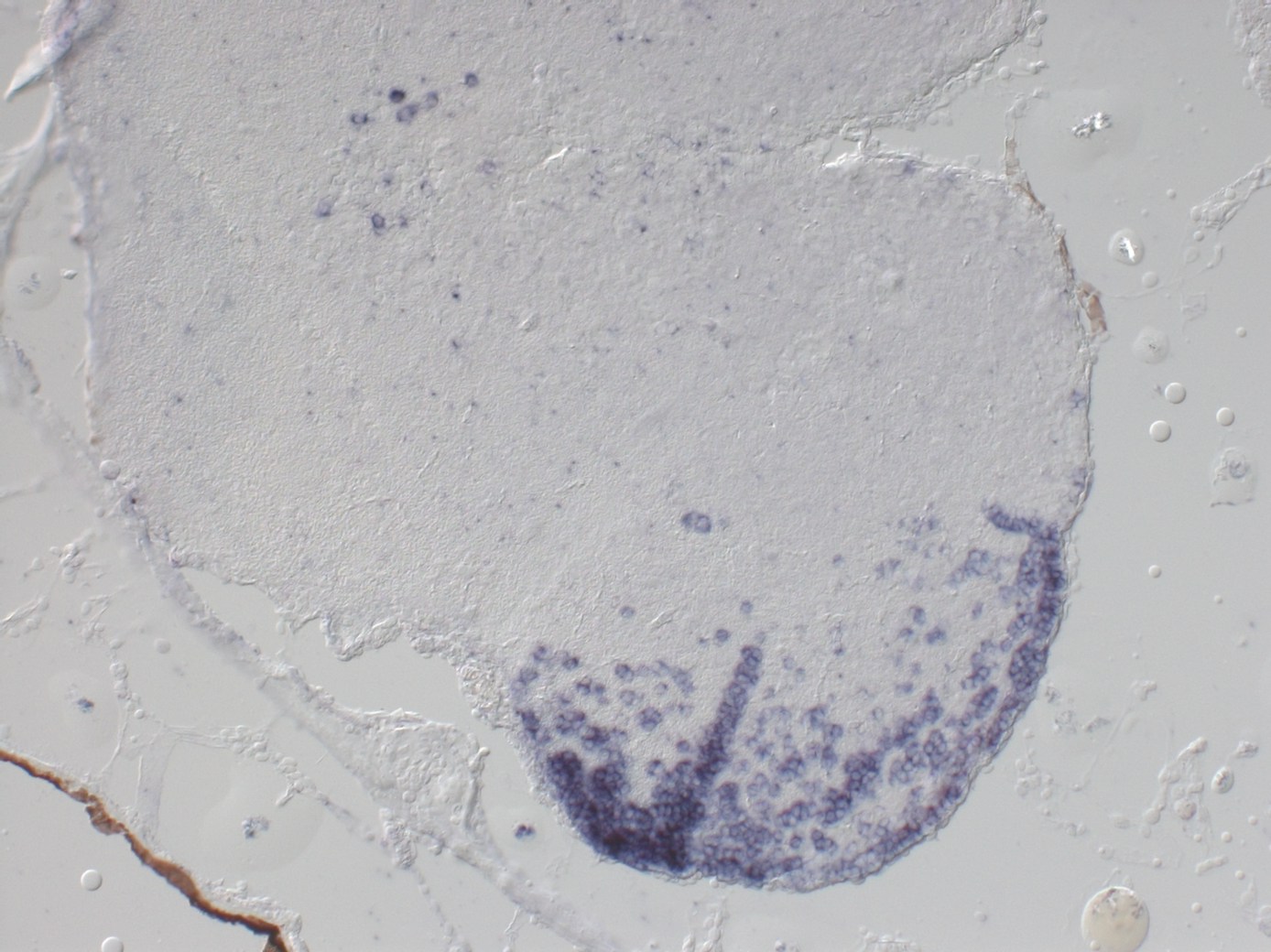

Supplement: Gene expression analysis in the adult zebrafish pallium — Dataset 1 Expression of eomesb in the embryonic brain and the adult pallium in zebrafish. Raw data of Figure S2 and additional image files of eomesb expression in the embryo and the adult pallium. Dataset 2 Images of negative control. No signal was detected in the absence of the riboprobe, demonstrating that the antibody reacts specifically with the synthetic RNA. Dataset 3 Expression of eomesa in the zebrafish pallium. Raw data of Figure 1 and additional image files of eomesa expression in the adult pallium. Dataset 4 Expression of emx1, emx2 and emx3 in the zebrafish larval brain. Raw data of Figure S3 and additional image files of emx gene expression in the zebrafish larvae. Dataset 5 Expression of emx1, emx2 and emx3 in the zebrafish pallium. Raw data of Figure 2 and additional image files of emx gene expression in the adult pallium. Dataset 6 Expression of Prox1 in the zebrafish pallium. Raw data of Figure 3 and additional image files of Prox1 expression in the adult pallium. Dataset 7 Expression of ascl1a in the zebrafish pallium. Raw data of Figure 4 and additional image files of ascl1a expression in the adult pallium. [file f1000research-3-7777-s0000.tgz › eomesa_4.TIF]

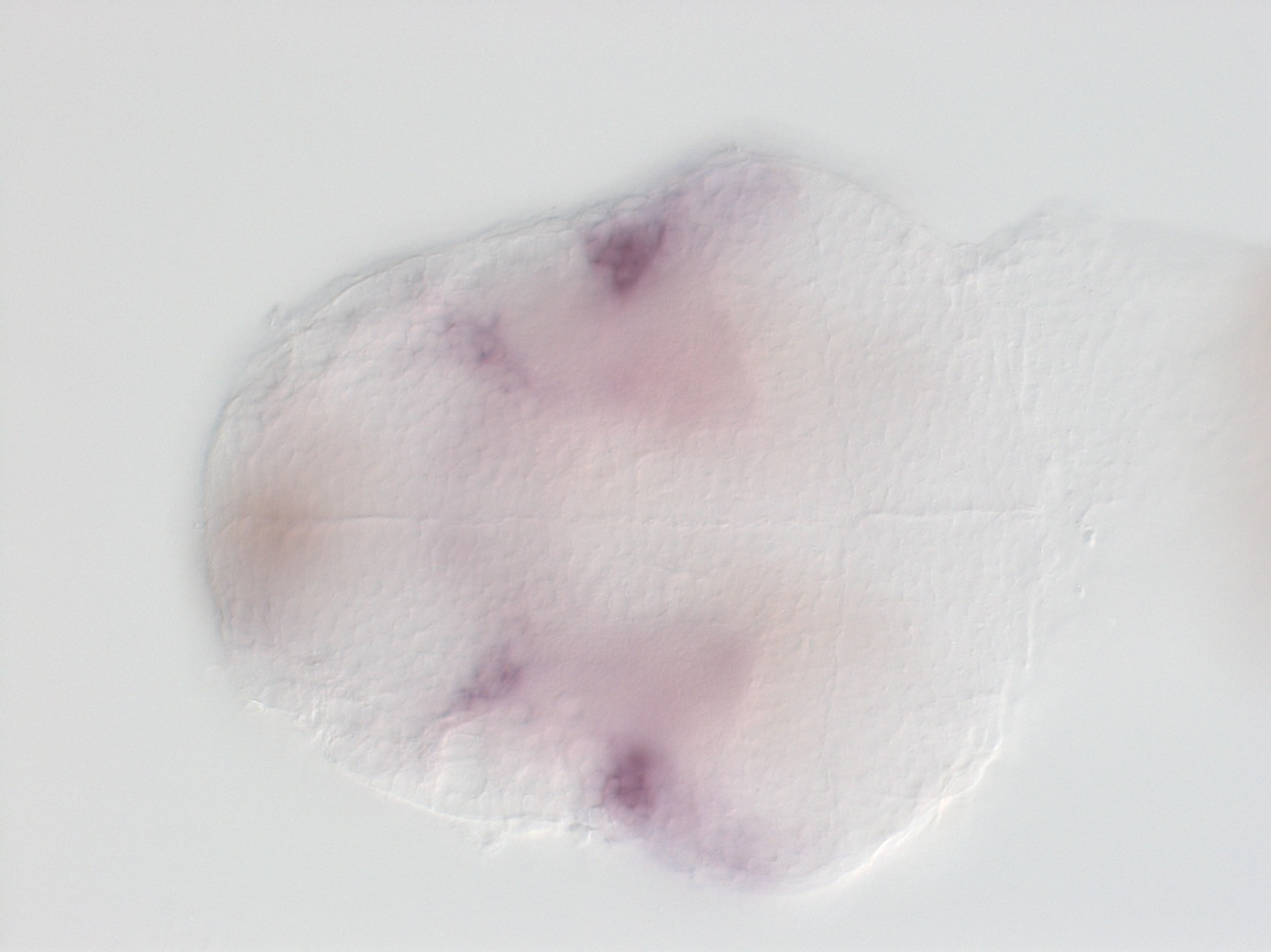

Supplement: Gene expression analysis in the adult zebrafish pallium — Dataset 1 Expression of eomesb in the embryonic brain and the adult pallium in zebrafish. Raw data of Figure S2 and additional image files of eomesb expression in the embryo and the adult pallium. Dataset 2 Images of negative control. No signal was detected in the absence of the riboprobe, demonstrating that the antibody reacts specifically with the synthetic RNA. Dataset 3 Expression of eomesa in the zebrafish pallium. Raw data of Figure 1 and additional image files of eomesa expression in the adult pallium. Dataset 4 Expression of emx1, emx2 and emx3 in the zebrafish larval brain. Raw data of Figure S3 and additional image files of emx gene expression in the zebrafish larvae. Dataset 5 Expression of emx1, emx2 and emx3 in the zebrafish pallium. Raw data of Figure 2 and additional image files of emx gene expression in the adult pallium. Dataset 6 Expression of Prox1 in the zebrafish pallium. Raw data of Figure 3 and additional image files of Prox1 expression in the adult pallium. Dataset 7 Expression of ascl1a in the zebrafish pallium. Raw data of Figure 4 and additional image files of ascl1a expression in the adult pallium. [file f1000research-3-7777-s0000.tgz › emx1_2.TIF]

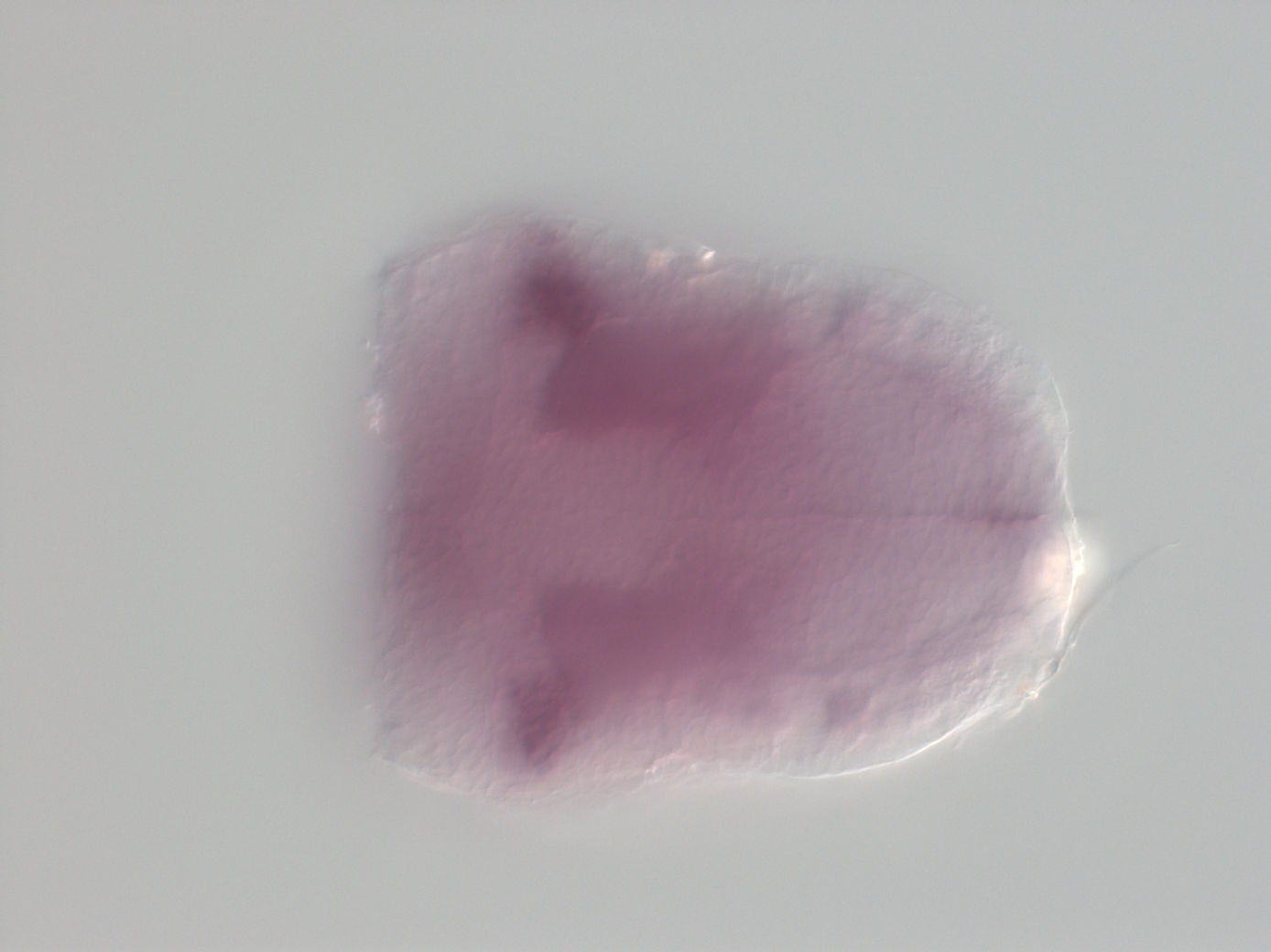

Supplement: Gene expression analysis in the adult zebrafish pallium — Dataset 1 Expression of eomesb in the embryonic brain and the adult pallium in zebrafish. Raw data of Figure S2 and additional image files of eomesb expression in the embryo and the adult pallium. Dataset 2 Images of negative control. No signal was detected in the absence of the riboprobe, demonstrating that the antibody reacts specifically with the synthetic RNA. Dataset 3 Expression of eomesa in the zebrafish pallium. Raw data of Figure 1 and additional image files of eomesa expression in the adult pallium. Dataset 4 Expression of emx1, emx2 and emx3 in the zebrafish larval brain. Raw data of Figure S3 and additional image files of emx gene expression in the zebrafish larvae. Dataset 5 Expression of emx1, emx2 and emx3 in the zebrafish pallium. Raw data of Figure 2 and additional image files of emx gene expression in the adult pallium. Dataset 6 Expression of Prox1 in the zebrafish pallium. Raw data of Figure 3 and additional image files of Prox1 expression in the adult pallium. Dataset 7 Expression of ascl1a in the zebrafish pallium. Raw data of Figure 4 and additional image files of ascl1a expression in the adult pallium. [file f1000research-3-7777-s0000.tgz › emx1_1.TIF]

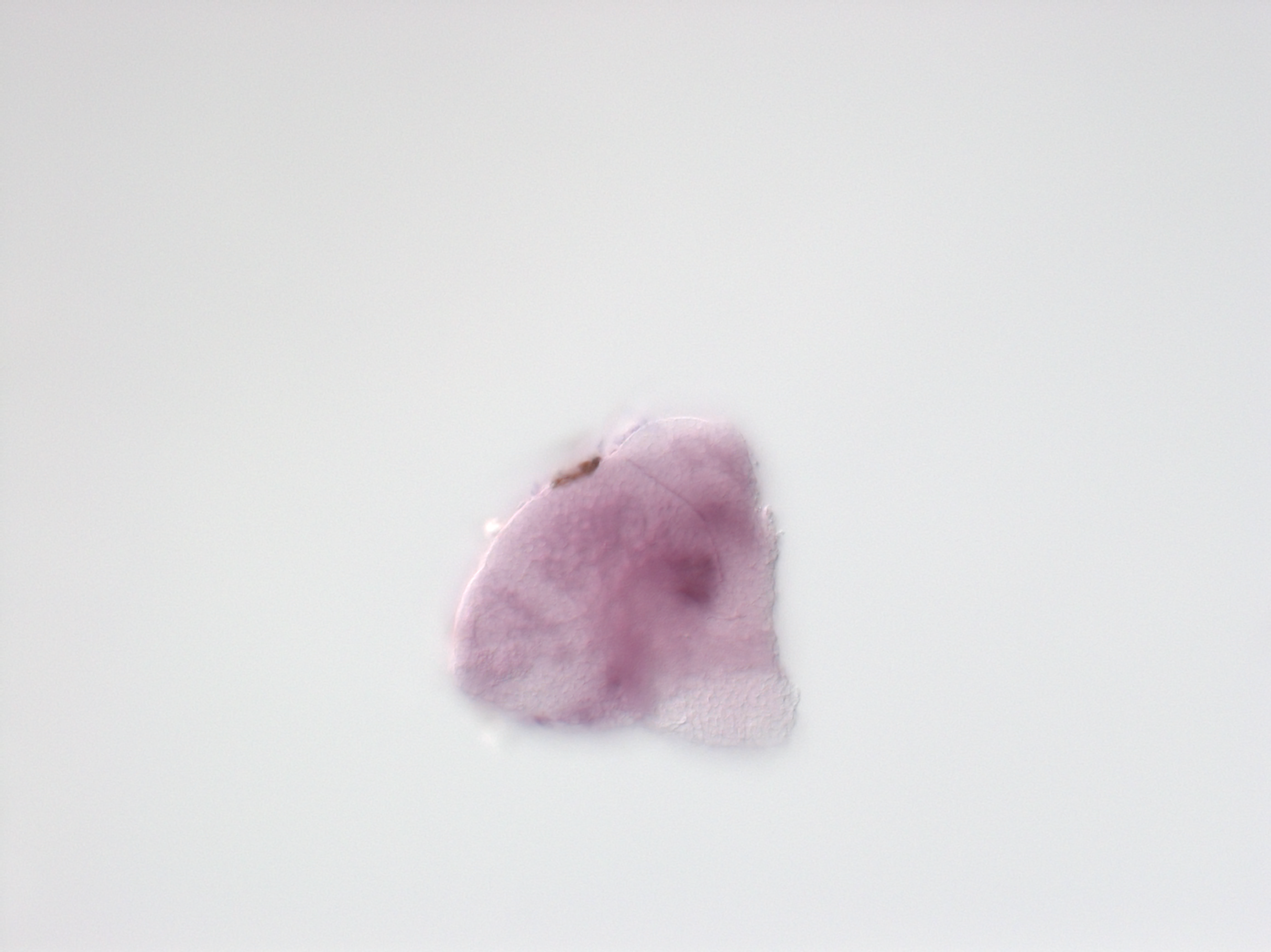

Supplement: Gene expression analysis in the adult zebrafish pallium — Dataset 1 Expression of eomesb in the embryonic brain and the adult pallium in zebrafish. Raw data of Figure S2 and additional image files of eomesb expression in the embryo and the adult pallium. Dataset 2 Images of negative control. No signal was detected in the absence of the riboprobe, demonstrating that the antibody reacts specifically with the synthetic RNA. Dataset 3 Expression of eomesa in the zebrafish pallium. Raw data of Figure 1 and additional image files of eomesa expression in the adult pallium. Dataset 4 Expression of emx1, emx2 and emx3 in the zebrafish larval brain. Raw data of Figure S3 and additional image files of emx gene expression in the zebrafish larvae. Dataset 5 Expression of emx1, emx2 and emx3 in the zebrafish pallium. Raw data of Figure 2 and additional image files of emx gene expression in the adult pallium. Dataset 6 Expression of Prox1 in the zebrafish pallium. Raw data of Figure 3 and additional image files of Prox1 expression in the adult pallium. Dataset 7 Expression of ascl1a in the zebrafish pallium. Raw data of Figure 4 and additional image files of ascl1a expression in the adult pallium. [file f1000research-3-7777-s0000.tgz › emx2.TIF]

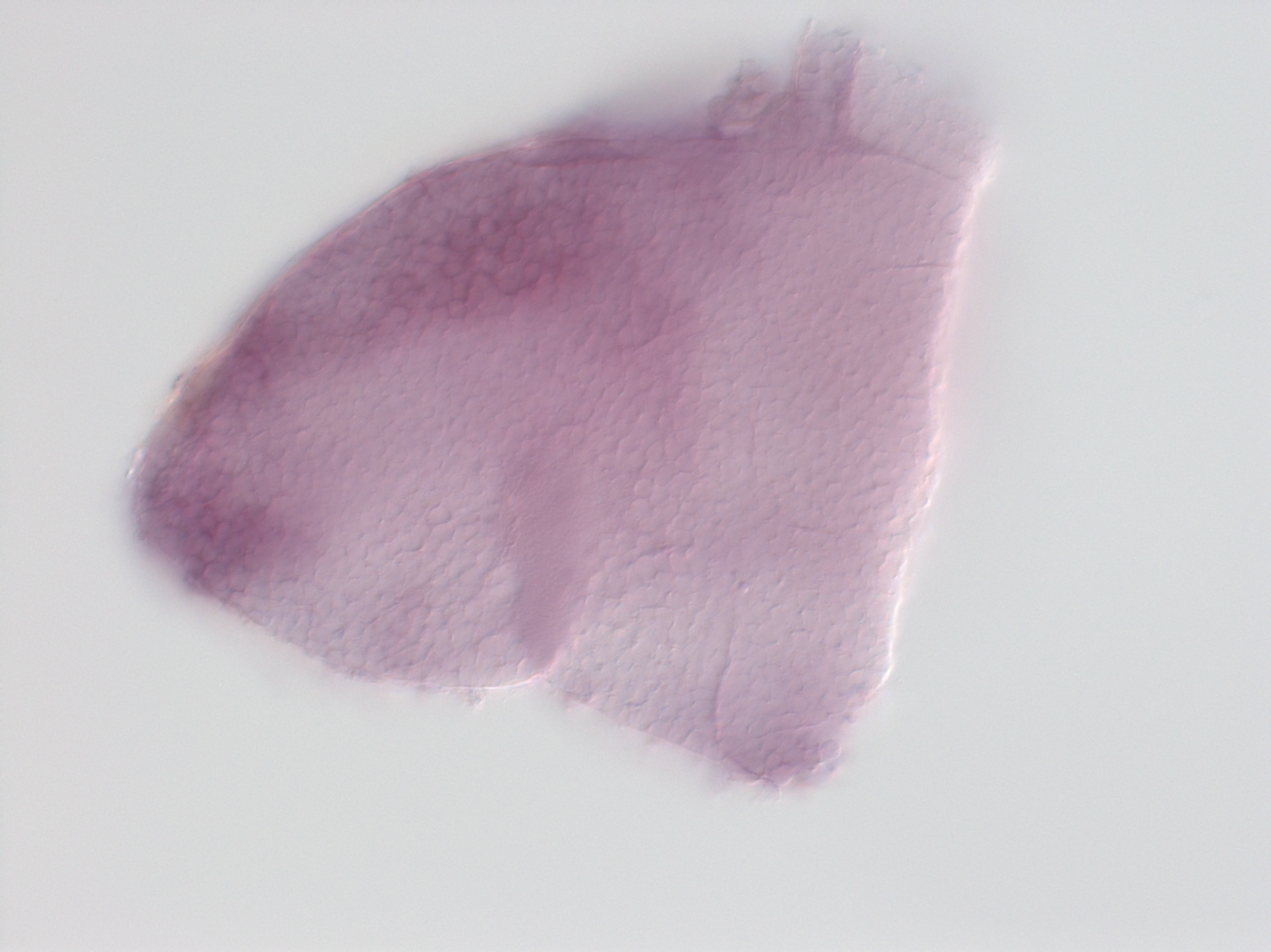

Supplement: Gene expression analysis in the adult zebrafish pallium — Dataset 1 Expression of eomesb in the embryonic brain and the adult pallium in zebrafish. Raw data of Figure S2 and additional image files of eomesb expression in the embryo and the adult pallium. Dataset 2 Images of negative control. No signal was detected in the absence of the riboprobe, demonstrating that the antibody reacts specifically with the synthetic RNA. Dataset 3 Expression of eomesa in the zebrafish pallium. Raw data of Figure 1 and additional image files of eomesa expression in the adult pallium. Dataset 4 Expression of emx1, emx2 and emx3 in the zebrafish larval brain. Raw data of Figure S3 and additional image files of emx gene expression in the zebrafish larvae. Dataset 5 Expression of emx1, emx2 and emx3 in the zebrafish pallium. Raw data of Figure 2 and additional image files of emx gene expression in the adult pallium. Dataset 6 Expression of Prox1 in the zebrafish pallium. Raw data of Figure 3 and additional image files of Prox1 expression in the adult pallium. Dataset 7 Expression of ascl1a in the zebrafish pallium. Raw data of Figure 4 and additional image files of ascl1a expression in the adult pallium. [file f1000research-3-7777-s0000.tgz › emx3.TIF]

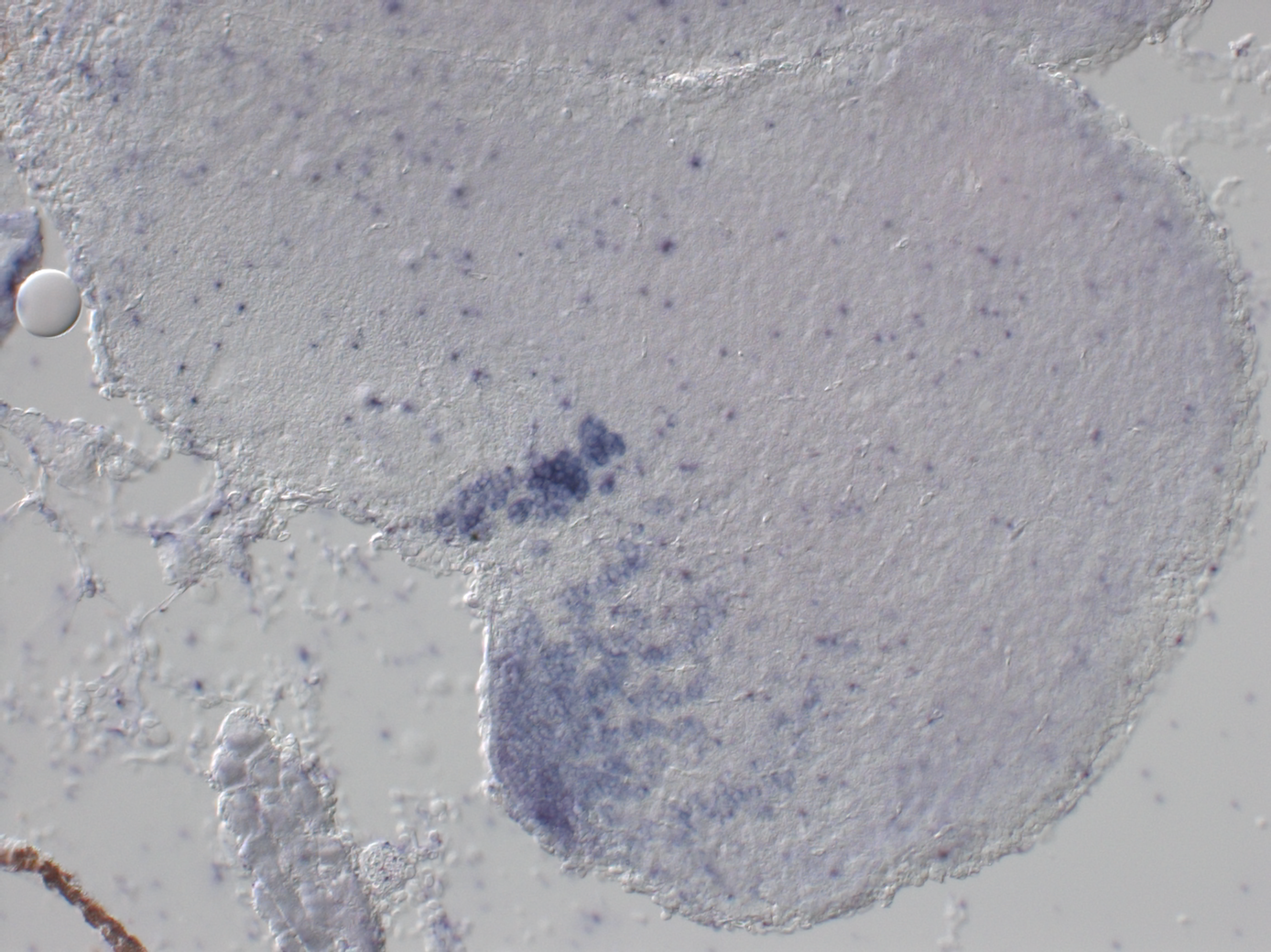

Supplement: Gene expression analysis in the adult zebrafish pallium — Dataset 1 Expression of eomesb in the embryonic brain and the adult pallium in zebrafish. Raw data of Figure S2 and additional image files of eomesb expression in the embryo and the adult pallium. Dataset 2 Images of negative control. No signal was detected in the absence of the riboprobe, demonstrating that the antibody reacts specifically with the synthetic RNA. Dataset 3 Expression of eomesa in the zebrafish pallium. Raw data of Figure 1 and additional image files of eomesa expression in the adult pallium. Dataset 4 Expression of emx1, emx2 and emx3 in the zebrafish larval brain. Raw data of Figure S3 and additional image files of emx gene expression in the zebrafish larvae. Dataset 5 Expression of emx1, emx2 and emx3 in the zebrafish pallium. Raw data of Figure 2 and additional image files of emx gene expression in the adult pallium. Dataset 6 Expression of Prox1 in the zebrafish pallium. Raw data of Figure 3 and additional image files of Prox1 expression in the adult pallium. Dataset 7 Expression of ascl1a in the zebrafish pallium. Raw data of Figure 4 and additional image files of ascl1a expression in the adult pallium. [file f1000research-3-7777-s0000.tgz › emx1.TIF]

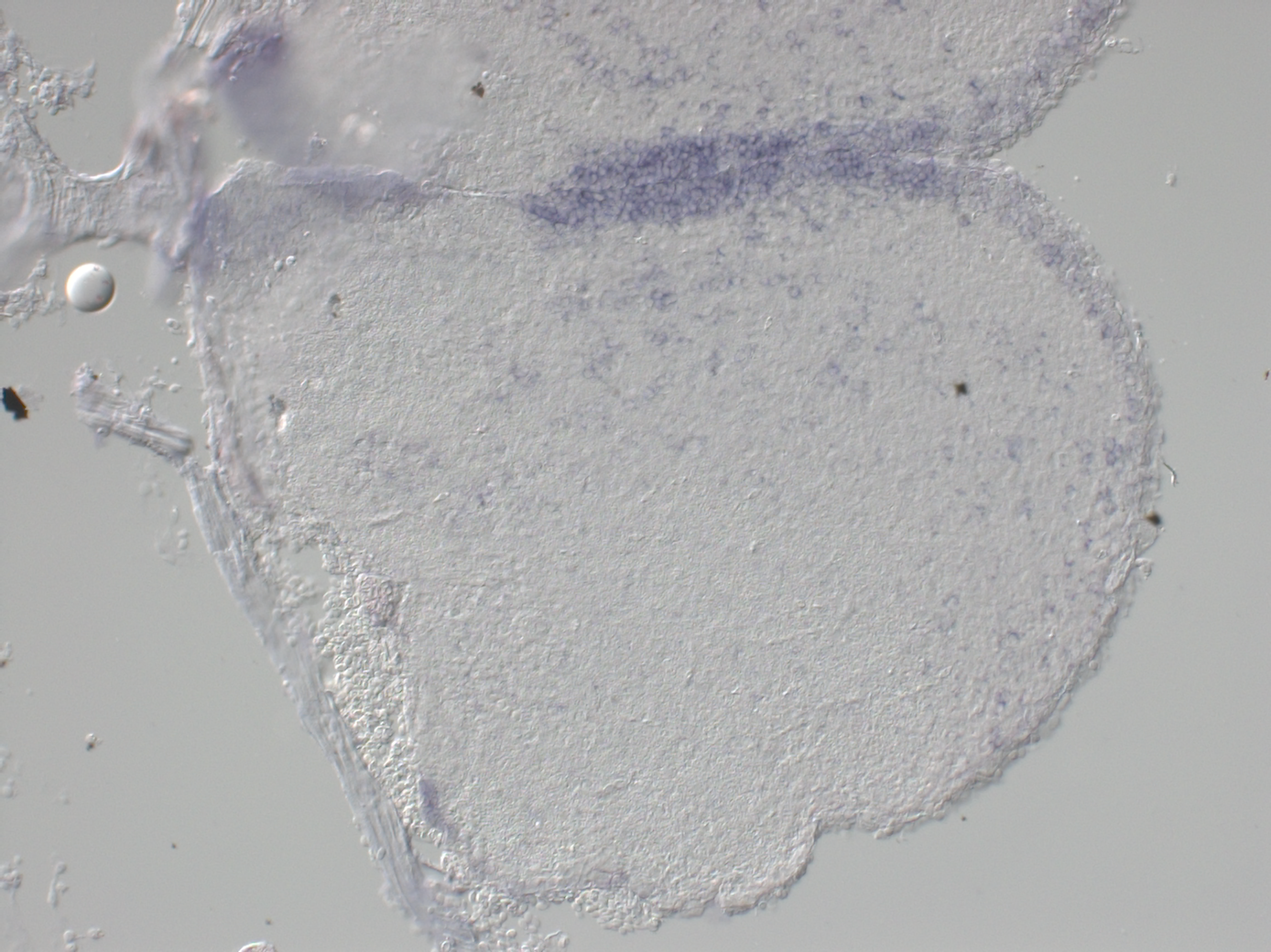

Supplement: Gene expression analysis in the adult zebrafish pallium — Dataset 1 Expression of eomesb in the embryonic brain and the adult pallium in zebrafish. Raw data of Figure S2 and additional image files of eomesb expression in the embryo and the adult pallium. Dataset 2 Images of negative control. No signal was detected in the absence of the riboprobe, demonstrating that the antibody reacts specifically with the synthetic RNA. Dataset 3 Expression of eomesa in the zebrafish pallium. Raw data of Figure 1 and additional image files of eomesa expression in the adult pallium. Dataset 4 Expression of emx1, emx2 and emx3 in the zebrafish larval brain. Raw data of Figure S3 and additional image files of emx gene expression in the zebrafish larvae. Dataset 5 Expression of emx1, emx2 and emx3 in the zebrafish pallium. Raw data of Figure 2 and additional image files of emx gene expression in the adult pallium. Dataset 6 Expression of Prox1 in the zebrafish pallium. Raw data of Figure 3 and additional image files of Prox1 expression in the adult pallium. Dataset 7 Expression of ascl1a in the zebrafish pallium. Raw data of Figure 4 and additional image files of ascl1a expression in the adult pallium. [file f1000research-3-7777-s0000.tgz › emx3_1.TIF]

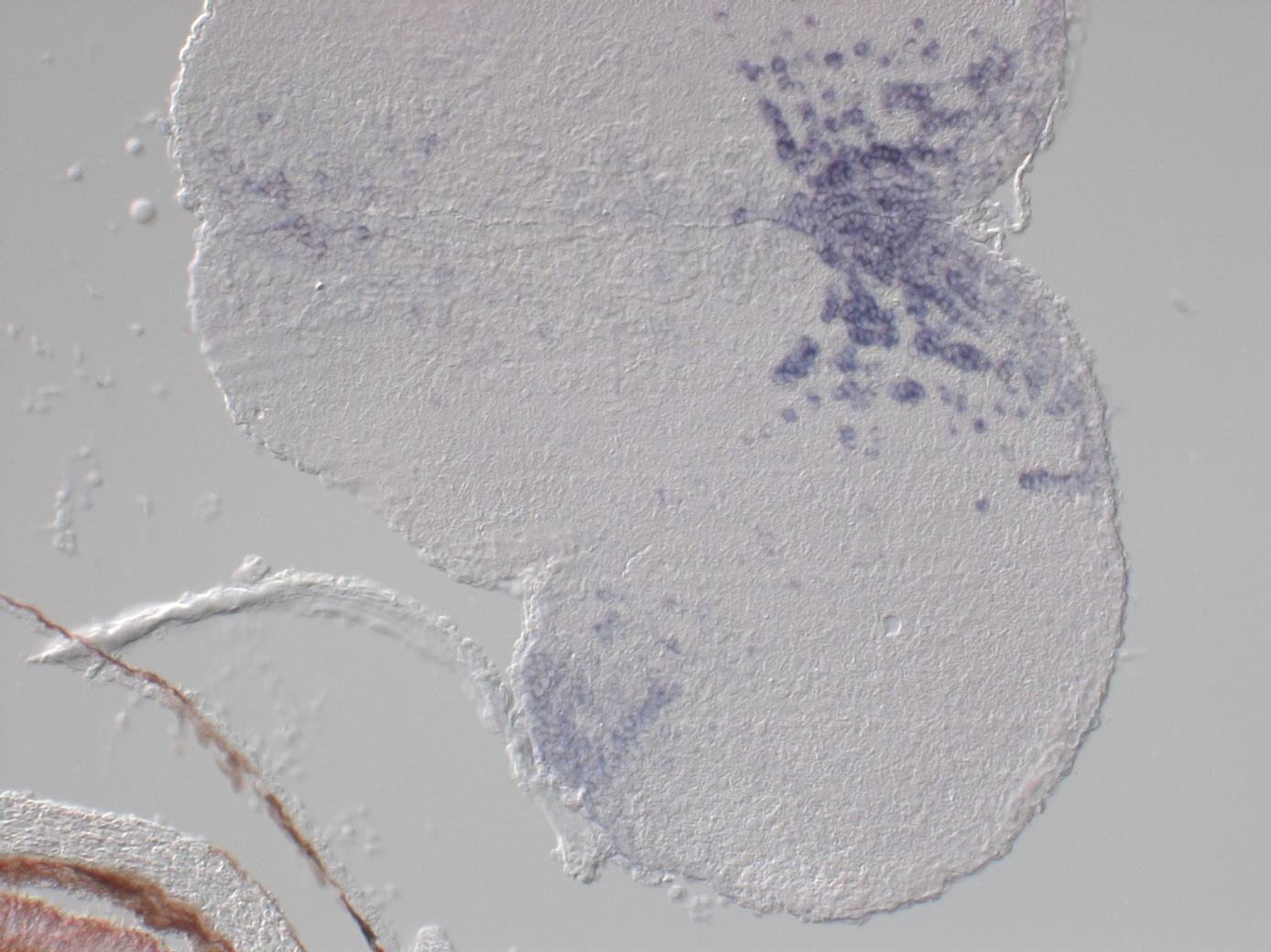

Supplement: Gene expression analysis in the adult zebrafish pallium — Dataset 1 Expression of eomesb in the embryonic brain and the adult pallium in zebrafish. Raw data of Figure S2 and additional image files of eomesb expression in the embryo and the adult pallium. Dataset 2 Images of negative control. No signal was detected in the absence of the riboprobe, demonstrating that the antibody reacts specifically with the synthetic RNA. Dataset 3 Expression of eomesa in the zebrafish pallium. Raw data of Figure 1 and additional image files of eomesa expression in the adult pallium. Dataset 4 Expression of emx1, emx2 and emx3 in the zebrafish larval brain. Raw data of Figure S3 and additional image files of emx gene expression in the zebrafish larvae. Dataset 5 Expression of emx1, emx2 and emx3 in the zebrafish pallium. Raw data of Figure 2 and additional image files of emx gene expression in the adult pallium. Dataset 6 Expression of Prox1 in the zebrafish pallium. Raw data of Figure 3 and additional image files of Prox1 expression in the adult pallium. Dataset 7 Expression of ascl1a in the zebrafish pallium. Raw data of Figure 4 and additional image files of ascl1a expression in the adult pallium. [file f1000research-3-7777-s0000.tgz › emx3_2.TIF]

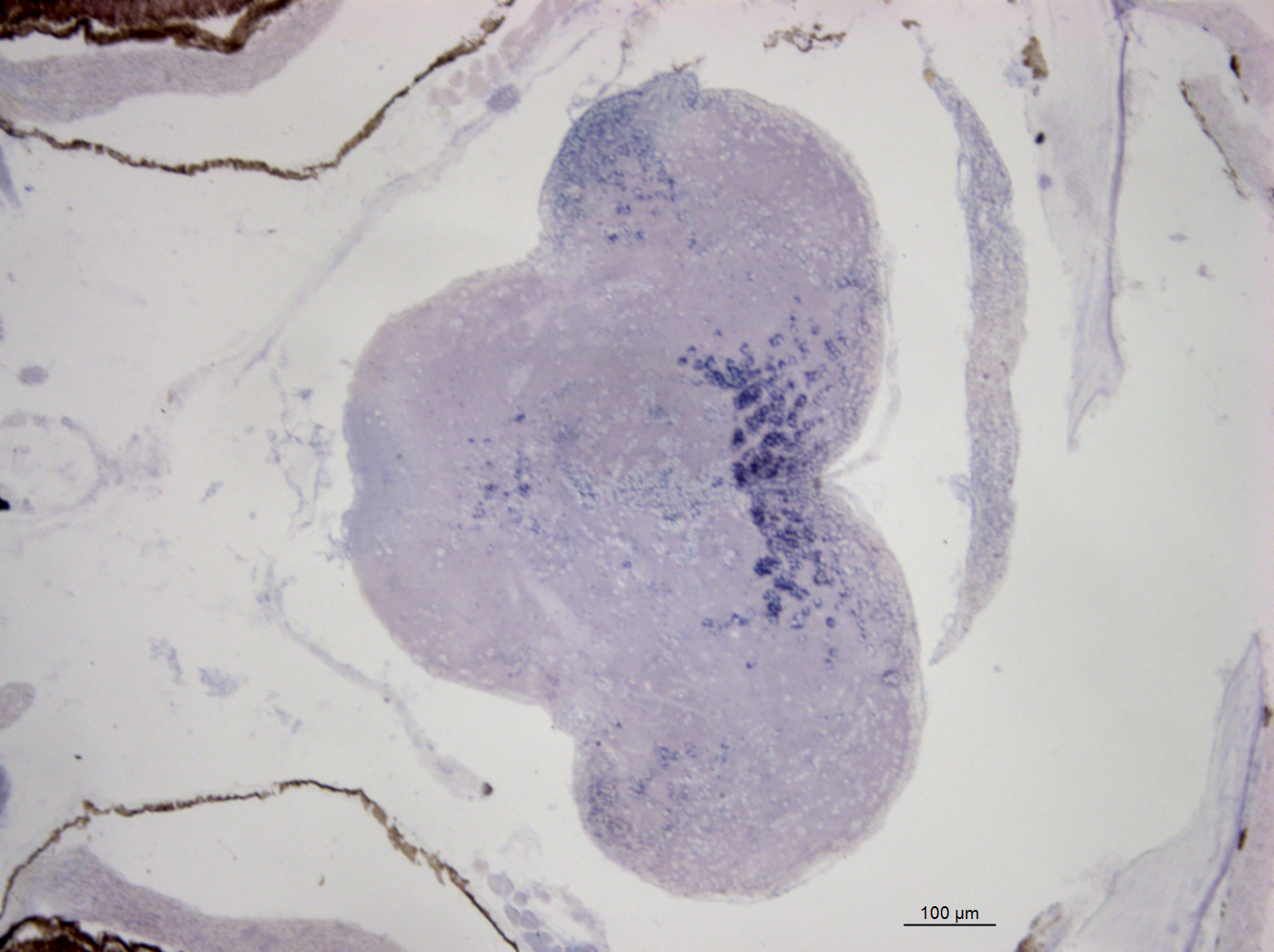

Supplement: Gene expression analysis in the adult zebrafish pallium — Dataset 1 Expression of eomesb in the embryonic brain and the adult pallium in zebrafish. Raw data of Figure S2 and additional image files of eomesb expression in the embryo and the adult pallium. Dataset 2 Images of negative control. No signal was detected in the absence of the riboprobe, demonstrating that the antibody reacts specifically with the synthetic RNA. Dataset 3 Expression of eomesa in the zebrafish pallium. Raw data of Figure 1 and additional image files of eomesa expression in the adult pallium. Dataset 4 Expression of emx1, emx2 and emx3 in the zebrafish larval brain. Raw data of Figure S3 and additional image files of emx gene expression in the zebrafish larvae. Dataset 5 Expression of emx1, emx2 and emx3 in the zebrafish pallium. Raw data of Figure 2 and additional image files of emx gene expression in the adult pallium. Dataset 6 Expression of Prox1 in the zebrafish pallium. Raw data of Figure 3 and additional image files of Prox1 expression in the adult pallium. Dataset 7 Expression of ascl1a in the zebrafish pallium. Raw data of Figure 4 and additional image files of ascl1a expression in the adult pallium. [file f1000research-3-7777-s0000.tgz › emx3_3.tif]

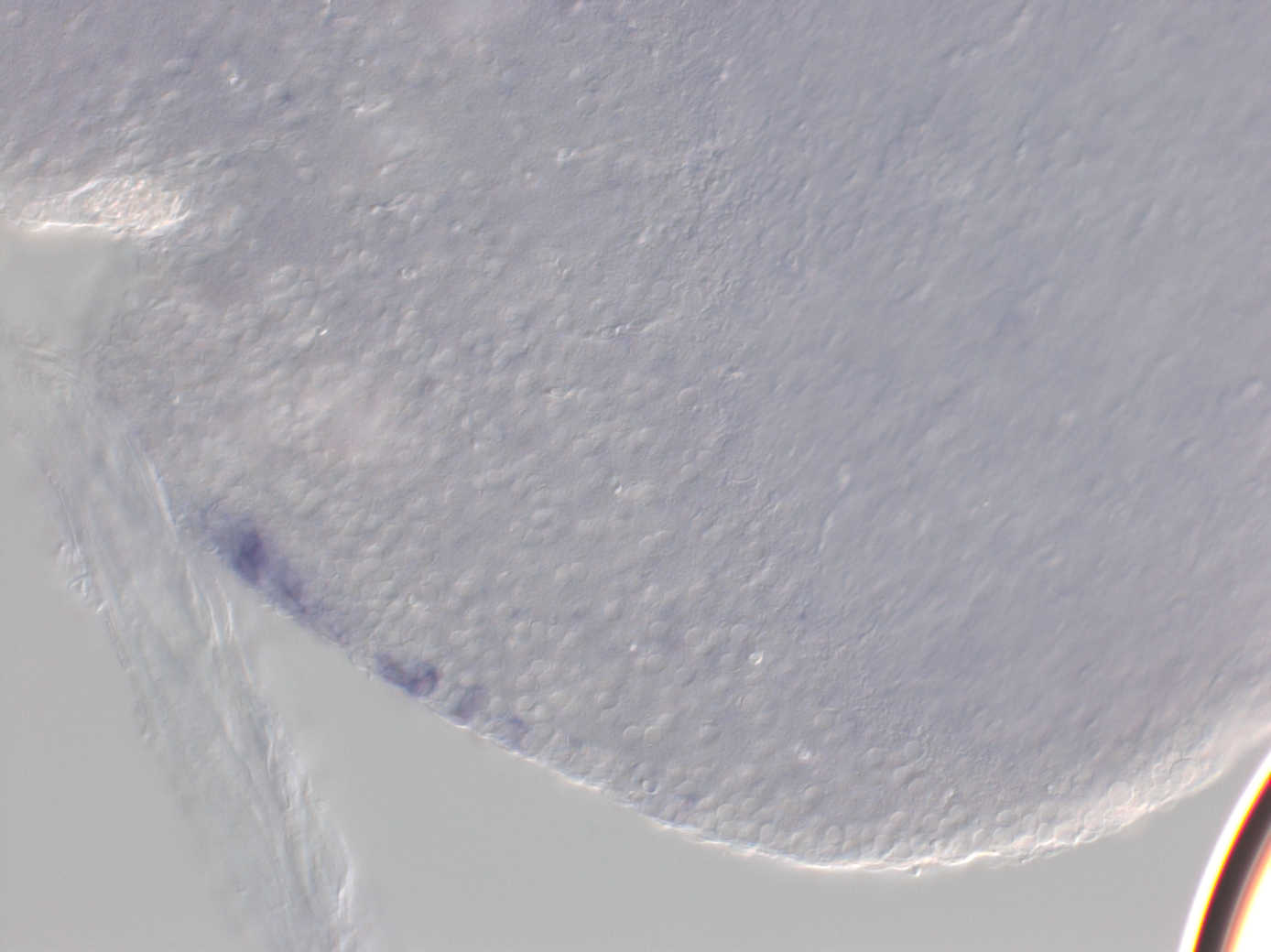

Supplement: Gene expression analysis in the adult zebrafish pallium — Dataset 1 Expression of eomesb in the embryonic brain and the adult pallium in zebrafish. Raw data of Figure S2 and additional image files of eomesb expression in the embryo and the adult pallium. Dataset 2 Images of negative control. No signal was detected in the absence of the riboprobe, demonstrating that the antibody reacts specifically with the synthetic RNA. Dataset 3 Expression of eomesa in the zebrafish pallium. Raw data of Figure 1 and additional image files of eomesa expression in the adult pallium. Dataset 4 Expression of emx1, emx2 and emx3 in the zebrafish larval brain. Raw data of Figure S3 and additional image files of emx gene expression in the zebrafish larvae. Dataset 5 Expression of emx1, emx2 and emx3 in the zebrafish pallium. Raw data of Figure 2 and additional image files of emx gene expression in the adult pallium. Dataset 6 Expression of Prox1 in the zebrafish pallium. Raw data of Figure 3 and additional image files of Prox1 expression in the adult pallium. Dataset 7 Expression of ascl1a in the zebrafish pallium. Raw data of Figure 4 and additional image files of ascl1a expression in the adult pallium. [file f1000research-3-7777-s0000.tgz › ascl1a_2.TIF]

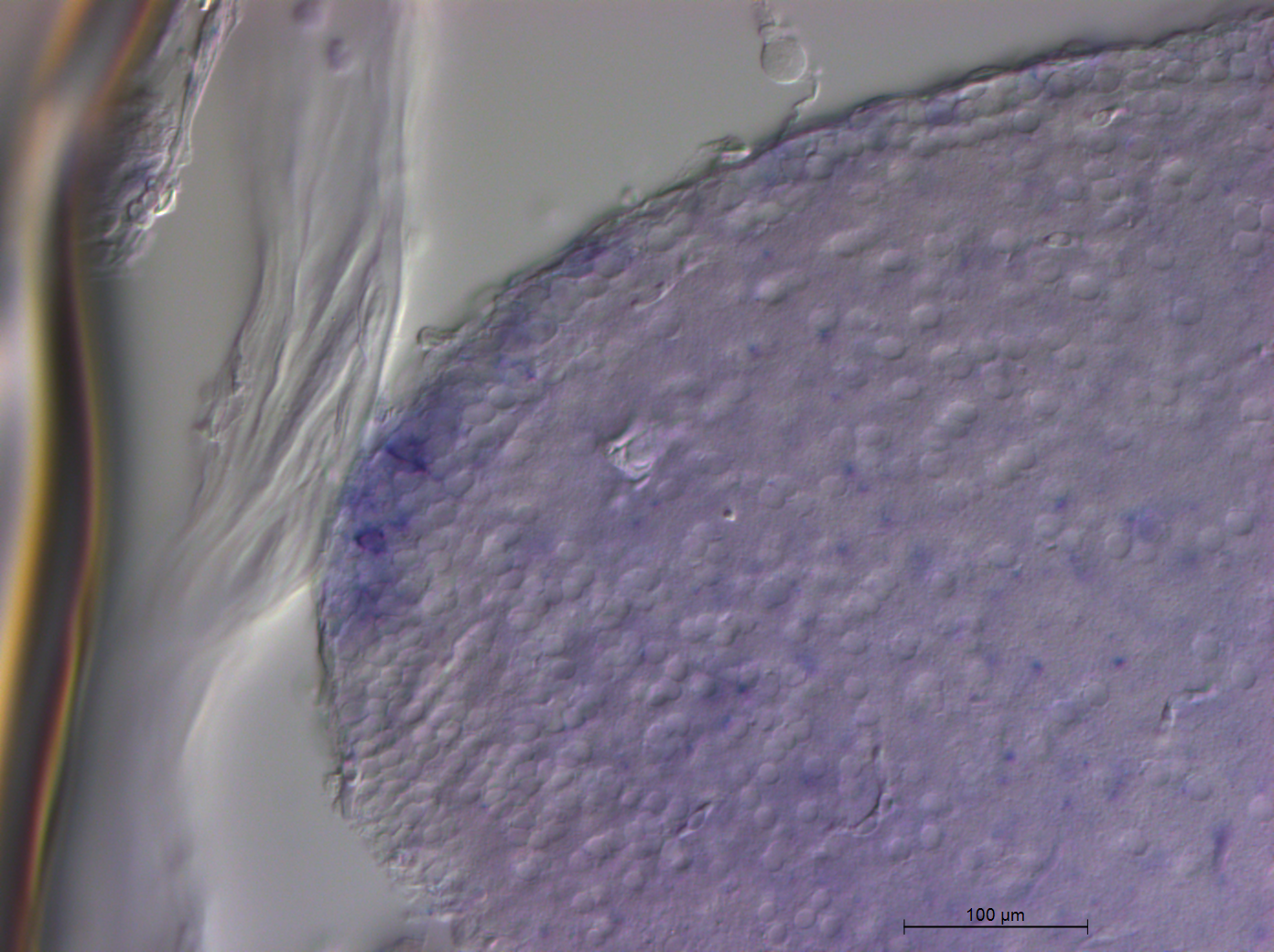

Supplement: Gene expression analysis in the adult zebrafish pallium — Dataset 1 Expression of eomesb in the embryonic brain and the adult pallium in zebrafish. Raw data of Figure S2 and additional image files of eomesb expression in the embryo and the adult pallium. Dataset 2 Images of negative control. No signal was detected in the absence of the riboprobe, demonstrating that the antibody reacts specifically with the synthetic RNA. Dataset 3 Expression of eomesa in the zebrafish pallium. Raw data of Figure 1 and additional image files of eomesa expression in the adult pallium. Dataset 4 Expression of emx1, emx2 and emx3 in the zebrafish larval brain. Raw data of Figure S3 and additional image files of emx gene expression in the zebrafish larvae. Dataset 5 Expression of emx1, emx2 and emx3 in the zebrafish pallium. Raw data of Figure 2 and additional image files of emx gene expression in the adult pallium. Dataset 6 Expression of Prox1 in the zebrafish pallium. Raw data of Figure 3 and additional image files of Prox1 expression in the adult pallium. Dataset 7 Expression of ascl1a in the zebrafish pallium. Raw data of Figure 4 and additional image files of ascl1a expression in the adult pallium. [file f1000research-3-7777-s0000.tgz › ascl1a_3.tif]

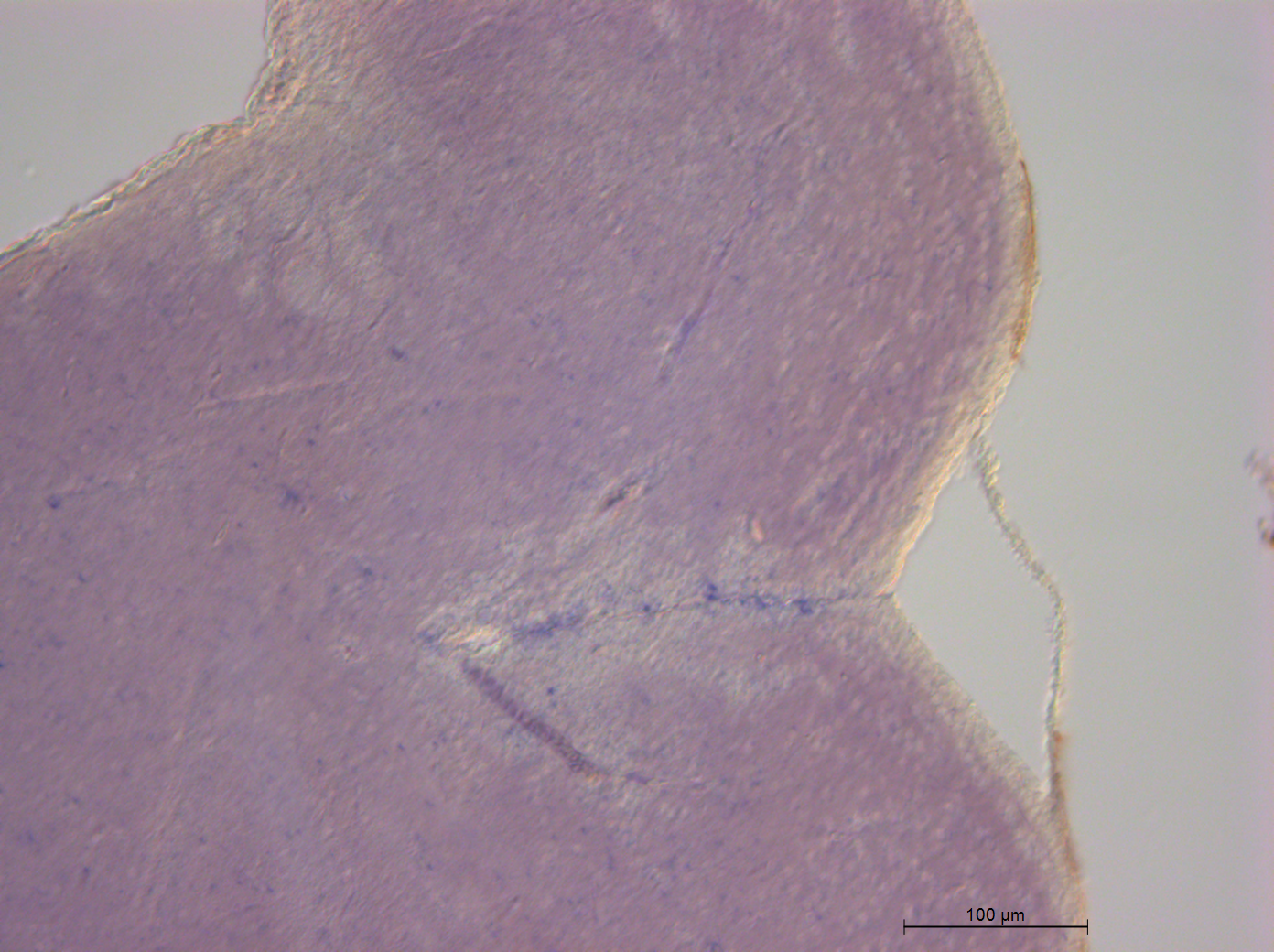

Supplement: Gene expression analysis in the adult zebrafish pallium — Dataset 1 Expression of eomesb in the embryonic brain and the adult pallium in zebrafish. Raw data of Figure S2 and additional image files of eomesb expression in the embryo and the adult pallium. Dataset 2 Images of negative control. No signal was detected in the absence of the riboprobe, demonstrating that the antibody reacts specifically with the synthetic RNA. Dataset 3 Expression of eomesa in the zebrafish pallium. Raw data of Figure 1 and additional image files of eomesa expression in the adult pallium. Dataset 4 Expression of emx1, emx2 and emx3 in the zebrafish larval brain. Raw data of Figure S3 and additional image files of emx gene expression in the zebrafish larvae. Dataset 5 Expression of emx1, emx2 and emx3 in the zebrafish pallium. Raw data of Figure 2 and additional image files of emx gene expression in the adult pallium. Dataset 6 Expression of Prox1 in the zebrafish pallium. Raw data of Figure 3 and additional image files of Prox1 expression in the adult pallium. Dataset 7 Expression of ascl1a in the zebrafish pallium. Raw data of Figure 4 and additional image files of ascl1a expression in the adult pallium. [file f1000research-3-7777-s0000.tgz › ascl1a_4.tif]

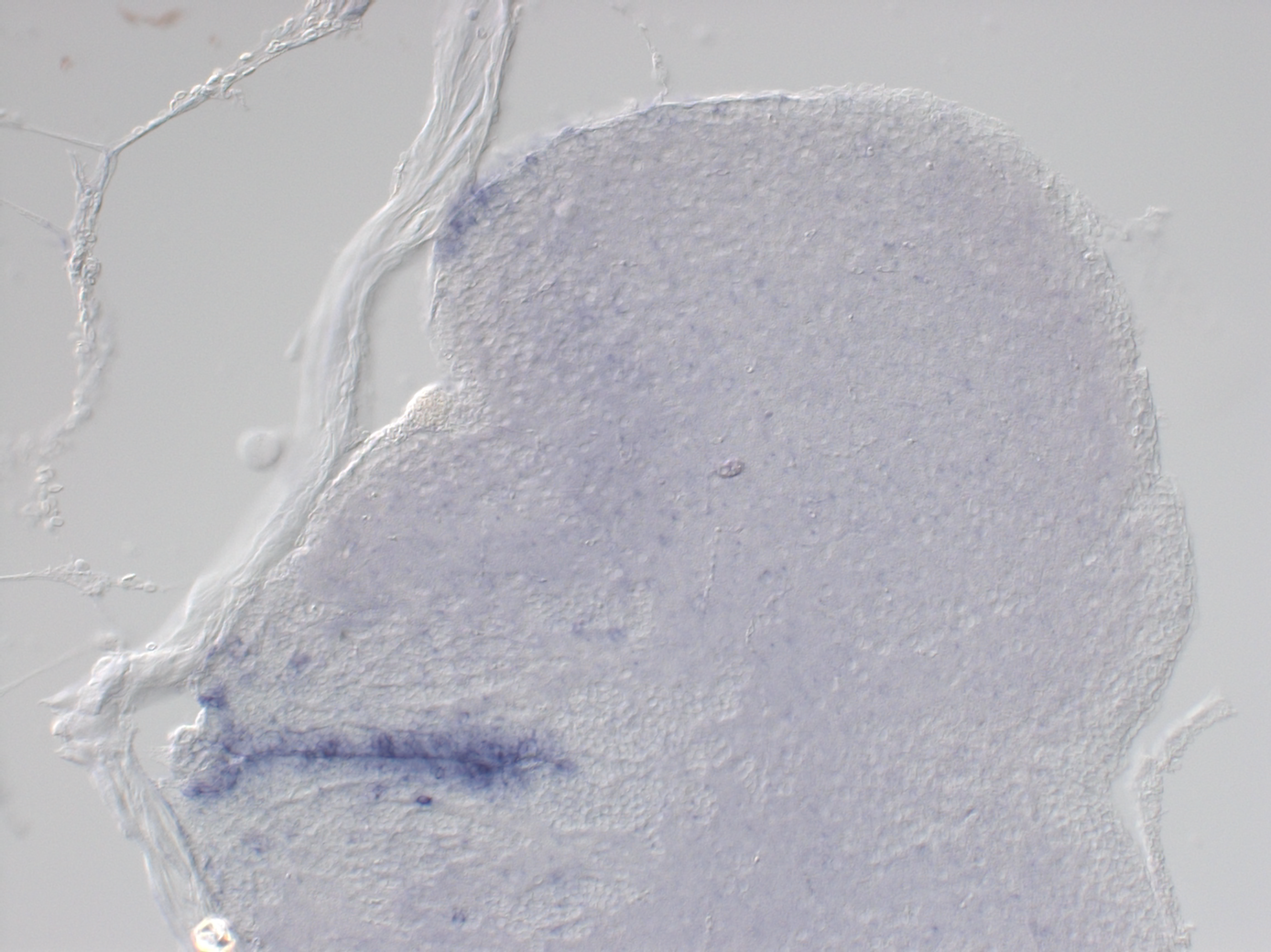

Supplement: Gene expression analysis in the adult zebrafish pallium — Dataset 1 Expression of eomesb in the embryonic brain and the adult pallium in zebrafish. Raw data of Figure S2 and additional image files of eomesb expression in the embryo and the adult pallium. Dataset 2 Images of negative control. No signal was detected in the absence of the riboprobe, demonstrating that the antibody reacts specifically with the synthetic RNA. Dataset 3 Expression of eomesa in the zebrafish pallium. Raw data of Figure 1 and additional image files of eomesa expression in the adult pallium. Dataset 4 Expression of emx1, emx2 and emx3 in the zebrafish larval brain. Raw data of Figure S3 and additional image files of emx gene expression in the zebrafish larvae. Dataset 5 Expression of emx1, emx2 and emx3 in the zebrafish pallium. Raw data of Figure 2 and additional image files of emx gene expression in the adult pallium. Dataset 6 Expression of Prox1 in the zebrafish pallium. Raw data of Figure 3 and additional image files of Prox1 expression in the adult pallium. Dataset 7 Expression of ascl1a in the zebrafish pallium. Raw data of Figure 4 and additional image files of ascl1a expression in the adult pallium. [file f1000research-3-7777-s0000.tgz › ascl1a_1.TIF]

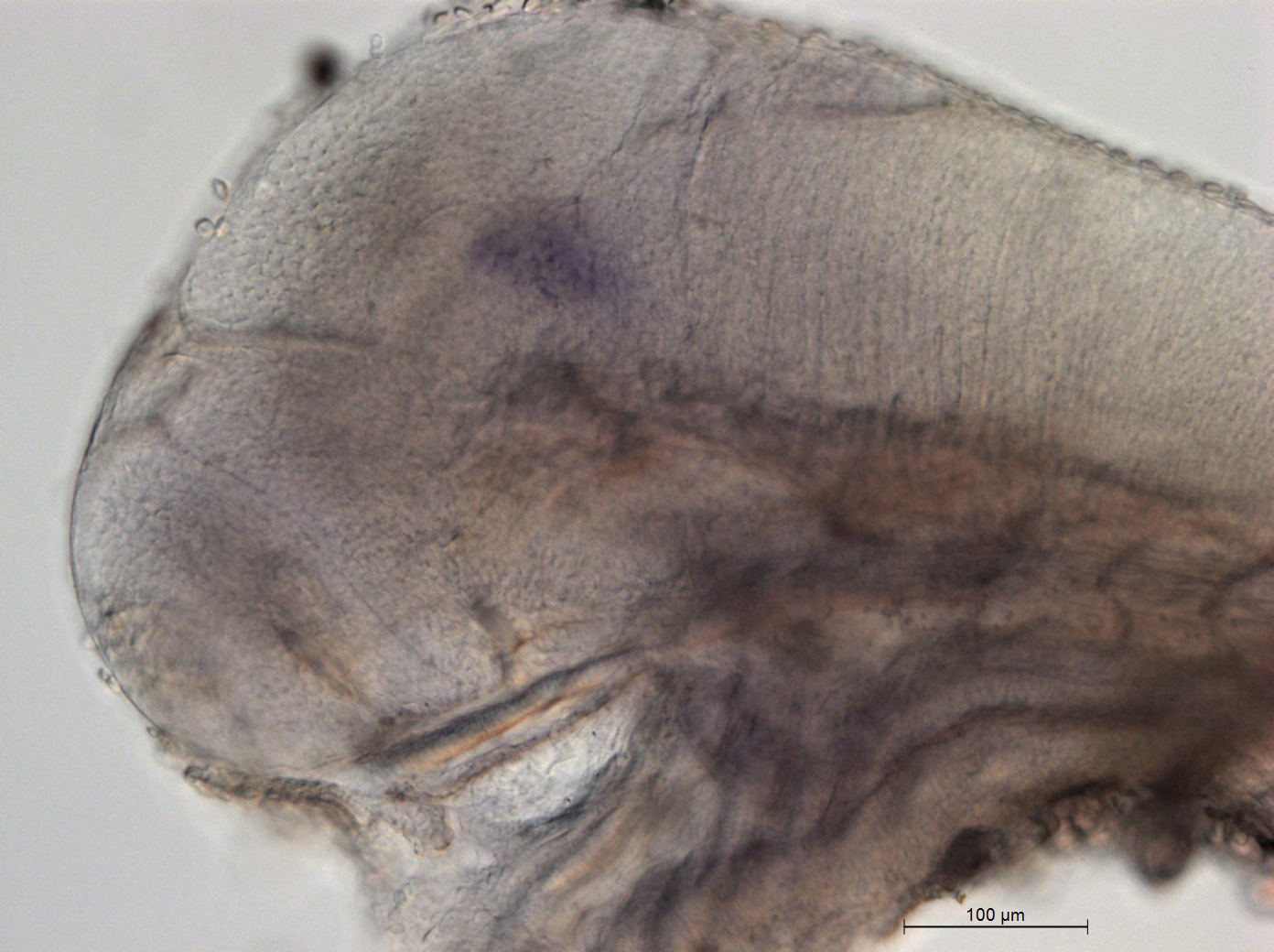

Supplement: Gene expression analysis in the adult zebrafish pallium — Dataset 1 Expression of eomesb in the embryonic brain and the adult pallium in zebrafish. Raw data of Figure S2 and additional image files of eomesb expression in the embryo and the adult pallium. Dataset 2 Images of negative control. No signal was detected in the absence of the riboprobe, demonstrating that the antibody reacts specifically with the synthetic RNA. Dataset 3 Expression of eomesa in the zebrafish pallium. Raw data of Figure 1 and additional image files of eomesa expression in the adult pallium. Dataset 4 Expression of emx1, emx2 and emx3 in the zebrafish larval brain. Raw data of Figure S3 and additional image files of emx gene expression in the zebrafish larvae. Dataset 5 Expression of emx1, emx2 and emx3 in the zebrafish pallium. Raw data of Figure 2 and additional image files of emx gene expression in the adult pallium. Dataset 6 Expression of Prox1 in the zebrafish pallium. Raw data of Figure 3 and additional image files of Prox1 expression in the adult pallium. Dataset 7 Expression of ascl1a in the zebrafish pallium. Raw data of Figure 4 and additional image files of ascl1a expression in the adult pallium. [file f1000research-3-7777-s0000.tgz › FigS2_Ganzetal.tif]

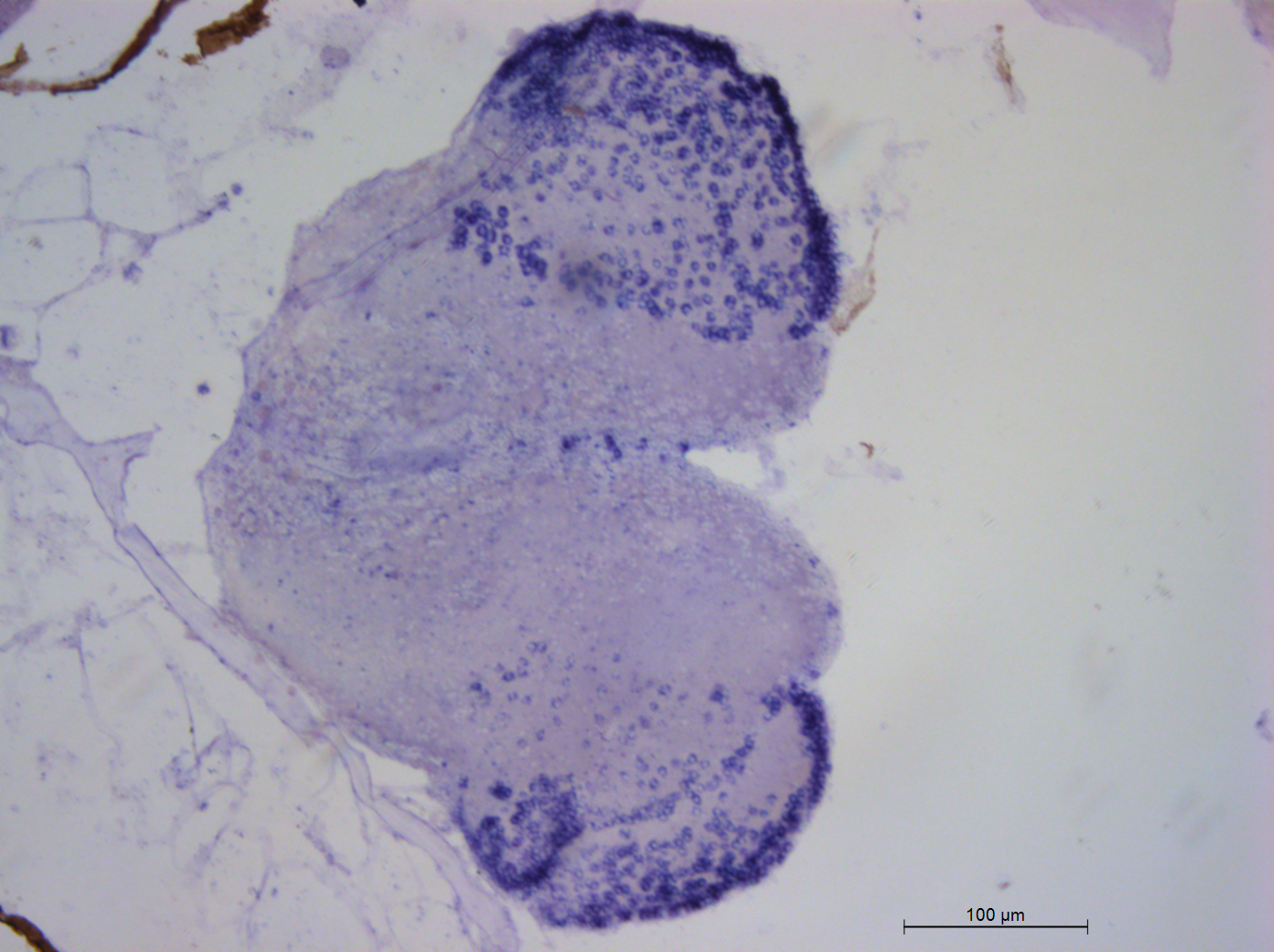

Supplement: Gene expression analysis in the adult zebrafish pallium — Dataset 1 Expression of eomesb in the embryonic brain and the adult pallium in zebrafish. Raw data of Figure S2 and additional image files of eomesb expression in the embryo and the adult pallium. Dataset 2 Images of negative control. No signal was detected in the absence of the riboprobe, demonstrating that the antibody reacts specifically with the synthetic RNA. Dataset 3 Expression of eomesa in the zebrafish pallium. Raw data of Figure 1 and additional image files of eomesa expression in the adult pallium. Dataset 4 Expression of emx1, emx2 and emx3 in the zebrafish larval brain. Raw data of Figure S3 and additional image files of emx gene expression in the zebrafish larvae. Dataset 5 Expression of emx1, emx2 and emx3 in the zebrafish pallium. Raw data of Figure 2 and additional image files of emx gene expression in the adult pallium. Dataset 6 Expression of Prox1 in the zebrafish pallium. Raw data of Figure 3 and additional image files of Prox1 expression in the adult pallium. Dataset 7 Expression of ascl1a in the zebrafish pallium. Raw data of Figure 4 and additional image files of ascl1a expression in the adult pallium. [file f1000research-3-7777-s0000.tgz › Fig1B_Ganzetal.tif]

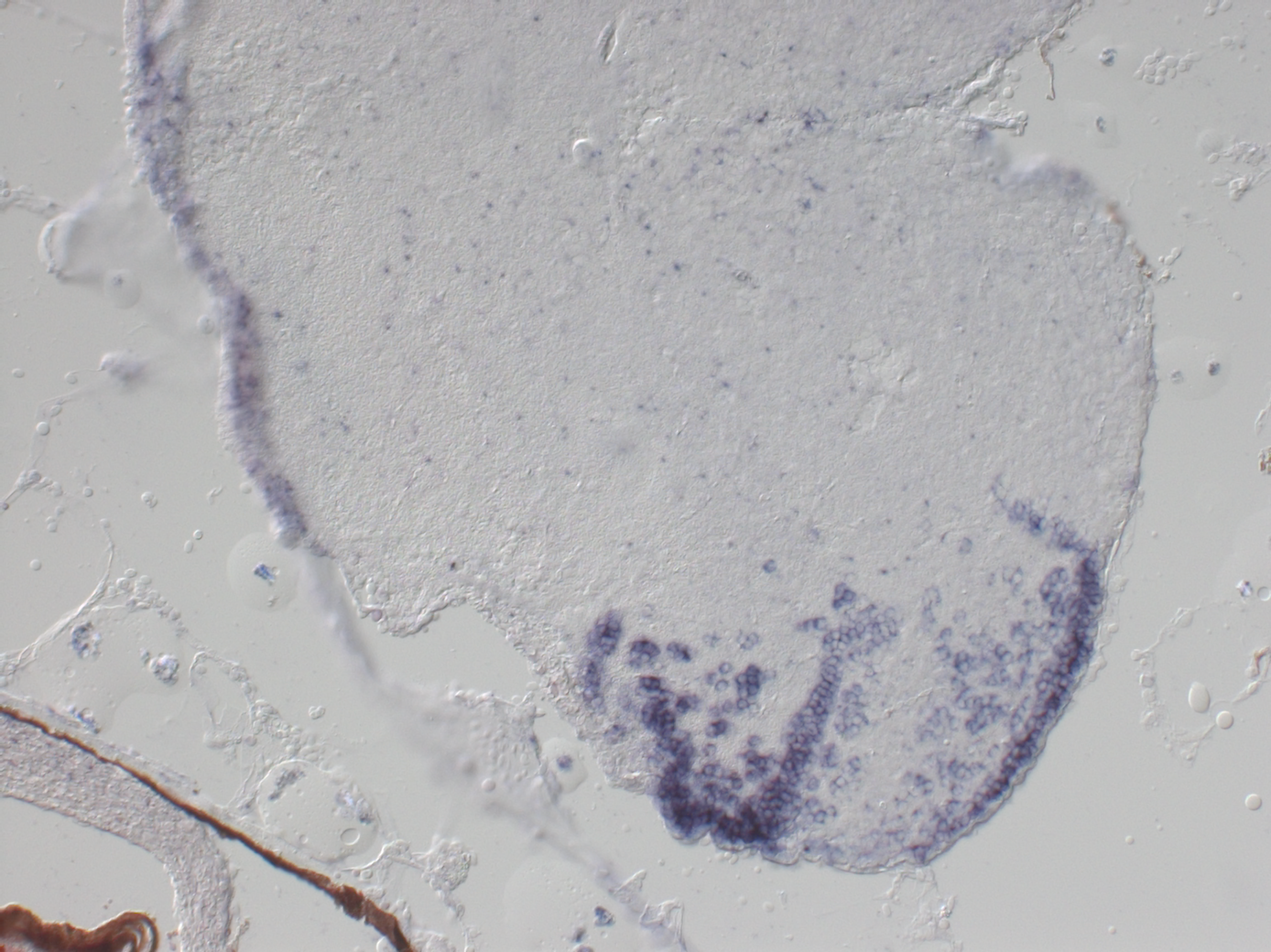

Supplement: Gene expression analysis in the adult zebrafish pallium — Dataset 1 Expression of eomesb in the embryonic brain and the adult pallium in zebrafish. Raw data of Figure S2 and additional image files of eomesb expression in the embryo and the adult pallium. Dataset 2 Images of negative control. No signal was detected in the absence of the riboprobe, demonstrating that the antibody reacts specifically with the synthetic RNA. Dataset 3 Expression of eomesa in the zebrafish pallium. Raw data of Figure 1 and additional image files of eomesa expression in the adult pallium. Dataset 4 Expression of emx1, emx2 and emx3 in the zebrafish larval brain. Raw data of Figure S3 and additional image files of emx gene expression in the zebrafish larvae. Dataset 5 Expression of emx1, emx2 and emx3 in the zebrafish pallium. Raw data of Figure 2 and additional image files of emx gene expression in the adult pallium. Dataset 6 Expression of Prox1 in the zebrafish pallium. Raw data of Figure 3 and additional image files of Prox1 expression in the adult pallium. Dataset 7 Expression of ascl1a in the zebrafish pallium. Raw data of Figure 4 and additional image files of ascl1a expression in the adult pallium. [file f1000research-3-7777-s0000.tgz › Fig1C_Ganzetal.tif]

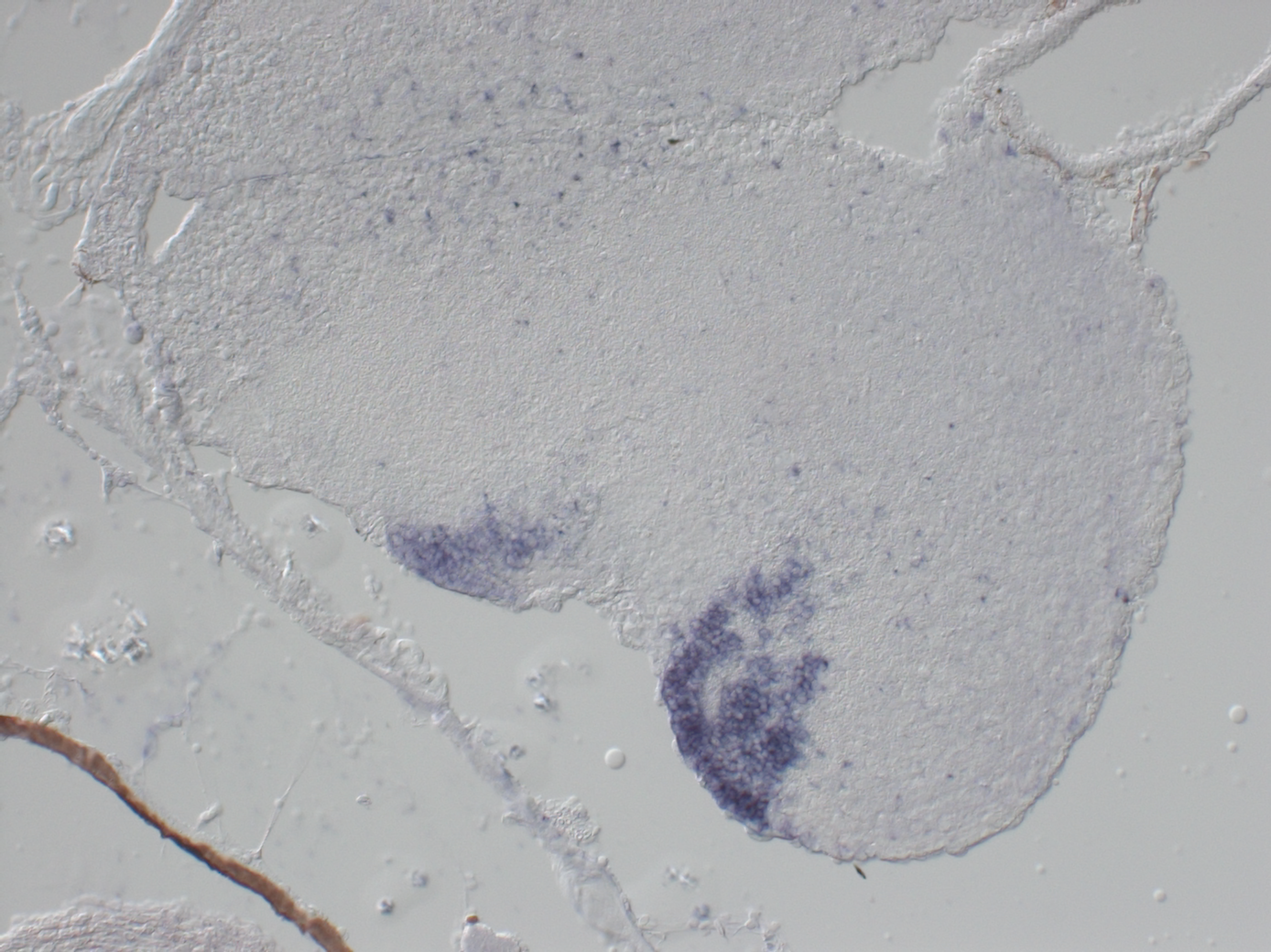

Supplement: Gene expression analysis in the adult zebrafish pallium — Dataset 1 Expression of eomesb in the embryonic brain and the adult pallium in zebrafish. Raw data of Figure S2 and additional image files of eomesb expression in the embryo and the adult pallium. Dataset 2 Images of negative control. No signal was detected in the absence of the riboprobe, demonstrating that the antibody reacts specifically with the synthetic RNA. Dataset 3 Expression of eomesa in the zebrafish pallium. Raw data of Figure 1 and additional image files of eomesa expression in the adult pallium. Dataset 4 Expression of emx1, emx2 and emx3 in the zebrafish larval brain. Raw data of Figure S3 and additional image files of emx gene expression in the zebrafish larvae. Dataset 5 Expression of emx1, emx2 and emx3 in the zebrafish pallium. Raw data of Figure 2 and additional image files of emx gene expression in the adult pallium. Dataset 6 Expression of Prox1 in the zebrafish pallium. Raw data of Figure 3 and additional image files of Prox1 expression in the adult pallium. Dataset 7 Expression of ascl1a in the zebrafish pallium. Raw data of Figure 4 and additional image files of ascl1a expression in the adult pallium. [file f1000research-3-7777-s0000.tgz › Fig1D_Ganzetal.tif]

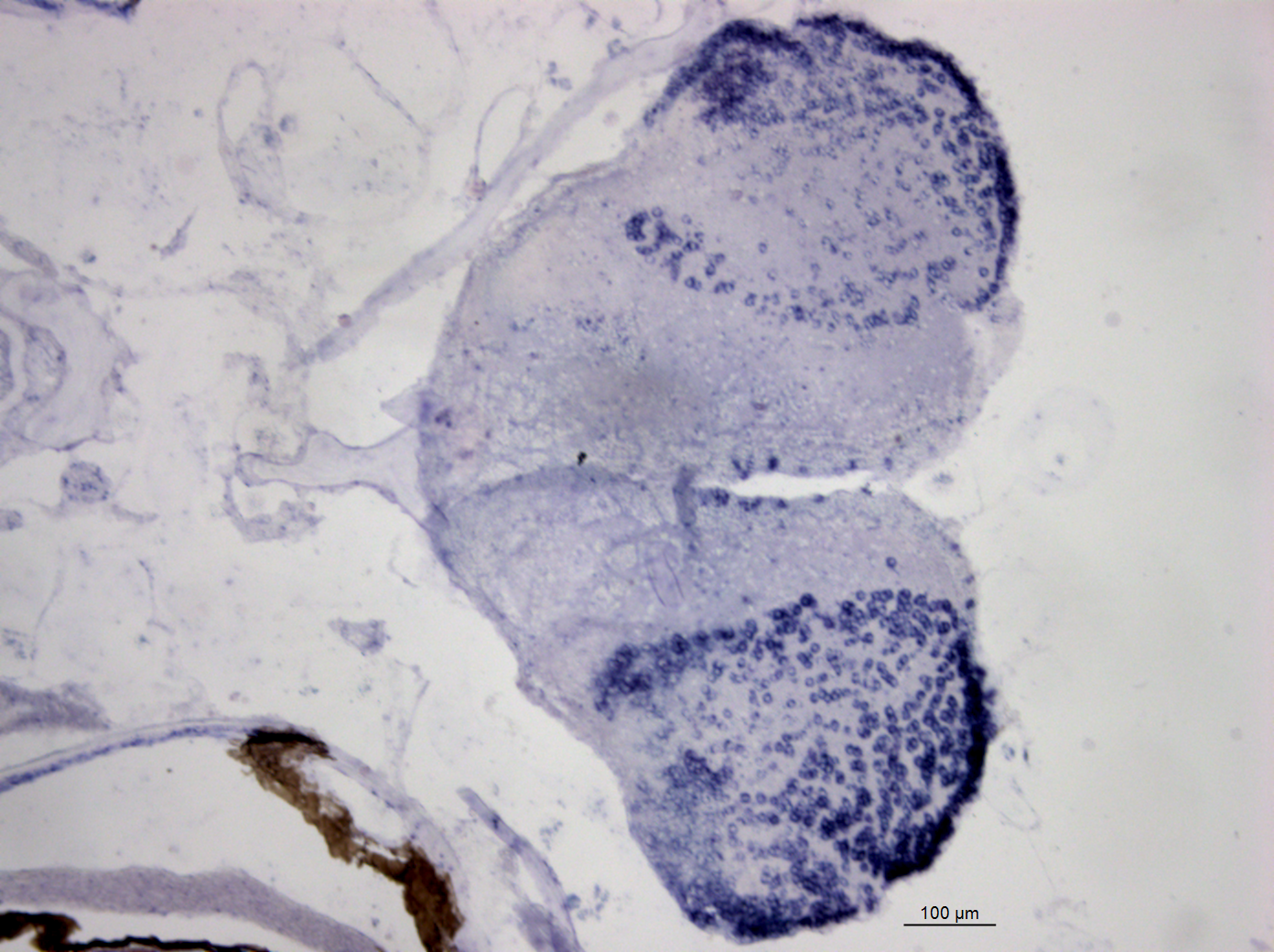

Supplement: Gene expression analysis in the adult zebrafish pallium — Dataset 1 Expression of eomesb in the embryonic brain and the adult pallium in zebrafish. Raw data of Figure S2 and additional image files of eomesb expression in the embryo and the adult pallium. Dataset 2 Images of negative control. No signal was detected in the absence of the riboprobe, demonstrating that the antibody reacts specifically with the synthetic RNA. Dataset 3 Expression of eomesa in the zebrafish pallium. Raw data of Figure 1 and additional image files of eomesa expression in the adult pallium. Dataset 4 Expression of emx1, emx2 and emx3 in the zebrafish larval brain. Raw data of Figure S3 and additional image files of emx gene expression in the zebrafish larvae. Dataset 5 Expression of emx1, emx2 and emx3 in the zebrafish pallium. Raw data of Figure 2 and additional image files of emx gene expression in the adult pallium. Dataset 6 Expression of Prox1 in the zebrafish pallium. Raw data of Figure 3 and additional image files of Prox1 expression in the adult pallium. Dataset 7 Expression of ascl1a in the zebrafish pallium. Raw data of Figure 4 and additional image files of ascl1a expression in the adult pallium. [file f1000research-3-7777-s0000.tgz › Fig1A_Ganzetal.tif]

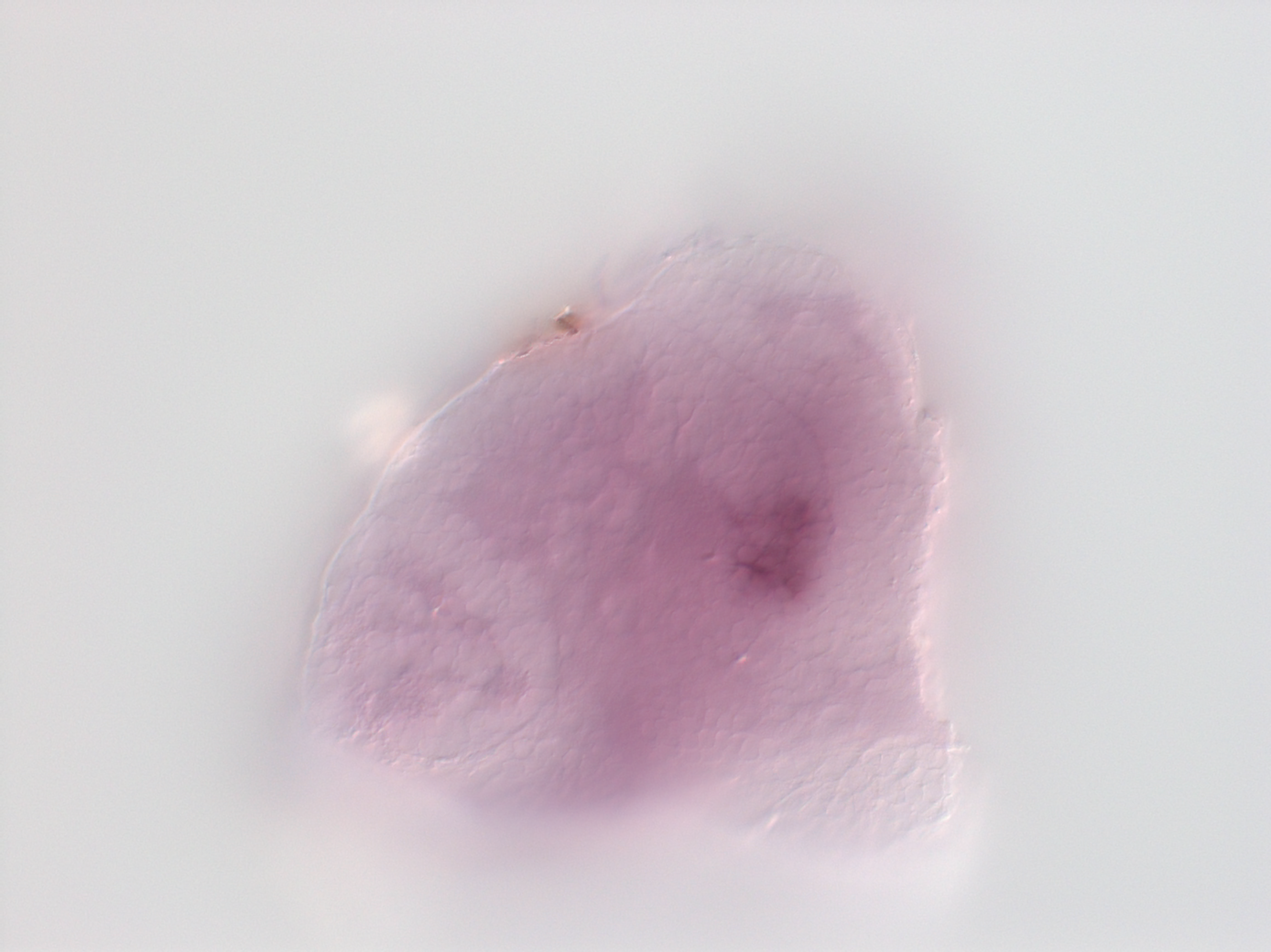

Supplement: Gene expression analysis in the adult zebrafish pallium — Dataset 1 Expression of eomesb in the embryonic brain and the adult pallium in zebrafish. Raw data of Figure S2 and additional image files of eomesb expression in the embryo and the adult pallium. Dataset 2 Images of negative control. No signal was detected in the absence of the riboprobe, demonstrating that the antibody reacts specifically with the synthetic RNA. Dataset 3 Expression of eomesa in the zebrafish pallium. Raw data of Figure 1 and additional image files of eomesa expression in the adult pallium. Dataset 4 Expression of emx1, emx2 and emx3 in the zebrafish larval brain. Raw data of Figure S3 and additional image files of emx gene expression in the zebrafish larvae. Dataset 5 Expression of emx1, emx2 and emx3 in the zebrafish pallium. Raw data of Figure 2 and additional image files of emx gene expression in the adult pallium. Dataset 6 Expression of Prox1 in the zebrafish pallium. Raw data of Figure 3 and additional image files of Prox1 expression in the adult pallium. Dataset 7 Expression of ascl1a in the zebrafish pallium. Raw data of Figure 4 and additional image files of ascl1a expression in the adult pallium. [file f1000research-3-7777-s0000.tgz › FigS3B_Ganzetal.TIF]

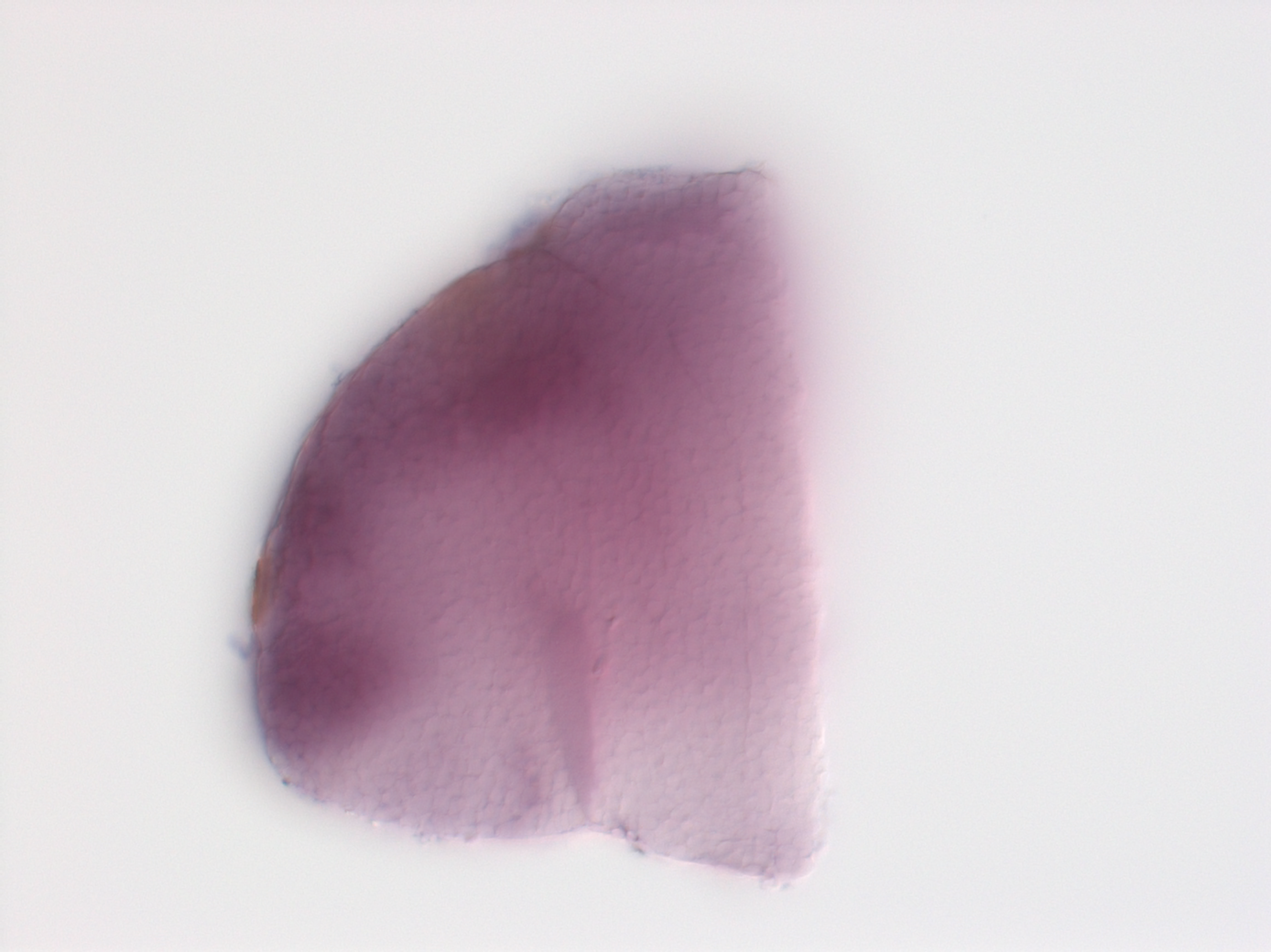

Supplement: Gene expression analysis in the adult zebrafish pallium — Dataset 1 Expression of eomesb in the embryonic brain and the adult pallium in zebrafish. Raw data of Figure S2 and additional image files of eomesb expression in the embryo and the adult pallium. Dataset 2 Images of negative control. No signal was detected in the absence of the riboprobe, demonstrating that the antibody reacts specifically with the synthetic RNA. Dataset 3 Expression of eomesa in the zebrafish pallium. Raw data of Figure 1 and additional image files of eomesa expression in the adult pallium. Dataset 4 Expression of emx1, emx2 and emx3 in the zebrafish larval brain. Raw data of Figure S3 and additional image files of emx gene expression in the zebrafish larvae. Dataset 5 Expression of emx1, emx2 and emx3 in the zebrafish pallium. Raw data of Figure 2 and additional image files of emx gene expression in the adult pallium. Dataset 6 Expression of Prox1 in the zebrafish pallium. Raw data of Figure 3 and additional image files of Prox1 expression in the adult pallium. Dataset 7 Expression of ascl1a in the zebrafish pallium. Raw data of Figure 4 and additional image files of ascl1a expression in the adult pallium. [file f1000research-3-7777-s0000.tgz › FigS3C_Ganzetal.TIF]

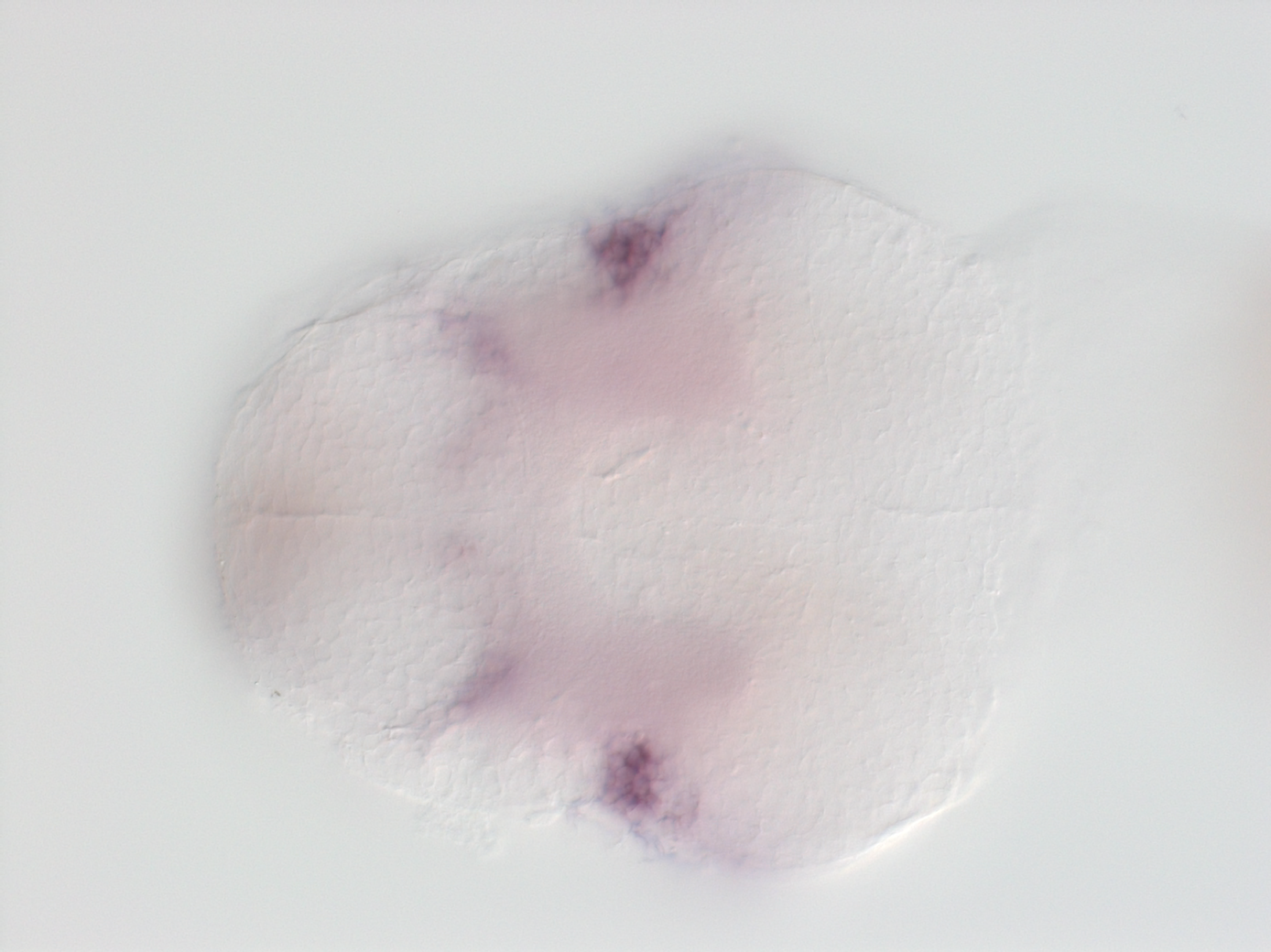

Supplement: Gene expression analysis in the adult zebrafish pallium — Dataset 1 Expression of eomesb in the embryonic brain and the adult pallium in zebrafish. Raw data of Figure S2 and additional image files of eomesb expression in the embryo and the adult pallium. Dataset 2 Images of negative control. No signal was detected in the absence of the riboprobe, demonstrating that the antibody reacts specifically with the synthetic RNA. Dataset 3 Expression of eomesa in the zebrafish pallium. Raw data of Figure 1 and additional image files of eomesa expression in the adult pallium. Dataset 4 Expression of emx1, emx2 and emx3 in the zebrafish larval brain. Raw data of Figure S3 and additional image files of emx gene expression in the zebrafish larvae. Dataset 5 Expression of emx1, emx2 and emx3 in the zebrafish pallium. Raw data of Figure 2 and additional image files of emx gene expression in the adult pallium. Dataset 6 Expression of Prox1 in the zebrafish pallium. Raw data of Figure 3 and additional image files of Prox1 expression in the adult pallium. Dataset 7 Expression of ascl1a in the zebrafish pallium. Raw data of Figure 4 and additional image files of ascl1a expression in the adult pallium. [file f1000research-3-7777-s0000.tgz › FigS3A_Ganzetal.TIF]

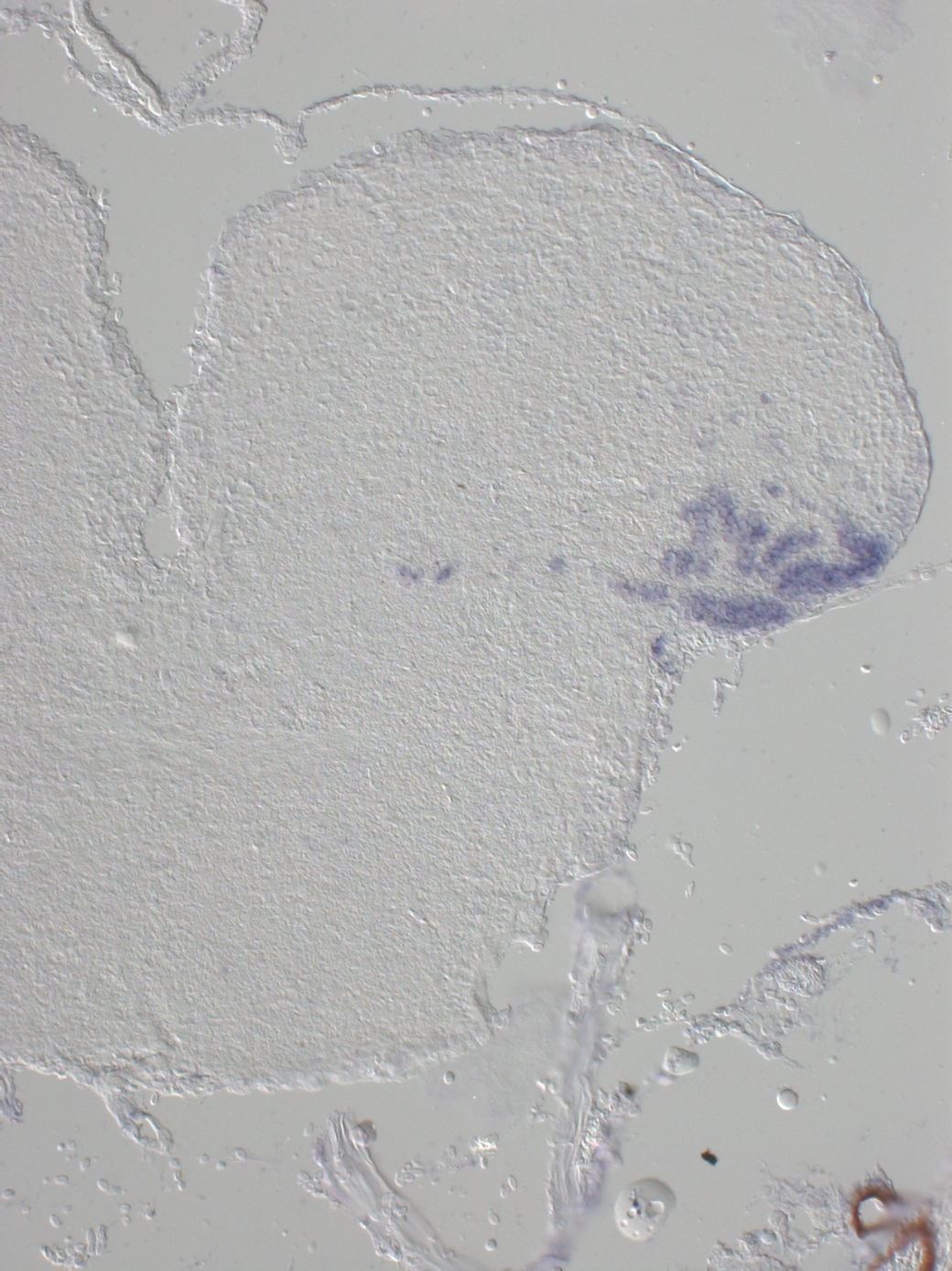

Supplement: Gene expression analysis in the adult zebrafish pallium — Dataset 1 Expression of eomesb in the embryonic brain and the adult pallium in zebrafish. Raw data of Figure S2 and additional image files of eomesb expression in the embryo and the adult pallium. Dataset 2 Images of negative control. No signal was detected in the absence of the riboprobe, demonstrating that the antibody reacts specifically with the synthetic RNA. Dataset 3 Expression of eomesa in the zebrafish pallium. Raw data of Figure 1 and additional image files of eomesa expression in the adult pallium. Dataset 4 Expression of emx1, emx2 and emx3 in the zebrafish larval brain. Raw data of Figure S3 and additional image files of emx gene expression in the zebrafish larvae. Dataset 5 Expression of emx1, emx2 and emx3 in the zebrafish pallium. Raw data of Figure 2 and additional image files of emx gene expression in the adult pallium. Dataset 6 Expression of Prox1 in the zebrafish pallium. Raw data of Figure 3 and additional image files of Prox1 expression in the adult pallium. Dataset 7 Expression of ascl1a in the zebrafish pallium. Raw data of Figure 4 and additional image files of ascl1a expression in the adult pallium. [file f1000research-3-7777-s0000.tgz › Fig2A_Ganzetal.TIF]

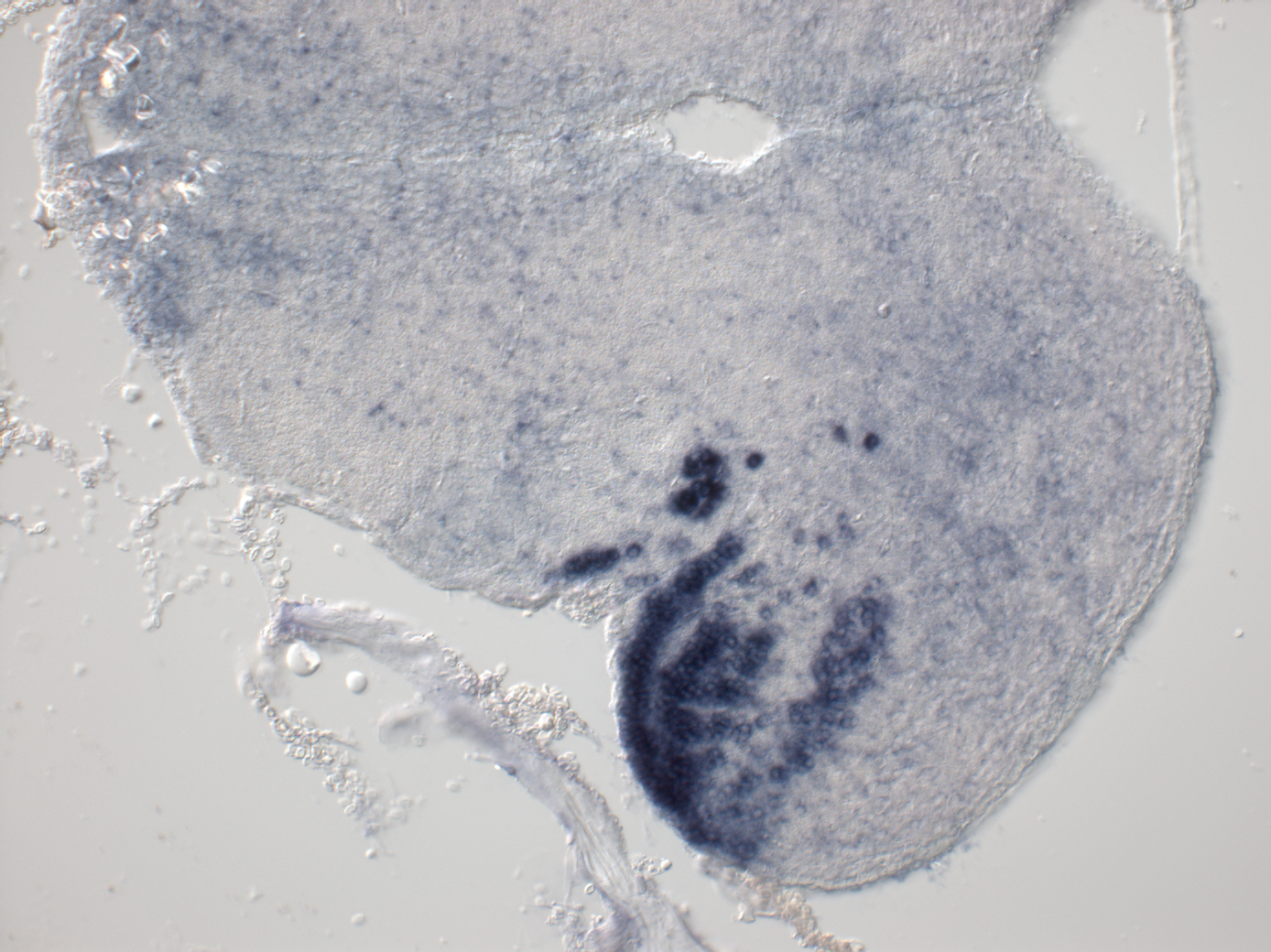

Supplement: Gene expression analysis in the adult zebrafish pallium — Dataset 1 Expression of eomesb in the embryonic brain and the adult pallium in zebrafish. Raw data of Figure S2 and additional image files of eomesb expression in the embryo and the adult pallium. Dataset 2 Images of negative control. No signal was detected in the absence of the riboprobe, demonstrating that the antibody reacts specifically with the synthetic RNA. Dataset 3 Expression of eomesa in the zebrafish pallium. Raw data of Figure 1 and additional image files of eomesa expression in the adult pallium. Dataset 4 Expression of emx1, emx2 and emx3 in the zebrafish larval brain. Raw data of Figure S3 and additional image files of emx gene expression in the zebrafish larvae. Dataset 5 Expression of emx1, emx2 and emx3 in the zebrafish pallium. Raw data of Figure 2 and additional image files of emx gene expression in the adult pallium. Dataset 6 Expression of Prox1 in the zebrafish pallium. Raw data of Figure 3 and additional image files of Prox1 expression in the adult pallium. Dataset 7 Expression of ascl1a in the zebrafish pallium. Raw data of Figure 4 and additional image files of ascl1a expression in the adult pallium. [file f1000research-3-7777-s0000.tgz › Fig2B_Ganzetal.TIF]

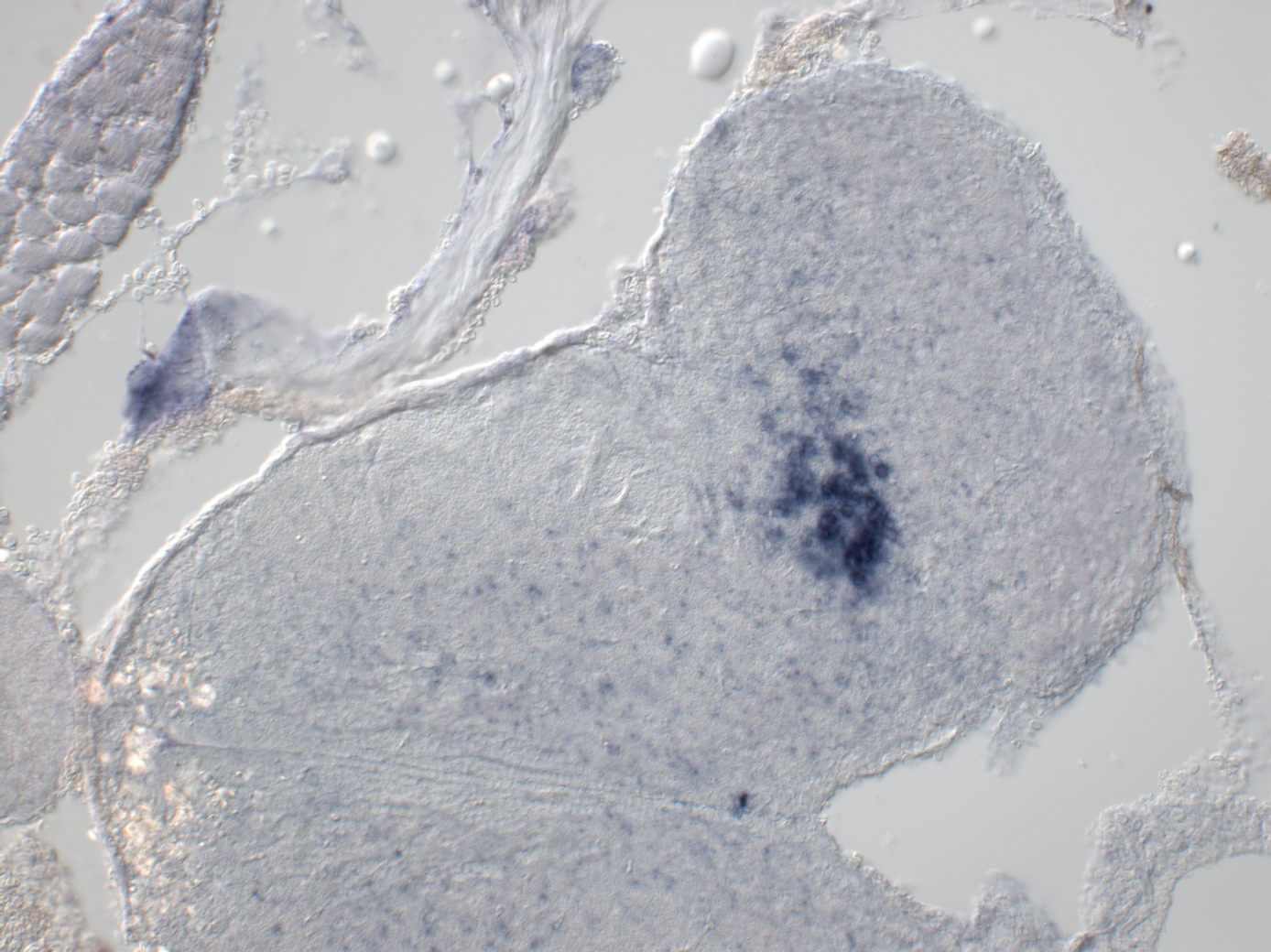

Supplement: Gene expression analysis in the adult zebrafish pallium — Dataset 1 Expression of eomesb in the embryonic brain and the adult pallium in zebrafish. Raw data of Figure S2 and additional image files of eomesb expression in the embryo and the adult pallium. Dataset 2 Images of negative control. No signal was detected in the absence of the riboprobe, demonstrating that the antibody reacts specifically with the synthetic RNA. Dataset 3 Expression of eomesa in the zebrafish pallium. Raw data of Figure 1 and additional image files of eomesa expression in the adult pallium. Dataset 4 Expression of emx1, emx2 and emx3 in the zebrafish larval brain. Raw data of Figure S3 and additional image files of emx gene expression in the zebrafish larvae. Dataset 5 Expression of emx1, emx2 and emx3 in the zebrafish pallium. Raw data of Figure 2 and additional image files of emx gene expression in the adult pallium. Dataset 6 Expression of Prox1 in the zebrafish pallium. Raw data of Figure 3 and additional image files of Prox1 expression in the adult pallium. Dataset 7 Expression of ascl1a in the zebrafish pallium. Raw data of Figure 4 and additional image files of ascl1a expression in the adult pallium. [file f1000research-3-7777-s0000.tgz › Fig2C_Ganzetal.TIF]

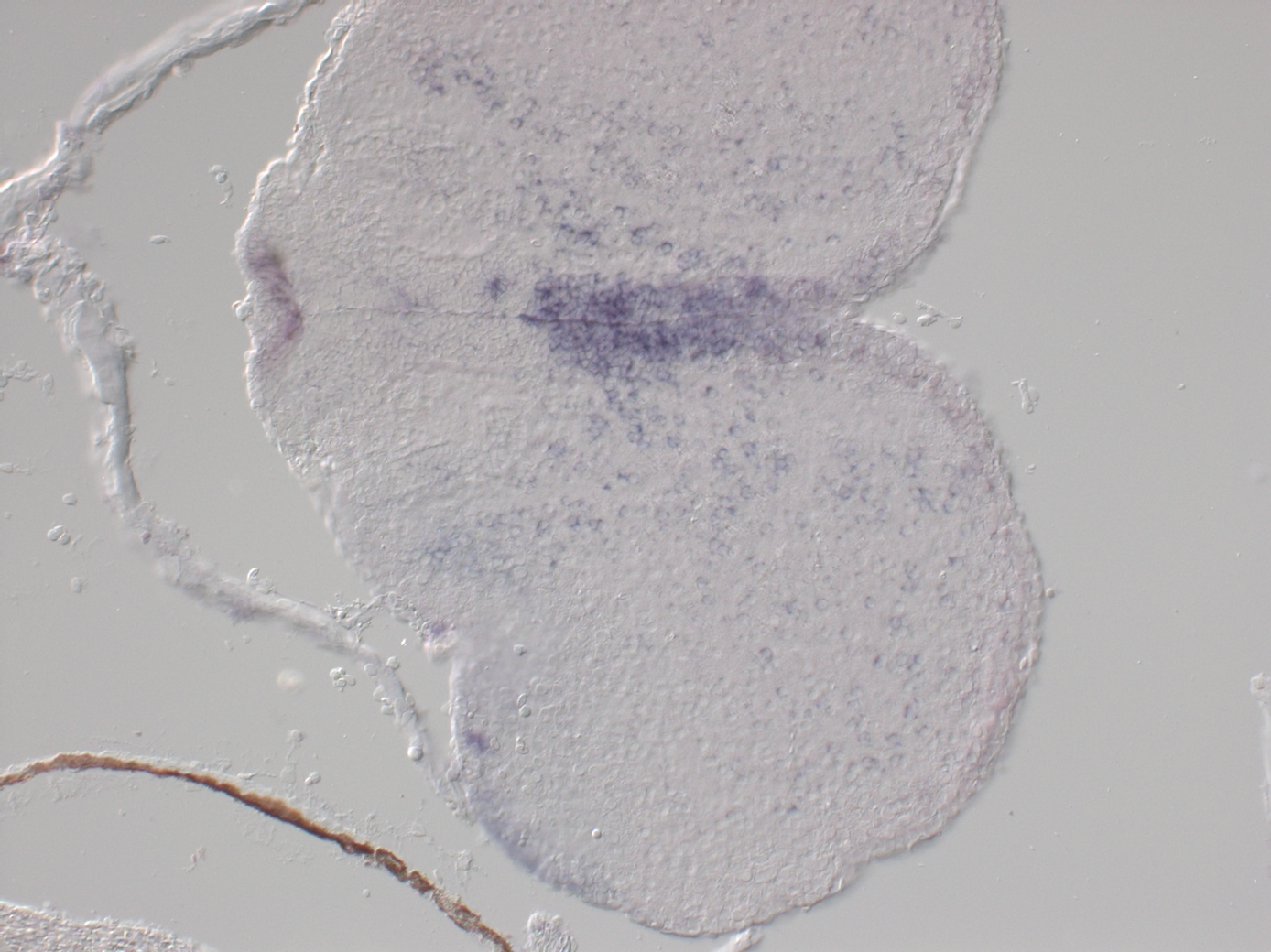

Supplement: Gene expression analysis in the adult zebrafish pallium — Dataset 1 Expression of eomesb in the embryonic brain and the adult pallium in zebrafish. Raw data of Figure S2 and additional image files of eomesb expression in the embryo and the adult pallium. Dataset 2 Images of negative control. No signal was detected in the absence of the riboprobe, demonstrating that the antibody reacts specifically with the synthetic RNA. Dataset 3 Expression of eomesa in the zebrafish pallium. Raw data of Figure 1 and additional image files of eomesa expression in the adult pallium. Dataset 4 Expression of emx1, emx2 and emx3 in the zebrafish larval brain. Raw data of Figure S3 and additional image files of emx gene expression in the zebrafish larvae. Dataset 5 Expression of emx1, emx2 and emx3 in the zebrafish pallium. Raw data of Figure 2 and additional image files of emx gene expression in the adult pallium. Dataset 6 Expression of Prox1 in the zebrafish pallium. Raw data of Figure 3 and additional image files of Prox1 expression in the adult pallium. Dataset 7 Expression of ascl1a in the zebrafish pallium. Raw data of Figure 4 and additional image files of ascl1a expression in the adult pallium. [file f1000research-3-7777-s0000.tgz › Fig2G_Ganzetal.TIF]

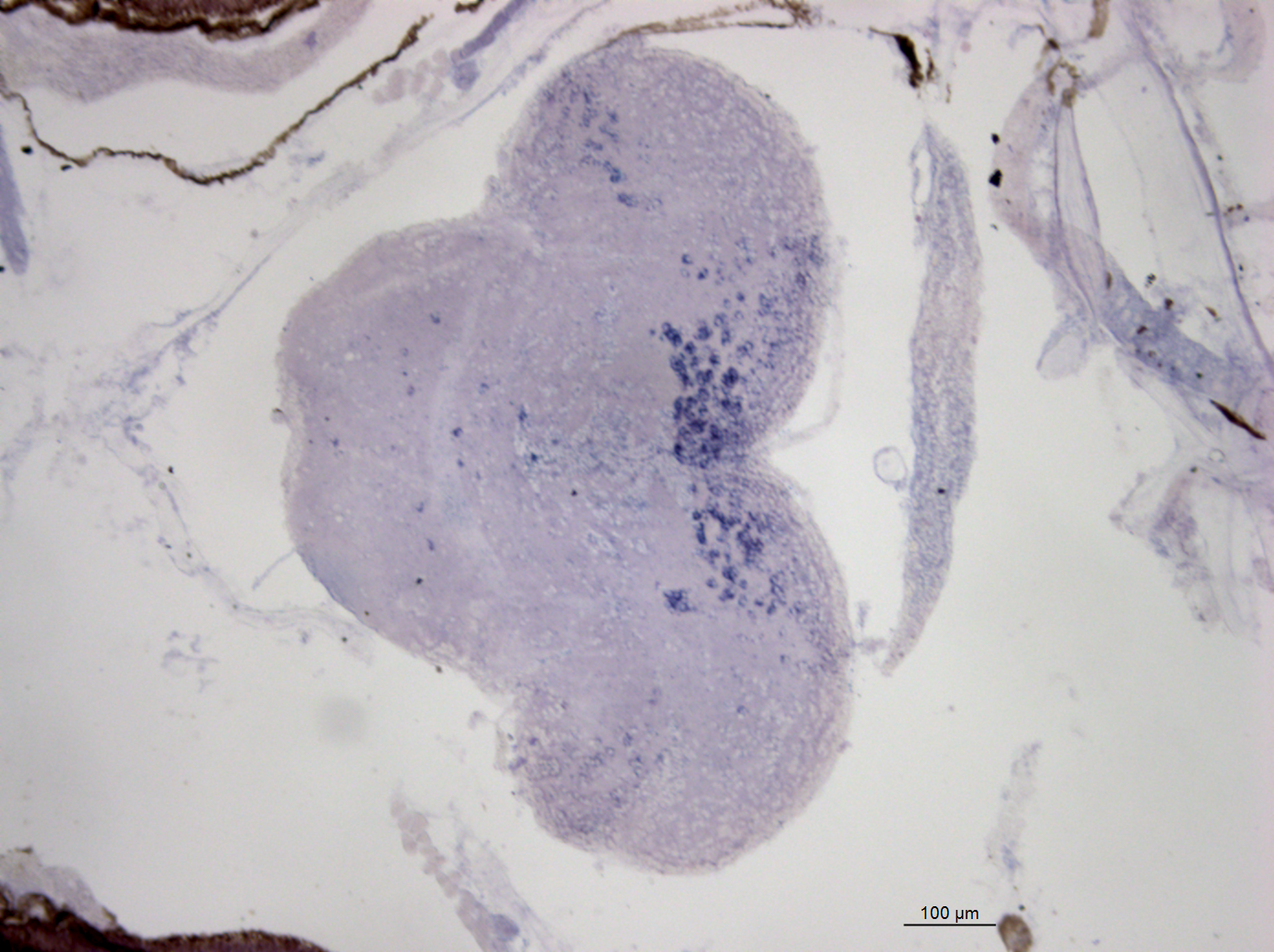

Supplement: Gene expression analysis in the adult zebrafish pallium — Dataset 1 Expression of eomesb in the embryonic brain and the adult pallium in zebrafish. Raw data of Figure S2 and additional image files of eomesb expression in the embryo and the adult pallium. Dataset 2 Images of negative control. No signal was detected in the absence of the riboprobe, demonstrating that the antibody reacts specifically with the synthetic RNA. Dataset 3 Expression of eomesa in the zebrafish pallium. Raw data of Figure 1 and additional image files of eomesa expression in the adult pallium. Dataset 4 Expression of emx1, emx2 and emx3 in the zebrafish larval brain. Raw data of Figure S3 and additional image files of emx gene expression in the zebrafish larvae. Dataset 5 Expression of emx1, emx2 and emx3 in the zebrafish pallium. Raw data of Figure 2 and additional image files of emx gene expression in the adult pallium. Dataset 6 Expression of Prox1 in the zebrafish pallium. Raw data of Figure 3 and additional image files of Prox1 expression in the adult pallium. Dataset 7 Expression of ascl1a in the zebrafish pallium. Raw data of Figure 4 and additional image files of ascl1a expression in the adult pallium. [file f1000research-3-7777-s0000.tgz › Fig2I_Ganzetal.tif]

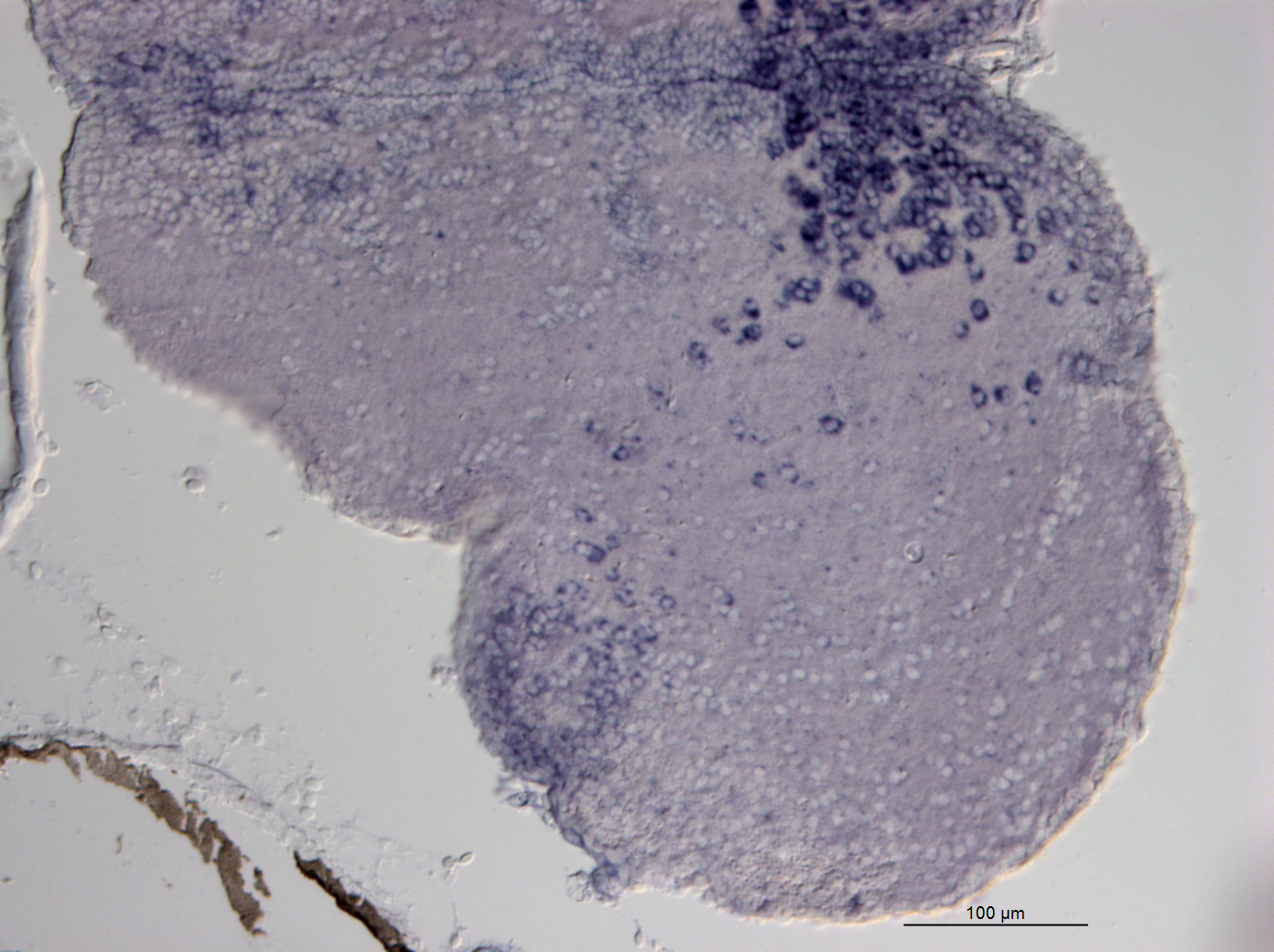

Supplement: Gene expression analysis in the adult zebrafish pallium — Dataset 1 Expression of eomesb in the embryonic brain and the adult pallium in zebrafish. Raw data of Figure S2 and additional image files of eomesb expression in the embryo and the adult pallium. Dataset 2 Images of negative control. No signal was detected in the absence of the riboprobe, demonstrating that the antibody reacts specifically with the synthetic RNA. Dataset 3 Expression of eomesa in the zebrafish pallium. Raw data of Figure 1 and additional image files of eomesa expression in the adult pallium. Dataset 4 Expression of emx1, emx2 and emx3 in the zebrafish larval brain. Raw data of Figure S3 and additional image files of emx gene expression in the zebrafish larvae. Dataset 5 Expression of emx1, emx2 and emx3 in the zebrafish pallium. Raw data of Figure 2 and additional image files of emx gene expression in the adult pallium. Dataset 6 Expression of Prox1 in the zebrafish pallium. Raw data of Figure 3 and additional image files of Prox1 expression in the adult pallium. Dataset 7 Expression of ascl1a in the zebrafish pallium. Raw data of Figure 4 and additional image files of ascl1a expression in the adult pallium. [file f1000research-3-7777-s0000.tgz › Fig2H_Ganzetal.tif]

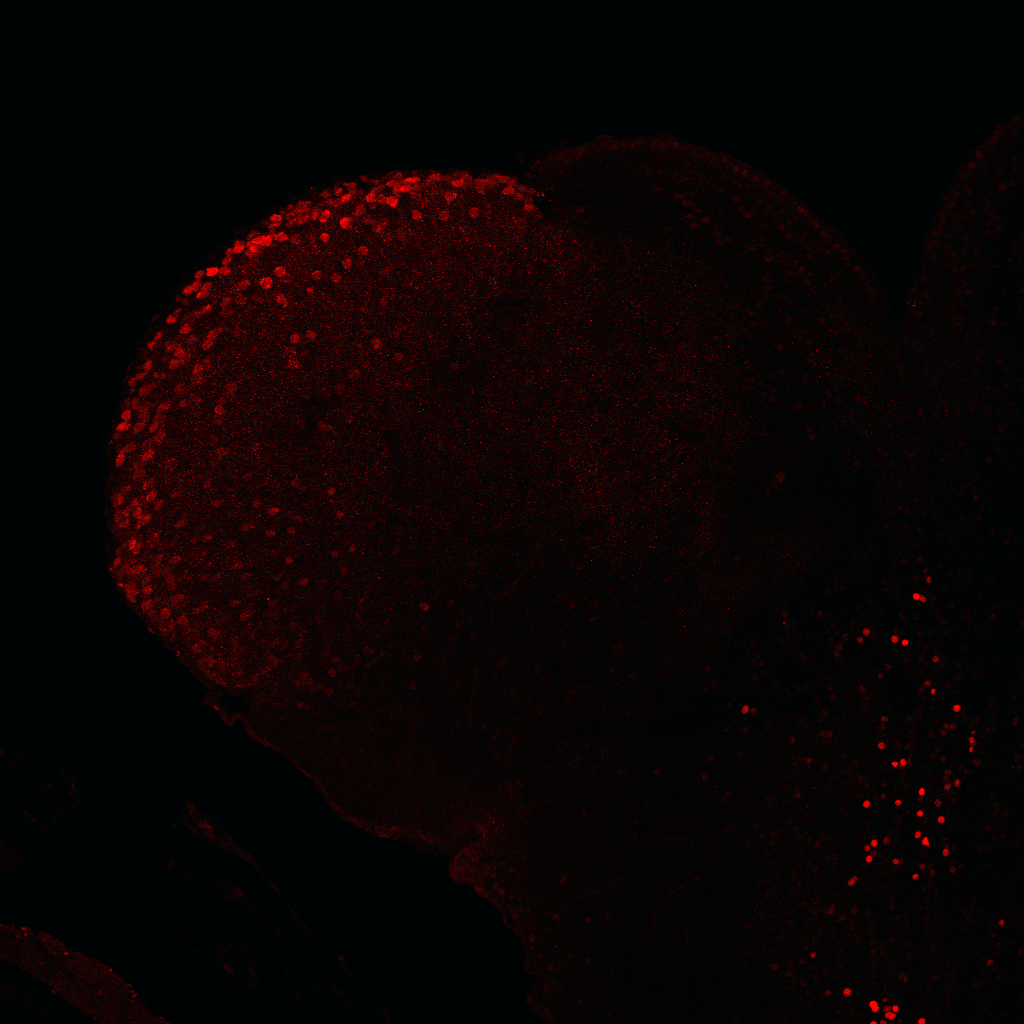

Supplement: Gene expression analysis in the adult zebrafish pallium — Dataset 1 Expression of eomesb in the embryonic brain and the adult pallium in zebrafish. Raw data of Figure S2 and additional image files of eomesb expression in the embryo and the adult pallium. Dataset 2 Images of negative control. No signal was detected in the absence of the riboprobe, demonstrating that the antibody reacts specifically with the synthetic RNA. Dataset 3 Expression of eomesa in the zebrafish pallium. Raw data of Figure 1 and additional image files of eomesa expression in the adult pallium. Dataset 4 Expression of emx1, emx2 and emx3 in the zebrafish larval brain. Raw data of Figure S3 and additional image files of emx gene expression in the zebrafish larvae. Dataset 5 Expression of emx1, emx2 and emx3 in the zebrafish pallium. Raw data of Figure 2 and additional image files of emx gene expression in the adult pallium. Dataset 6 Expression of Prox1 in the zebrafish pallium. Raw data of Figure 3 and additional image files of Prox1 expression in the adult pallium. Dataset 7 Expression of ascl1a in the zebrafish pallium. Raw data of Figure 4 and additional image files of ascl1a expression in the adult pallium. [file f1000research-3-7777-s0000.tgz › Fig_3A_Ganzetal.tif]

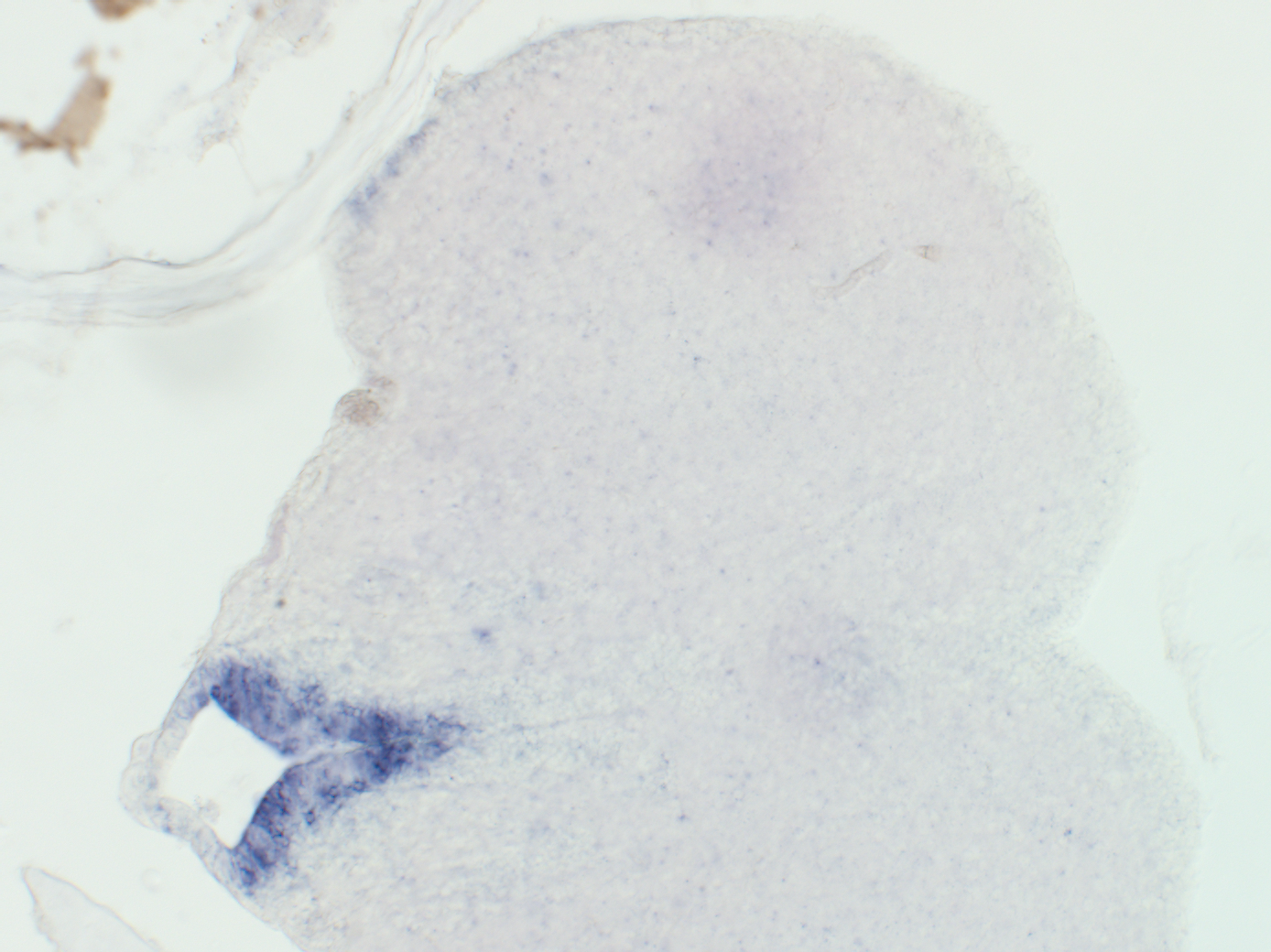

Supplement: Gene expression analysis in the adult zebrafish pallium — Dataset 1 Expression of eomesb in the embryonic brain and the adult pallium in zebrafish. Raw data of Figure S2 and additional image files of eomesb expression in the embryo and the adult pallium. Dataset 2 Images of negative control. No signal was detected in the absence of the riboprobe, demonstrating that the antibody reacts specifically with the synthetic RNA. Dataset 3 Expression of eomesa in the zebrafish pallium. Raw data of Figure 1 and additional image files of eomesa expression in the adult pallium. Dataset 4 Expression of emx1, emx2 and emx3 in the zebrafish larval brain. Raw data of Figure S3 and additional image files of emx gene expression in the zebrafish larvae. Dataset 5 Expression of emx1, emx2 and emx3 in the zebrafish pallium. Raw data of Figure 2 and additional image files of emx gene expression in the adult pallium. Dataset 6 Expression of Prox1 in the zebrafish pallium. Raw data of Figure 3 and additional image files of Prox1 expression in the adult pallium. Dataset 7 Expression of ascl1a in the zebrafish pallium. Raw data of Figure 4 and additional image files of ascl1a expression in the adult pallium. [file f1000research-3-7777-s0000.tgz › Fig4B_Ganzetal.TIF]

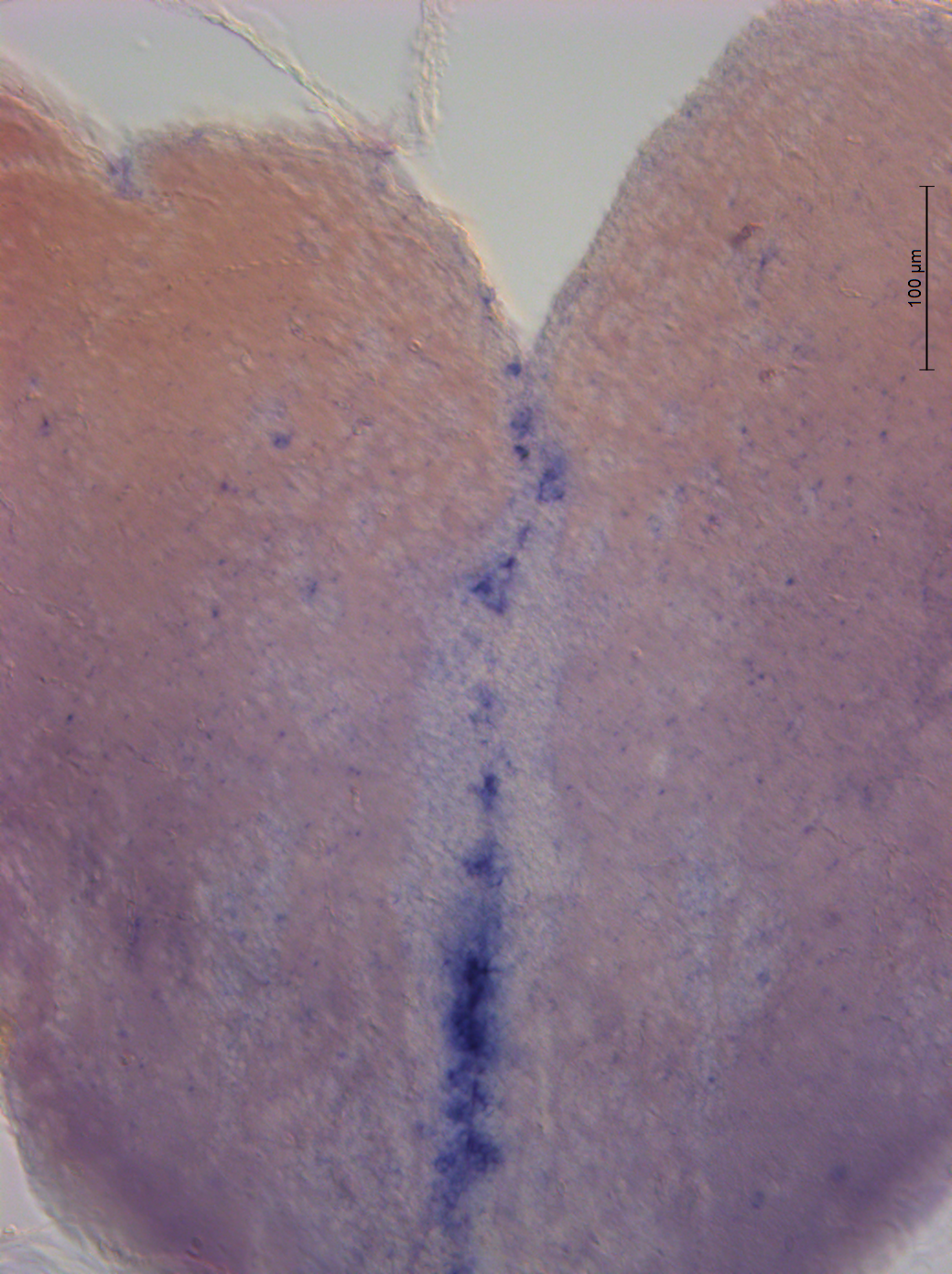

Supplement: Gene expression analysis in the adult zebrafish pallium — Dataset 1 Expression of eomesb in the embryonic brain and the adult pallium in zebrafish. Raw data of Figure S2 and additional image files of eomesb expression in the embryo and the adult pallium. Dataset 2 Images of negative control. No signal was detected in the absence of the riboprobe, demonstrating that the antibody reacts specifically with the synthetic RNA. Dataset 3 Expression of eomesa in the zebrafish pallium. Raw data of Figure 1 and additional image files of eomesa expression in the adult pallium. Dataset 4 Expression of emx1, emx2 and emx3 in the zebrafish larval brain. Raw data of Figure S3 and additional image files of emx gene expression in the zebrafish larvae. Dataset 5 Expression of emx1, emx2 and emx3 in the zebrafish pallium. Raw data of Figure 2 and additional image files of emx gene expression in the adult pallium. Dataset 6 Expression of Prox1 in the zebrafish pallium. Raw data of Figure 3 and additional image files of Prox1 expression in the adult pallium. Dataset 7 Expression of ascl1a in the zebrafish pallium. Raw data of Figure 4 and additional image files of ascl1a expression in the adult pallium. [file f1000research-3-7777-s0000.tgz › Fig4C_Ganzetal.tif]

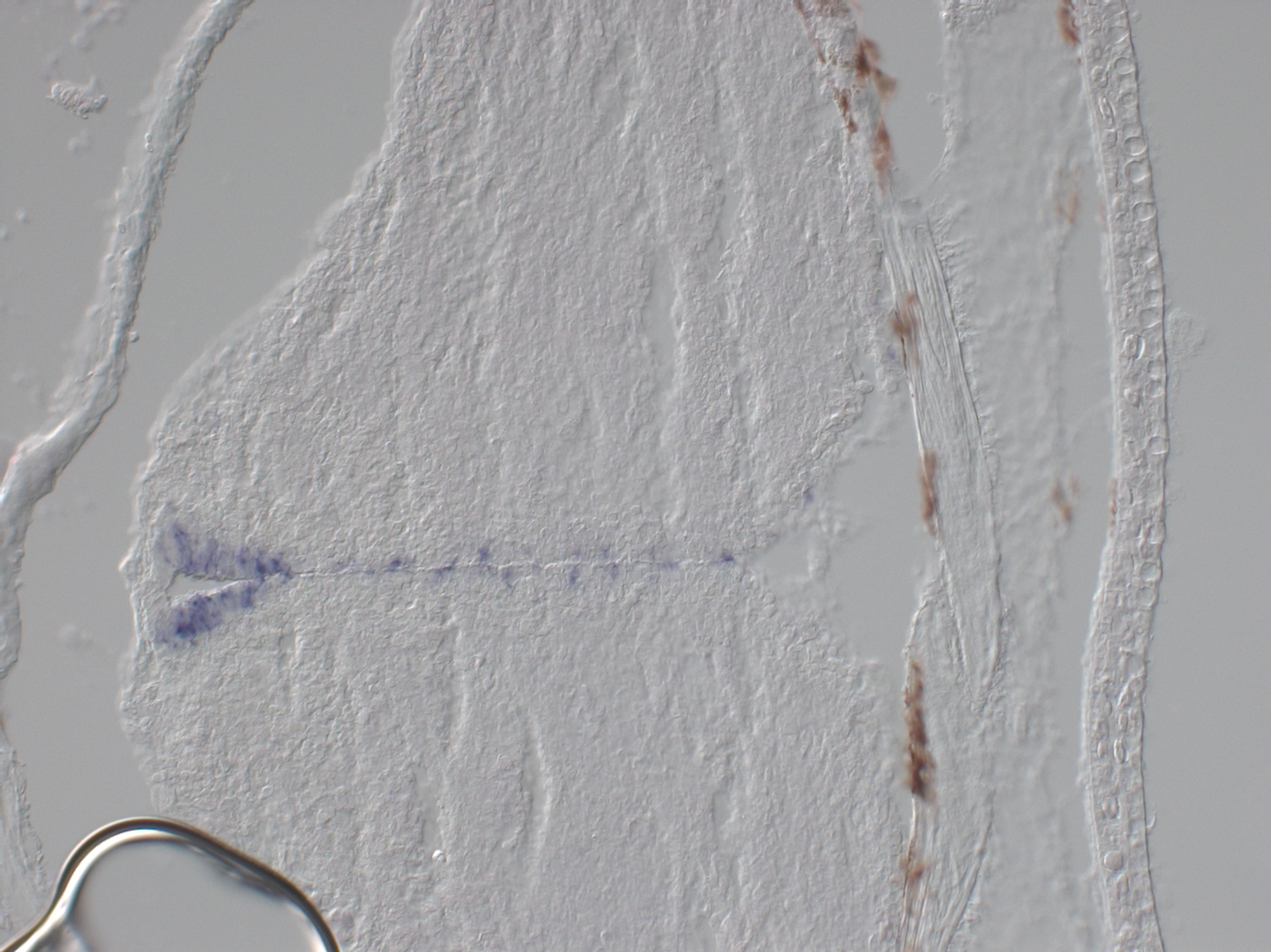

Supplement: Gene expression analysis in the adult zebrafish pallium — Dataset 1 Expression of eomesb in the embryonic brain and the adult pallium in zebrafish. Raw data of Figure S2 and additional image files of eomesb expression in the embryo and the adult pallium. Dataset 2 Images of negative control. No signal was detected in the absence of the riboprobe, demonstrating that the antibody reacts specifically with the synthetic RNA. Dataset 3 Expression of eomesa in the zebrafish pallium. Raw data of Figure 1 and additional image files of eomesa expression in the adult pallium. Dataset 4 Expression of emx1, emx2 and emx3 in the zebrafish larval brain. Raw data of Figure S3 and additional image files of emx gene expression in the zebrafish larvae. Dataset 5 Expression of emx1, emx2 and emx3 in the zebrafish pallium. Raw data of Figure 2 and additional image files of emx gene expression in the adult pallium. Dataset 6 Expression of Prox1 in the zebrafish pallium. Raw data of Figure 3 and additional image files of Prox1 expression in the adult pallium. Dataset 7 Expression of ascl1a in the zebrafish pallium. Raw data of Figure 4 and additional image files of ascl1a expression in the adult pallium. [file f1000research-3-7777-s0000.tgz › Fig4A_Ganzetal.TIF]

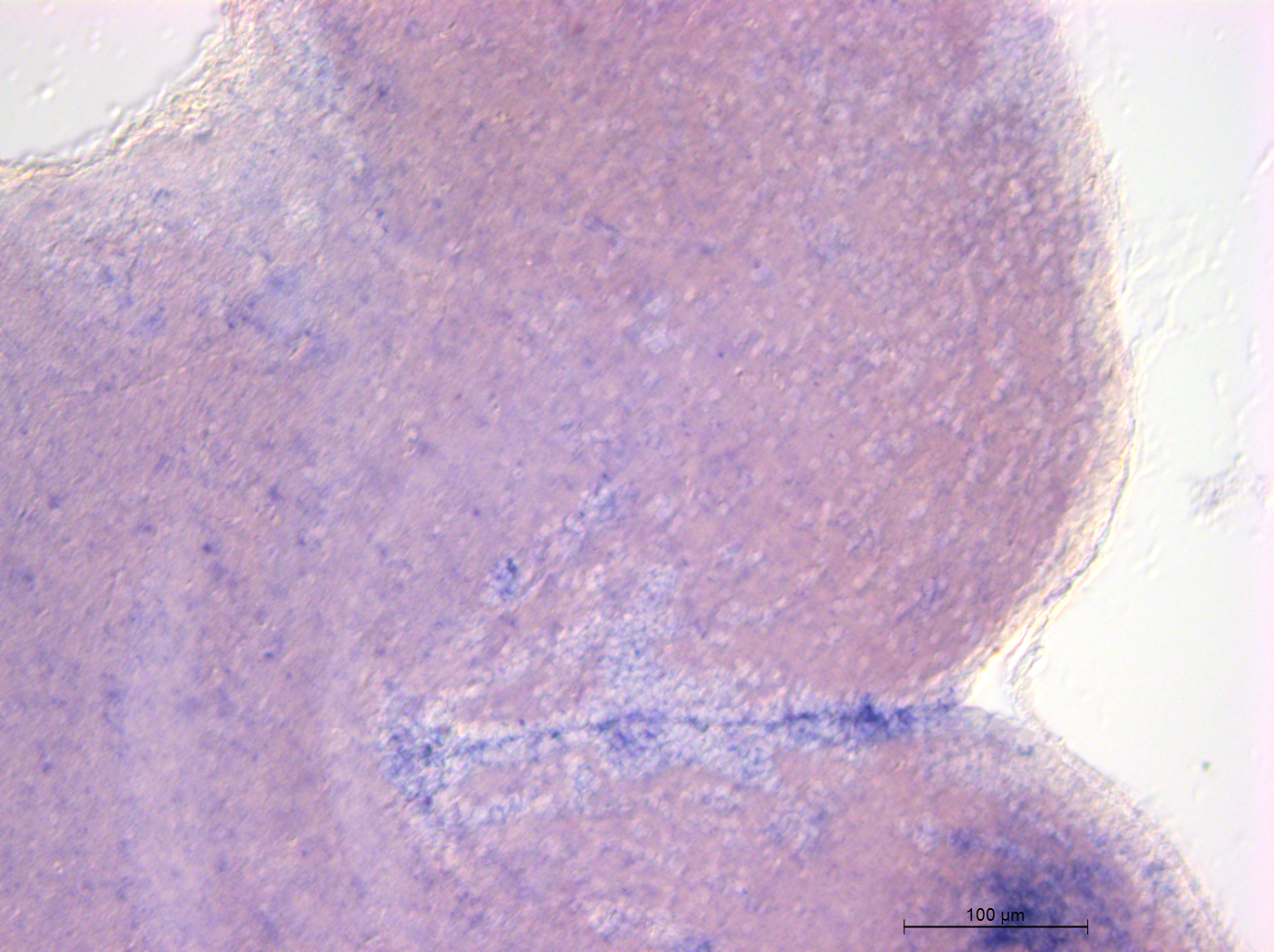

Supplement: Gene expression analysis in the adult zebrafish pallium — Dataset 1 Expression of eomesb in the embryonic brain and the adult pallium in zebrafish. Raw data of Figure S2 and additional image files of eomesb expression in the embryo and the adult pallium. Dataset 2 Images of negative control. No signal was detected in the absence of the riboprobe, demonstrating that the antibody reacts specifically with the synthetic RNA. Dataset 3 Expression of eomesa in the zebrafish pallium. Raw data of Figure 1 and additional image files of eomesa expression in the adult pallium. Dataset 4 Expression of emx1, emx2 and emx3 in the zebrafish larval brain. Raw data of Figure S3 and additional image files of emx gene expression in the zebrafish larvae. Dataset 5 Expression of emx1, emx2 and emx3 in the zebrafish pallium. Raw data of Figure 2 and additional image files of emx gene expression in the adult pallium. Dataset 6 Expression of Prox1 in the zebrafish pallium. Raw data of Figure 3 and additional image files of Prox1 expression in the adult pallium. Dataset 7 Expression of ascl1a in the zebrafish pallium. Raw data of Figure 4 and additional image files of ascl1a expression in the adult pallium. [file f1000research-3-7777-s0000.tgz › Fig4E_Ganzetal.tif]

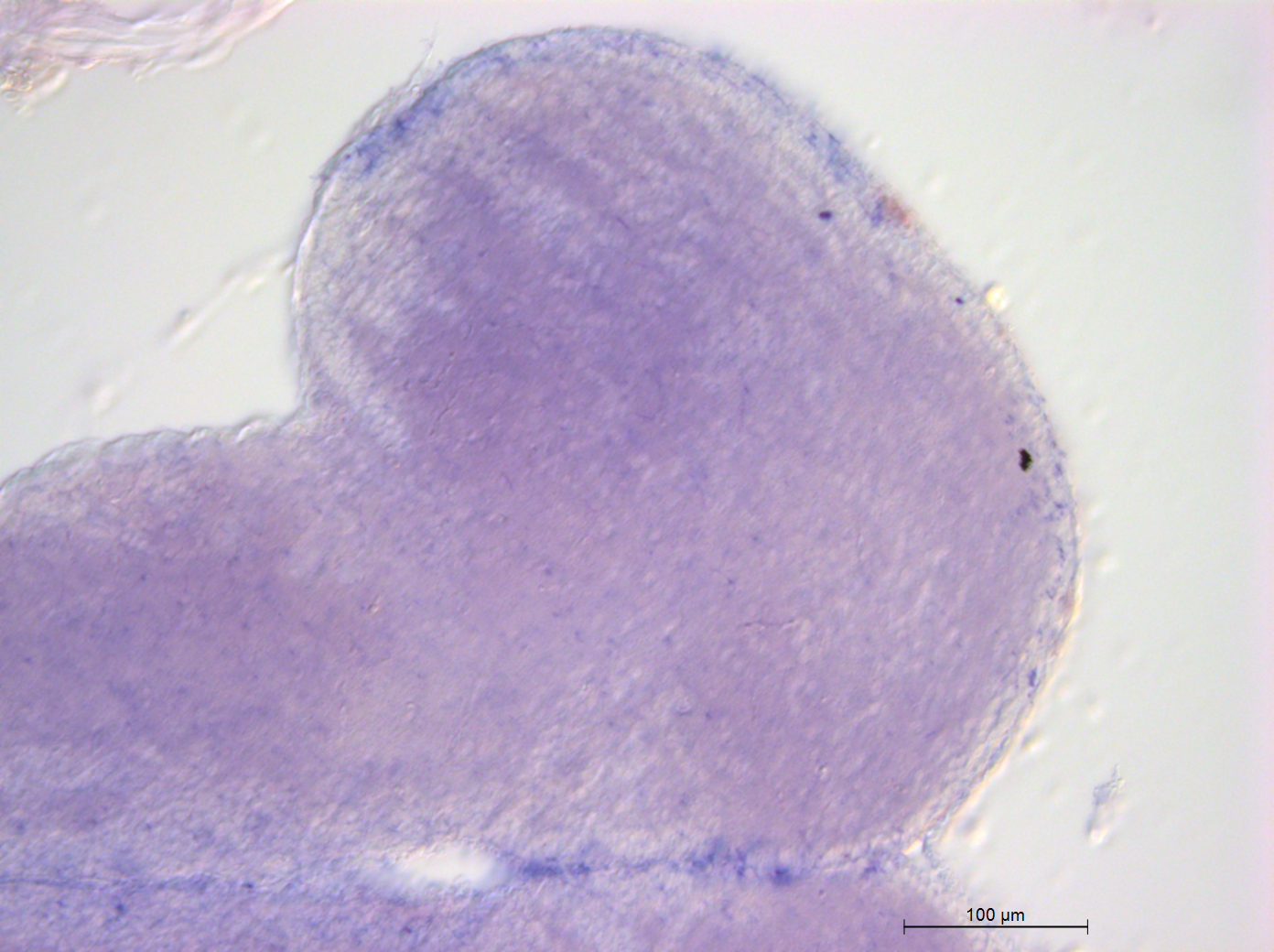

Supplement: Gene expression analysis in the adult zebrafish pallium — Dataset 1 Expression of eomesb in the embryonic brain and the adult pallium in zebrafish. Raw data of Figure S2 and additional image files of eomesb expression in the embryo and the adult pallium. Dataset 2 Images of negative control. No signal was detected in the absence of the riboprobe, demonstrating that the antibody reacts specifically with the synthetic RNA. Dataset 3 Expression of eomesa in the zebrafish pallium. Raw data of Figure 1 and additional image files of eomesa expression in the adult pallium. Dataset 4 Expression of emx1, emx2 and emx3 in the zebrafish larval brain. Raw data of Figure S3 and additional image files of emx gene expression in the zebrafish larvae. Dataset 5 Expression of emx1, emx2 and emx3 in the zebrafish pallium. Raw data of Figure 2 and additional image files of emx gene expression in the adult pallium. Dataset 6 Expression of Prox1 in the zebrafish pallium. Raw data of Figure 3 and additional image files of Prox1 expression in the adult pallium. Dataset 7 Expression of ascl1a in the zebrafish pallium. Raw data of Figure 4 and additional image files of ascl1a expression in the adult pallium. [file f1000research-3-7777-s0000.tgz › Fig4G_Ganzetal.tif]

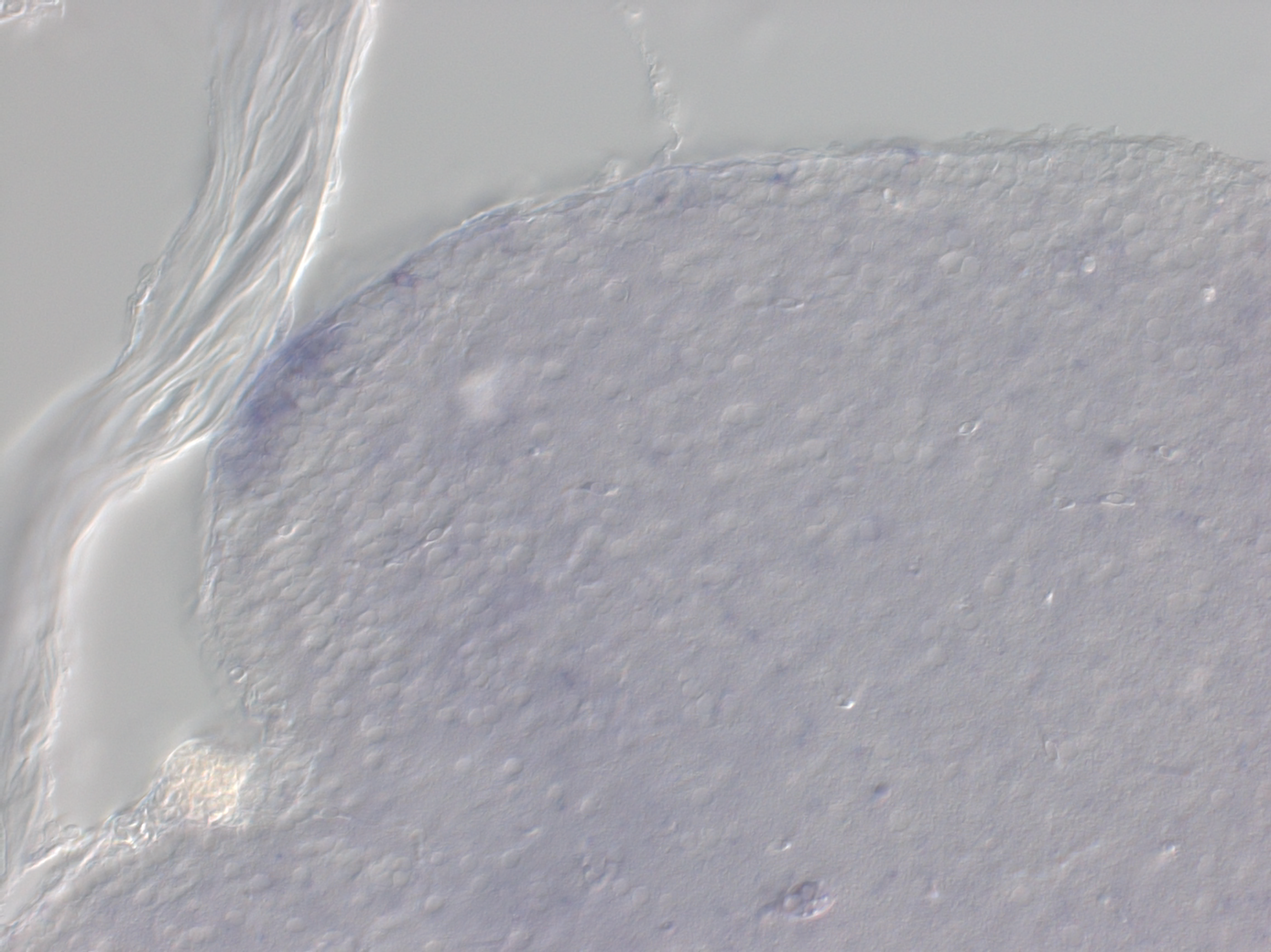

Supplement: Gene expression analysis in the adult zebrafish pallium — Dataset 1 Expression of eomesb in the embryonic brain and the adult pallium in zebrafish. Raw data of Figure S2 and additional image files of eomesb expression in the embryo and the adult pallium. Dataset 2 Images of negative control. No signal was detected in the absence of the riboprobe, demonstrating that the antibody reacts specifically with the synthetic RNA. Dataset 3 Expression of eomesa in the zebrafish pallium. Raw data of Figure 1 and additional image files of eomesa expression in the adult pallium. Dataset 4 Expression of emx1, emx2 and emx3 in the zebrafish larval brain. Raw data of Figure S3 and additional image files of emx gene expression in the zebrafish larvae. Dataset 5 Expression of emx1, emx2 and emx3 in the zebrafish pallium. Raw data of Figure 2 and additional image files of emx gene expression in the adult pallium. Dataset 6 Expression of Prox1 in the zebrafish pallium. Raw data of Figure 3 and additional image files of Prox1 expression in the adult pallium. Dataset 7 Expression of ascl1a in the zebrafish pallium. Raw data of Figure 4 and additional image files of ascl1a expression in the adult pallium. [file f1000research-3-7777-s0000.tgz › Fig4D_Ganzetal.TIF]

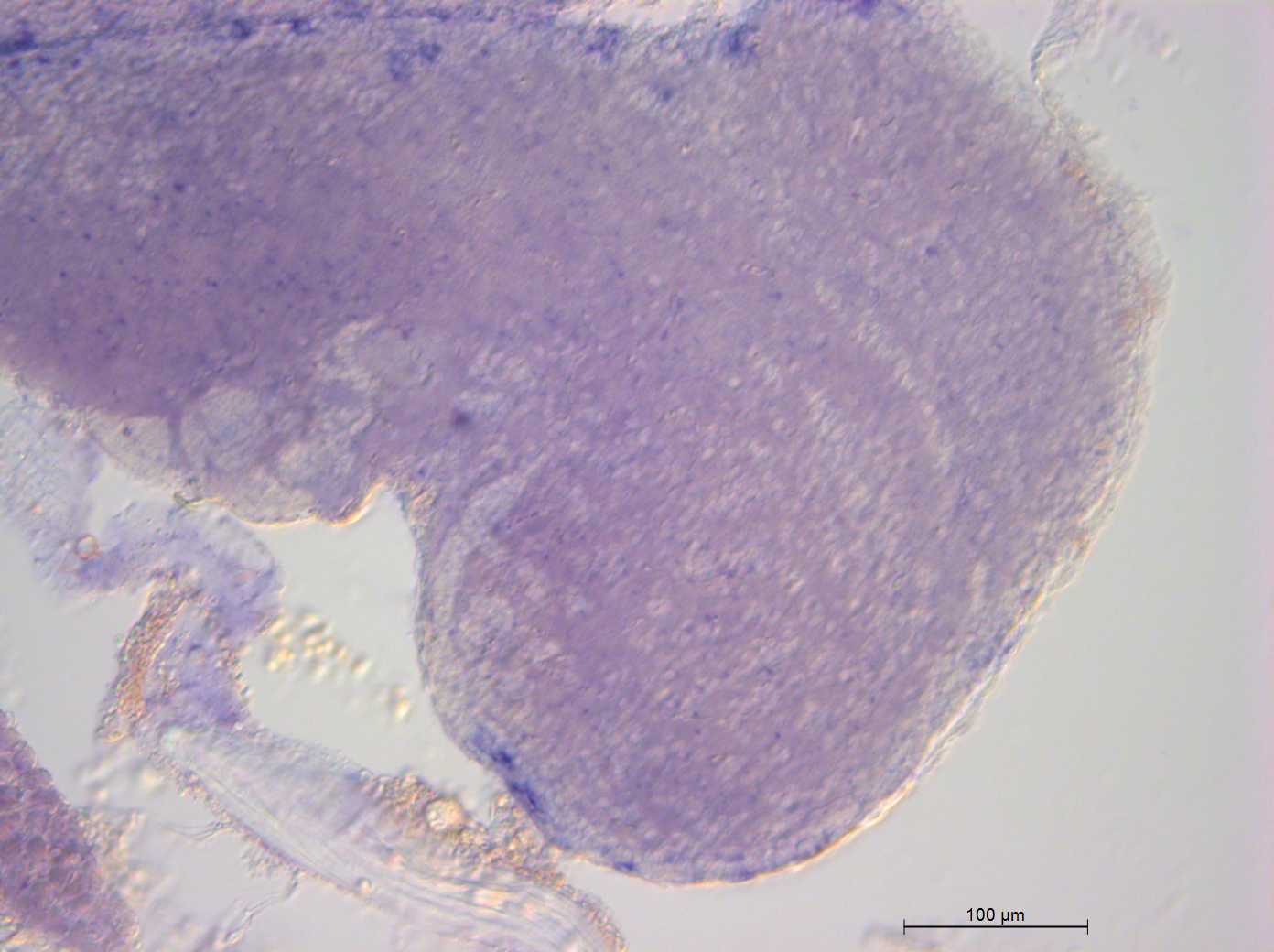

Supplement: Gene expression analysis in the adult zebrafish pallium — Dataset 1 Expression of eomesb in the embryonic brain and the adult pallium in zebrafish. Raw data of Figure S2 and additional image files of eomesb expression in the embryo and the adult pallium. Dataset 2 Images of negative control. No signal was detected in the absence of the riboprobe, demonstrating that the antibody reacts specifically with the synthetic RNA. Dataset 3 Expression of eomesa in the zebrafish pallium. Raw data of Figure 1 and additional image files of eomesa expression in the adult pallium. Dataset 4 Expression of emx1, emx2 and emx3 in the zebrafish larval brain. Raw data of Figure S3 and additional image files of emx gene expression in the zebrafish larvae. Dataset 5 Expression of emx1, emx2 and emx3 in the zebrafish pallium. Raw data of Figure 2 and additional image files of emx gene expression in the adult pallium. Dataset 6 Expression of Prox1 in the zebrafish pallium. Raw data of Figure 3 and additional image files of Prox1 expression in the adult pallium. Dataset 7 Expression of ascl1a in the zebrafish pallium. Raw data of Figure 4 and additional image files of ascl1a expression in the adult pallium. [file f1000research-3-7777-s0000.tgz › Fig4H_Ganzetal.tif]

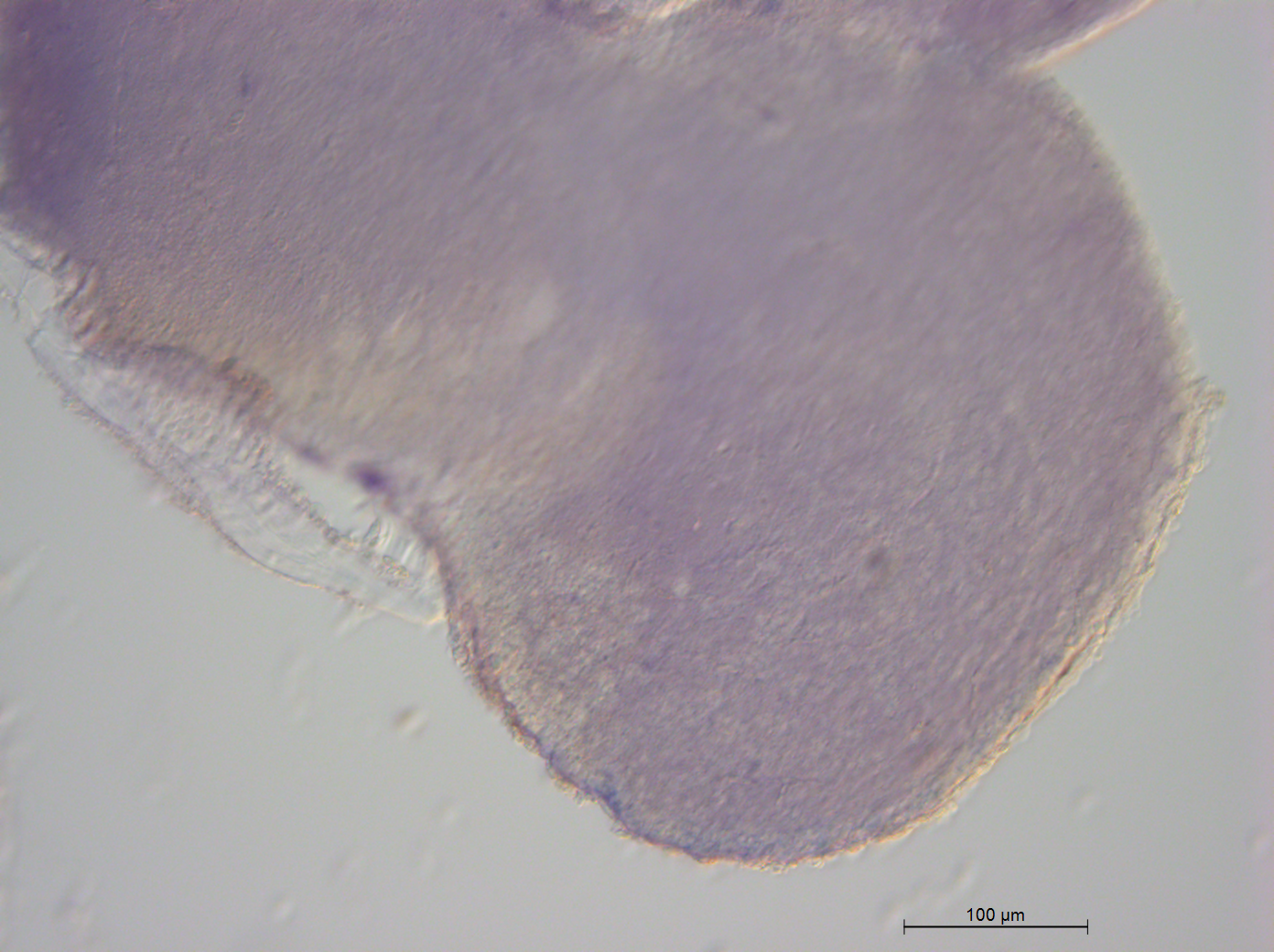

Supplement: Gene expression analysis in the adult zebrafish pallium — Dataset 1 Expression of eomesb in the embryonic brain and the adult pallium in zebrafish. Raw data of Figure S2 and additional image files of eomesb expression in the embryo and the adult pallium. Dataset 2 Images of negative control. No signal was detected in the absence of the riboprobe, demonstrating that the antibody reacts specifically with the synthetic RNA. Dataset 3 Expression of eomesa in the zebrafish pallium. Raw data of Figure 1 and additional image files of eomesa expression in the adult pallium. Dataset 4 Expression of emx1, emx2 and emx3 in the zebrafish larval brain. Raw data of Figure S3 and additional image files of emx gene expression in the zebrafish larvae. Dataset 5 Expression of emx1, emx2 and emx3 in the zebrafish pallium. Raw data of Figure 2 and additional image files of emx gene expression in the adult pallium. Dataset 6 Expression of Prox1 in the zebrafish pallium. Raw data of Figure 3 and additional image files of Prox1 expression in the adult pallium. Dataset 7 Expression of ascl1a in the zebrafish pallium. Raw data of Figure 4 and additional image files of ascl1a expression in the adult pallium. [file f1000research-3-7777-s0000.tgz › Fig4F_Ganzetal.tif]
