# Supplementary material for: Subdivisions of the adult zebrafish pallium based on molecular marker analysis
Source: F1000Res. 2015 Nov 4;3:308. Originally published 2014 Dec 17. [Version 2] doi: 10.12688/f1000research.5595.2 (PMC4335597; doi:10.12688/f1000research.5595.2)
Supplement: Peer review history

# Peer review: Referee response for version 1

**DOI:** 10.5256/f1000research.5978.r7077

**Published:** 2015-02-20

**Authors:** Steve Wilson

**Affiliations:** 1 Department of Cell and Developmental Biology, University College London, London, UK

In teleosts, the pallial telencephalon undergoes eversion during its development rather than the more common evagination that occurs in other vertebrates.  These differing modes of morphogenesis have made it difficult to resolve homologies between pallial domains in teleosts and other vertebrates.  This paper describes the pallial expression of zebrafish orthologues of genes that have helped to define pallial sub-domains in other vertebrates.   The methods are clearly described, the data are of high quality and the discussion of results are balanced and informative.  The authors acknowledge the limitations of using gene expression patterns and suggest additional experimental approaches that could be used to address the validity of hypotheses raised in the discussion of the data.   There is a great deal of information of use for comparative neuroanatomy studies and some observations challenge current models of teleost telencephalic organisation.  The expression analyses suggest that pallial sub-divisions show considerable variation from rostral to caudal telencephalon and it will be interesting to study how such organisation arises during development. I have no revisions that I consider to be essential.  I think that Monica Flogueira's concern about the summary figure potentially being misleading could most simply be addressed by adding a question mark after the suggested homology term.

I have read this submission. I believe that I have an appropriate level of expertise to confirm that it is of an acceptable scientific standard.

# Peer review: Referee response for version 1

**DOI:** 10.5256/f1000research.5978.r7076

**Published:** 2015-01-29

**Authors:** Perrti Panula

**Affiliations:** 1 Institute of Biomedicine, University of Helsinki, Helsinki, Finland

The title accurately describes the contents of the paper, and the abstract describes the findings and main conclusions well.The analysis of the selected markers in adult zebrafish telencephalon, particularly the pallium as is done here, is important for any functional studies on the zf brain.The study thus forms a good basis for further studies on adult fish brain. I have some comments on the controls below. Whereas the adult part is of high quality and will be very helpful in further analysis of e.g. circuits (which are essential to understand the functional significance of the results) and behavior, the developmental part could be improved.

Some detailed comments:

1. It needs to be indicated how the probe specificity has been verified. No probe-controls cannot replace a non-reacting probe control such as sense probe or partly scrambled probe controls, because occasionally probes bind non-specifically to e.g. proteins.
2. From the point of view of the conclusions regarding correlation to telencephalic areas of other species, the developmental part in the study could be improved. Although mentioned in the abstract, only a limited dataset is shown at 7 dpf (on p. 4, last chapter), and even here the data is difficult to find because the title indicates only analysis of adults.The age/stage of the samples is not indicated in Fig. S3, and the samples look earlier than 7 dpf. It would be particularly useful to see a series a developmental series in a set of images in which the brain domains could be easily identified. In this respect Fig.S3 is of limited value. The resolution is not very good, and clearly better figures could be provided. At least from 5 dpf on the brains are easily prepared from the skull and penetration is excellent. Adding data on the developmental expression series would significantly improve the paper. Whole brain ish can be easily done on even 1-month-old fish. The data during the whole first week, or (even better) two weeks would be useful.

In summary, the adult data is very useful and only a note on controls should be added. The developmental data could be improved to the same high level as the adult data.

I have read this submission. I believe that I have an appropriate level of expertise to confirm that it is of an acceptable scientific standard, however I have significant reservations, as outlined above.

# Peer review: Referee response for version 1

**DOI:** 10.5256/f1000research.5978.r7078

**Published:** 2015-01-08

**Authors:** Monica Folgueira

**Affiliations:** 1 Department of Cellular and Molecular Biology, University of A Coruña, A Coruña, Spain

Morphological differences between evaginated and everted telencephali hampers comparisons between brain morphotypes and complicates establishing homologies. This manuscript presents a comparative analysis of the expression pattern of a few transcription factors in the everted pallium of the zebrafish. These data will help us understand the organization of the zebrafish pallium and compare it with the pallium of other vertebrates. The manuscript will be of interest for readers working on comparative neuroanatomy and development, among other fields.

The manuscript is generally clear, well written and appropriate. The analysis of data is generally well performed, but maybe authors could clarify the following points:

- Based on their expression analysis, authors identify five subdivisions of the pallium that they name as Dm, Dlv, Dld, Dc and Dp after adapting nomenclature from Mueller *et al.* (2011). I think it could be clearer in the text how data supports the division between “Dc” and “Dld” at rostral levels. In addition, as authors show that “Dld” represents a different histogenic unit to “Dlv”, I wonder if “Dld” could be equivalent to “Dd/Dld” of other teleosts. Maybe authors could comment on this in the manuscript.
- *eomesa *and *Prox1 *seem to show differences between anterior and posterior regions of Dld. Yet, authors consider Dld as a continuous unit. Dc also seems to show differences from rostral to caudal levels (e.g. *emx2+* is only expressed in a caudal portion of Dc, while *eomesa* is only expressed in a rostral portion). Thus I wonder if more subnuclei could be identified in the zebrafish pallium, thus the organization of the pallium being even more complex. Maybe authors could comment on these observations (if correct).
- Authors discuss the divisions of the zebrafish pallium as corresponding to histogenic units of other vertebrates (ventral, lateral, dorsal and medial pallium). In addition, they propose certain homologies for specific nuclei, such as “Dm” being homologous to the “pallial amygdala” or “caudal part of Dld” homologous to the “dentate gyrus”. In my opinion, it is right to propose and discuss about such putative homologies in the manuscript. However, in my opinion, they are too speculative to be included in a summary figure, as this could mislead readers. I suggest removing these homologies from diagrams A-D in Figure 5, just referring to putative homologies for histogenic units.

**Minor comments:**

- Discussion: authors state that ‘Our gene expression data is consistent with the second part of the “modified pallial eversion model” of Mueller and colleagues’ To me, it is not clear what to authors mean by “second part of the modified pallial eversion model”.
- Figure 1C-D: ventricular expression is not clear in the original data sets.
- Figure 2C: Authors state that red lines indicate subdivisions based on the current marker. However, *emx2 *only seems to be labelling a central area at this telenphalic level (Dc). So, it does not seem to support the limits between Dm, Dl and Dp.
-  Figure S3: stages of development of the larvae are missing. Image C: in my opinion, OB limits are not accurately marked. Although the stage of development of the larva is not shown, the OB is certainly bigger than labelled between 3-5dpf (it seems correctly marked but not labelled in E-D).
- I find the abbreviation TGD unnecessary.

I have read this submission. I believe that I have an appropriate level of expertise to confirm that it is of an acceptable scientific standard.

# Response

**Published:** 2015-10-24

**Authors:** Julia Ganz

**Affiliations:** University of Oregon, USA

We thank Monica Folgueira for her helpful comments and suggestions.

- Based on their expression analysis, authors identify five subdivisions of the pallium that they name as Dm, Dlv, Dld, Dc and Dp after adapting nomenclature from Mueller *et al.* (2011). I think it could be clearer in the text how data supports the division between “Dc” and “Dld” at rostral levels.

The difference between Dc and Dld is the absence of *emx3* positive cells in Dld. We have included this point in the discussion to make it clearer and now write:

“Dld is different from Dc based on absence of *emx3* positive cells in Dld.”

- In addition, as authors show that “Dld” represents a different histogenic unit to “Dlv”, I wonder if “Dld” could be equivalent to “Dd/Dld” of other teleosts. Maybe authors could comment on this in the manuscript.

The unit ‘Dld” could be an equivalent to “Dd/Dld” of other teleosts (such as in trout). As there are, however, to our knowledge, no good markers that have been used to identify “Dd/Dld”, we do not want to introduce another subdivision that has not been used so far in zebrafish. It would be really interesting though to test if our proposed subdivision “Dld” is equivalent to “Dd/Dld”.

- *eomesa *and *Prox1 *seem to show differences between anterior and posterior regions of Dld. Yet, authors consider Dld as a continuous unit. Dc also seems to show differences from rostral to caudal levels (e.g. *emx2+* is only expressed in a caudal portion of Dc, while *eomesa* is only expressed in a rostral portion). Thus I wonder if more subnuclei could be identified in the zebrafish pallium, thus the organization of the pallium being even more complex. Maybe authors could comment on these observations (if correct).

We have included different color coding in our summary figure to reflect these changes in gene expression (light green – medium green – dark green for Dc, and light yellow – dark yellow for Dld). We have also added an explanation to this color code in the figure legend and write:

“The different shades of green (Dc) and yellow (Dld) indicate gene expression changes along the rostro-caudal axis.”

At this point, we have avoided adding additional subdivision acronyms as they might be potentially confusing especially when comparing the subdivisions in zebrafish to other teleost species.

- Authors discuss the divisions of the zebrafish pallium as corresponding to histogenic units of other vertebrates (ventral, lateral, dorsal and medial pallium). In addition, they propose certain homologies for specific nuclei, such as “Dm” being homologous to the “pallial amygdala” or “caudal part of Dld” homologous to the “dentate gyrus”. In my opinion, it is right to propose and discuss about such putative homologies in the manuscript. However, in my opinion, they are too speculative to be included in a summary figure, as this could mislead readers. I suggest removing these homologies from diagrams A-D in Figure 5, just referring to putative homologies for histogenic units.

We have adopted the suggestion from the reviewer Steve Wilson to add a question mark to figure 5 (“Putative homologous region in the tetrapod pallium (?)”) to indicate that the putative homologies that we propose based on our work will need additional studies. We also point this out both in the figure legend of figure 5 and at the beginning of the discussion.

**Minor comments:**

- Discussion: authors state that ‘Our gene expression data is consistent with the second part of the “modified pallial eversion model” of Mueller and colleagues’ To me, it is not clear what to authors mean by “second part of the modified pallial eversion model”.

We have clarified the text and now write:

“Our gene expression data is consistent with the “modified partial pallial eversion model” of Mueller and colleagues with regard to Dc.” 

- Figure 1C-D: ventricular expression is not clear in the original data sets.

The cells at caudal levels of the telencephalon are scattered and a bit weaker labeled than at rostral levels, but there are scattered *eomesa *positive cells present at the ventricular zone of Dm at all levels.

- Figure 2C: Authors state that red lines indicate subdivisions based on the current marker. However, *emx2 *only seems to be labelling a central area at this telencephalic level (Dc). So, it does not seem to support the limits between Dm, Dl and Dp.

We have modified this and changed the red lines in the figure. 

-  Figure S3: stages of development of the larvae are missing. Image C: in my opinion, OB limits are not accurately marked. Although the stage of development of the larva is not shown, the OB is certainly bigger than labelled between 3-5dpf (it seems correctly marked but not labelled in E-D).

We have included the stages of development in the figure legend. We have included the OB label in S3E,D. We have changed the OB limits in the figure S3C accordingly.

- I find the abbreviation TGD unnecessary.

The abbreviation has been removed.
